# Efficacy and safety of invasive laser acupuncture (650 and 830 nm) on knee osteoarthritis: A pilot randomized clinical trial

**DOI:** 10.1371/journal.pone.0353654

**Published:** 2026-07-20

**Authors:** Raeon Jang, Changsop Yang, Jae-Hong Kim, Dongwoo Nam, Jeong-Cheol Shin, Gwang-Cheon Park, Byoung-Kab Kang, Kyung-Min Shin, Ae-Ran Kim

**Affiliations:** 1 Clinical Research Center, Dongshin University Gwangju Korean Medicine Hospital, Gwangju, Republic of Korea; 2 Department of Clinical Korean Medicine, Graduate School, Kyung Hee University, Seoul, Republic of Korea; 3 KM Science Research Division, Korea Institute of Oriental Medicine, Daejeon, Republic of Korea; 4 Department of Acupuncture and Moxibustion Medicine, College of Korean Medicine, Dongshin University, Naju, Republic of Korea; 5 Department of Acupuncture and Moxibustion, College of Korean Medicine, Kyung Hee University, Seoul, Republic of Korea; 6 Clinical Research Coordinating Team, Korea Institute of Oriental Medicine, Daejeon, Republic of Korea; Yarmouk University, JORDAN

## Abstract

This pilot study explored the safety and efficacy of invasive laser acupuncture (ILA) using two wavelengths (650 and 830 nm) compared with a sham laser in patients with knee osteoarthritis (KOA). This randomized, parallel-arm, patient- and outcome assessor-blinded, placebo-controlled, single-center clinical trial was designed with three groups (control, 650, and 830 nm), and 45 participants were randomly allocated in a ratio of 1:1:1. All participants were treated with ILA on the more affected side at SP9, SP10, ST34, ST35, EX-LE2, EX-LE4, and GB34 for 10 minutes per session, twice weekly, for a total of 12 sessions over 6 weeks. The primary outcome was the change in pain visual analog scale (VAS) score at rest and during activity at baseline (Visit 1) versus 1 week after intervention completion (Visit 13). Secondary outcomes included VAS scores after six treatments (Visit 7) and 6 weeks after intervention completion (Visit 14), Western Ontario and McMaster Universities Osteoarthritis Index (WOMAC) scores, European Quality of Life Five Dimension Five Level Scale (EQ-5D-5L), patient’s global assessment, rescue medication dosage, and responder rate. Changes in the VAS scores at rest (p = 0.0044, Visit 13; p = 0.0001, Visit 14) and during activity (p = 0.0038, Visit 13; p < 0.0001, Visit 14) were significantly different among the three groups. Significant differences were also observed in WOMAC total (p = 0.0278, Visit 7; p = 0.0485, Visit 13; p = 0.0051, Visit 14), pain (p = 0.0073, Visit 7; p = 0.0210, Visit 13; p = 0.0038, Visit 14), function subscale (p = 0.0055, Visit 14), and EQ-5D-5L scores (p = 0.0131, Visit 14). No ILA-related adverse events or significant changes in vital signs or laboratory results were observed during the study. ILA at 650 and 830 nm showed preliminary signals of short-term pain reduction. However, these preliminary findings should be confirmed in larger, rigorously designed clinical trials.

**Trial registration:**

CRIS registration number: KCT0008860; registered on 10/11/2023 (https://cris.nih.go.kr/cris/search/detailSearch.do/25506).

## Introduction

Knee osteoarthritis (KOA) is a prevalent joint disease involving new bone formation, focal cartilage loss, and bone enlargement. KOA can lead to usage-related pain and/or functional limitation. Therefore, people with KOA are at risk of mobility disability [1]. KOA was consistently ranked among the ten most frequently hospitalized diseases in Korean medicine from 2013 to 2021. Furthermore, the number of patients, number of visitation days, and total amount of medical care costs in Korean medicine hospitalizations have steadily increased during the last three years (2021–2023) [2]. Therefore, KOA incurs significant social and economic costs.

According to the American College of Rheumatology (ACR) guideline for the management of osteoarthritis, non-pharmacologic therapies such as exercise, weight loss, tai chi, and acupuncture and pharmacologic therapies such as acetaminophen, oral non-steroidal anti-inflammatory drugs (NSAIDs) and topical NSAIDs can be considered for the management of KOA [3]. If the symptoms persist and cannot be relieved by non-operative treatment, operative treatments, such as total knee replacement arthroplasty (TKRA), may be considered [4].

Low-level laser (LLL) therapy, a non-invasive treatment, is known to relieve pain and improve function through its anti-inflammatory effect [5,6]. Some systematic reviews and meta-analyses have reported that LLL may be effective in treating KOA [7–9]. Laser acupuncture (LA), a branch of non-invasive LLL therapy, stimulates acupoints through the logic of traditional Chinese medicine by LLL [10]. Several systematic reviews have suggested that LA improves pain and neuropathy [11,12]. Invasive laser acupuncture (ILA) combines acupuncture—a modality conditionally recommended for KOA in the ACR guideline [3]—with low-level laser therapy. It is performed by invasive irradiation and acupuncture stimulation using an optical fiber-coupled laser diode (InGaAlP 650 nm; GaAlAs 830 nm) inserted inside the needle (material, stainless steel; length, 30 mm; inner diameter, 0.15 mm; external diameter, 0.3 mm). A previous study demonstrated that the 650 nm ILA restricted the formation of inflammatory mediators in osteoarthritis [13]. Additionally, ILA for chronic non-specific low back pain can reduce pain and improve functional disability [14].

However, no previous studies have evaluated ILA for KOA. Therefore, this exploratory pilot study aimed to explore wavelength-dependent differences in the safety and efficacy of ILA (650 and 830 nm) compared with a sham laser control in patients with KOA. This pilot study provides a basis for future research on the use of ILA in KOA.

## Materials and methods

The study design and reporting followed the Standard Protocol Items of the Recommendations for Interventional Trials (SPIRIT) statements [15] and Consolidated Standards of Reporting Trials (CONSORT) guidelines [16]. The methodology of this study has been reported in a previous study [17].

### Study design

We conducted a randomized, parallel-arm, patient- and outcome assessor-blinded, placebo-controlled, single-center prospective pilot clinical trial (Dongshin University Gwangju Korean Medicine Hospital, Republic of Korea). A total of 45 participants who were judged eligible for the study were randomly assigned to the 650 group (650 nm wavelength ILA), 830 group (830 nm wavelength ILA), or control group (Sham ILA) at a ratio of 1:1:1. The participants were treated with ILA on one-side (more affected side) of SP9 (Yinglingquan; Spleen 9), SP10 (Xuehai; Spleen 10), ST34 (Liangqiu; Stomach 34), ST35 (Dubi; Stomach 35), EX-LE2 (Heding; Extra Lower Extremity 2), EX-LE4 (Neixiyan; Extra Lower Extremity 4), and GB34 (Yanglingquan; Gallbladder 34) for 10 minutes per session, twice weekly, for a total of 12 sessions over 6 weeks. Measurements for the outcomes, except the patient’s global assessment (PGA) and dosage of rescue medication, were carried out at baseline (Visit 1), after six treatments (Visit 7), 1 week after intervention completion (Visit 13), and 6 weeks after intervention completion (Visit 14). Other variables were measured at Visits 7, 13, and 14. A summary of this study is presented in Table 1.

**Table 1:** Standard protocol items: Recommendations for interventional trials (SPIRIT) statement.

	STUDY PERIOD							
	Enrolment	Allocation	Post-allocation					Close-out
TIMEPOINT	Screening		Visit1	Visit2–6	Visit7	Visit8–12	Visit13	Visit14
	Week		1	1-3	4	4-6	7	12
ENROLMENT								
Informed consent	X							
Sociodemographic profile	X							
Medical history	X							
								
Inclusion/exclusion criteria	X							
Allocation		X						
INTERVENTIONS								
ILA (sham, 650-nm, 830-nm)			X	X	X	X		
Self-management and exercise teaching			X	X	X	X		
ASSESSMENTS								
Change of medical history			X	X	X	X	X	X
Vital signs	X	X	X	X	X	X	X	X
Laboratory test	X						X	
VAS	X		X		X		X	X
WOMAC			X		X		X	X
PGA					X		X	X
EQ-5D-5L			X		X		X	X
Check dosage of rescue medication					X		X	X
Check adverse events			X	X	X	X	X	X
								

VAS, visual analog scale; ILA, invasive laser acupuncture; WOMAC, Western Ontario and McMaster Universities Osteoarthritis Index; PGA, Patient’s Global Assessment; EQ-5D-5L: European Quality of Life Five Dimension Five Level Scale

### Ethical considerations

The research was performed in line with the Declaration of Helsinki. The study protocol was approved before the trial’s start by the Institutional Review Board (IRB) of Dongshin University Gwangju Korean Medicine Hospital (approval no: DSGOH-2023–002; approval date: July 18, 2023). This research was registered with the Clinical Research Information Service of the Republic of Korea (cris.nih.go.kr; Registration No. KCT0008860; registered date: October 11, 2023; available at https://cris.nih.go.kr/cris/search/detailSearch.do/25506). All the participants were informed of the purpose, background, and precautions of the study, and written informed consent was obtained prior to the start of the trial. The authors confirm that all ongoing and related trials for this intervention are registered. All investigators participating in the trial had no conflicts of interest.

### Participant recruitment

Participants were enrolled at the Dongshin University Gwangju Korean Medicine Hospital, Republic of Korea. Participants were recruited through community posters from October 4, 2023 to April 1, 2024 and were treated and followed up from October 10, 2023 to June 26, 2024. The investigator explained about the purpose, background, and precautions for the study. Subsequently, the participants signed an informed consent form before participation. The clinical research coordinator (CRC) checked for the adverse events (AEs) and coordinated the study schedule during the study period.

### Participants

The inclusion criteria were as follows: (1) individuals aged between 50 and 85 years; (2) diagnosed with KOA using the ACR diagnostic criteria [18]; (3) experiencing knee pain for more than 14 days per month and persisting for more than the 3 months prior to enrollment; (4) existence of moderate knee pain (daily mean pain 0–100 mm visual analogue scale [VAS] score during activity and at rest over the past week up to screening between 35 and 74); (5) diagnosis with Kellgren-Lawrence (K-L) grade 2 or 3 by x-ray [19] and (6) providing informed consent

The exclusion criteria were as follows: (1) individuals having TKRA due to KOA; (2) individuals with KOA in post-traumatic state; (3) serious underlying diseases (severe renal, cardiovascular, central nervous system disease, diabetic neuropathy and cancer); (4) knee pain caused by inflammatory disease, tumor, trauma, rheumatoid arthritis, autoimmune disease, severe genu varum or genu valgum, severe hip joint disease, or gout; (5) history of treatment for drug or alcohol dependence or mental illnesses (dementia, schizophrenia, or epilepsy) in the 6 months prior to screening; (6) injection with intra-articular mucosal supplement (hyaluronan, sodium hyaluronate, and hylan) for the treatment of KOA in the 6 months prior to screening; (7) aspiration of knee joint fluid by intra-articular injection in the 3 months prior to screening; (8) prolotherapy or steroid injections into or near the knee joint in the 3 months prior to screening; (9) current treatment such as Korean medicine treatment, non-drug local treatment (physical therapy), or medication (including health functional food) for KOA in the 4 weeks prior to screening; (10) undergoing treatment for pain control (e.g., steroids, NSAIDs) due to other diseases; (11) unsuitable case application of ILA, such as blood clotting disability, metallic devices in the knee, severe skin disease about the treatment area, or electronic medical devices; (12) knee surgery within 1 year or set to have during the trial; (13) women who were pregnant, preparing for pregnancy, or breastfeeding; and (14) individuals who have participated in other trials within the 2 months before screening or were currently participating in other trials

The dropout criteria were as follows: (1) withdrawal of informed consent for participants, (2) occurrence of serious AEs in participants; (3) absence of efficacy data after allocation (i.e., intervention was not conducted or absence of baseline assessments); and (4) judgment by the investigator or IRB that exclusion of the participant from the trial was appropriate

The violation criteria were as follows: (1) investigators or participants remarkably violated the trial protocol, and (2) treatment compliance rate of less than 75%

### Randomization and blinding

The 45 eligible participants were randomly assigned to three groups (control [0 mW power sham laser], 650 [20 mW power, 650 nm wavelength laser], or 830 [20 mW power, 830 nm wavelength laser]) at a 1:1:1 ratio. The randomization sequence was generated by SAS^Ⓡ^ (Version 9.4, SAS Institute, Cary, NC, USA) with block randomization by a statistician who did not participate in the trial. Allocation concealment was ensured through the following procedure. Each allocation code was placed inside an opaque envelope, which was then sealed and labeled with a sequential number. The envelopes were stored in a double-locked cabinet. At the point of enrollment—immediately after a participant had completed baseline measurements and provided informed consent—a designated randomization administrator, who took no part in screening and intervention, outcome assessment, or data analysis, retrieved the next envelope in numerical order, opened it, and recorded the date and signature on a log sheet. The group assignment was disclosed only to the practitioner who delivered the intervention.

Because of the nature of the ILA treatment, in which the practitioner needed to operate the laser device (sham, 650 nm, or 830 nm laser) after inserting the needle, blinding of the practitioner was not feasible. To minimize potential bias, we conducted a patient- and assessor-blinded trial. Participants could not feel any specific sensation distinguishing between the real laser and sham laser, and the device was configured to generate identical operational noise across all groups. In addition, participants wore a blindfold during the procedure to block visual identification of the intervention. Through these methods, participants were unable to distinguish between treatment conditions. The outcome assessor was not in contact with any participant except during the assessment time points. Thus, until trial completion, both participants and assessor were blinded to the group allocation. Formal assessment of blinding effectiveness using a validated blinding index was not performed in this pilot trial.

### Intervention

The participants were treated with ILA on one-side (more affected side) SP9, SP10, ST34, ST35, EX-LE2, EX-LE4, and GB34, which were based on previous randomized controlled trial (RCT) and clinical practice guidelines for KOA [20,21], for 10 minutes per session, twice weekly, for a total of 12 sessions over 6 weeks. Participants were placed in the supine position and wore a blindfold to block their sight. Certified Korean medicine doctors who had completed 6 years of formal university training to practice Korean medicine performed ILA using a medical device (Ellise, WONTECH Co.Ltd.) composed of a combination of a sterile, disposable acupuncture needle (material, stainless steel; length, 30 mm; inner diameter, 0.15 mm; external diameter, 0.3 mm), optical fiber-coupled laser diode (InGaAlP 650 nm; GaAlAs 830 nm), and LLL (power, 20 mW; energy density, 38216.56 J/cm^2^; energy dose, 12 J/point; power density, 63.69 W/cm^2^; frequency 50 Hz) (S1 Fig). Once the needles were vertically inserted at the acupoints, laser irradiation treatment was initiated according to group allocation. The control group was treated with sham LLL, which involved acupuncture without irradiation at a power of 0 mW. The methods of ILA, aligning with the Revised Standards for Reporting Intervention in Clinical Trials of Acupuncture guidelines, are detailed in S1 Table.

Rescue medication (acetaminophen 500 mg, 20 tablets) was provided to all participants at Visit 1 and Visit 13. Participants were instructed to take the medication only when their knee pain was intolerable, at a dosage of 1–2 tablets (500–1,000 mg) per intake at intervals of 4–6 hours, and limited to a maximum of 8 tablets (4,000 mg) per day. Dosage of rescue medication was monitored at Visits 7, 13, and 14.

Education on self-care and exercise was conducted on each intervention visit.

During the study period, any treatment related to the alleviation of KOA symptoms, other than our intervention, was not permitted.

### Outcome measurements

The primary outcome was the difference in changes in the VAS scores at rest and during activity (while walking on flat ground) among the three groups at visit 13.

Secondary outcomes were the VAS scores (including VAS scores at rest and during activity) at Visits 7 and 14, Korean version of the Western Ontario and McMaster Universities Osteoarthritis Index (WOMAC) total, WOMAC pain subscale, WOMAC function subscale, European Quality of Life Five Dimension Five Level Scale (EQ-5D-5L), Patient Global Assessment (PGA) score, dosage of rescue medication, and responder rate. These were measured at Visits 1, 7, 13, and 14. However, PGA and rescue medication dosage were excluded at Visit 1 and responder rate was only measured at Visit 13.

VAS, which conveniently describes the pain, is a 100-mm-long scale with two ends, “no pain” to “pain as ever been never” [22]. It is useful in identifying changes in degenerative joint pain [23].

WOMAC is a useful index for assessing the severity of knee pain and disability. It is composed of 24 questions, including 5 questions for pain, 17 questions for function, and 2 questions for stiffness [23]. In our trials, we used the Korean WOMAC, which has been verified for its reliability and validity [24].

The EQ-5D-5L is designed to evaluate health statements in five dimensions (mobility, self-care, usual activities, pain/discomfort, and anxiety/depression) at five levels (1–5) [25].

The PGA is an indicator that assesses a patient’s perception of treatment. Regarding treatment satisfaction, very poor was set to 1, very good to 5, and one from 1 to 5 was selected [26].

We defined responders based on the OMERACT-OARSI criteria. A responder was defined by any of the following categories: (1) improvement in the WOMAC pain scale or function scale of more than 50% and absolute change of more than 20 out of 100, and (2) improvement comprising at least two of the following: WOMAC pain subscale score of 20% and absolute change of 10 or more. WOMAC function subscale score of 20%, and the absolute change of 10 or more. PGA score of 20%, with an absolute change of 10 or more [27].

### Sample size calculation

We adopted a pilot study design due to the absence of previous studies, limited budgets, and enrollment opportunities. In a three-arm pilot study, the adequate sample size is more than 12 [28,29]. Assuming that 20% of the participants may drop out, we allocated 15 participants to each group.

Because this study was a pilot study, the sample size was not enough to evaluate the efficacy and safety of ILA for KOA. However, our pilot study may establish the feasibility of a larger, rigorously designed confirmatory RCT on ILA in patients with KOA and provide preliminary insight into the acceptability and safety of ILA for KOA patients.

### Statistical analyses

Outcomes were analyzed using SAS^Ⓡ^ (Version 9.4, SAS Institute, Cary, NC, USA). The data obtained from the participants were analyzed in two ways: a safety set and a full analysis set. In case of missing data, they were applied to the last observation carried forward (LOCF), which replaced the missing data with the last obtained data. The data obtained from this clinical trial are presented using appropriate descriptive statistics. For continuous variables, mean ± standard deviation or medians, with interquartile are presented. For categorical variables, frequencies or percentages are presented. The significance of differences was verified using a two-tailed test at a 5% significance level.

Regarding demographic characteristics, continuous variables were tested by one-way analysis of variance (ANOVA) or the Kruskal–Wallis test and categorical variables were tested using Fisher’s exact test.

In the outcome analyses and safety tests, differences in changes among the three groups were assessed using a normality test. Parametric or non-parametric tests were conducted.

For the outcome analyses, the difference in the changes among the three groups was tested using analysis of covariance (ANCOVA) with BMI included as a covariate. In case of responder rate, we used the Chi-square test (χ²).

For the safety test, we used ANOVA or the Kruskal–Wallis test for differences in the changes among the three groups. Additionally, the Fisher’s exact test was used for AEs.

Subanalyses and interim analyses were not conducted

## Results

### Participants

**Fig 1:**
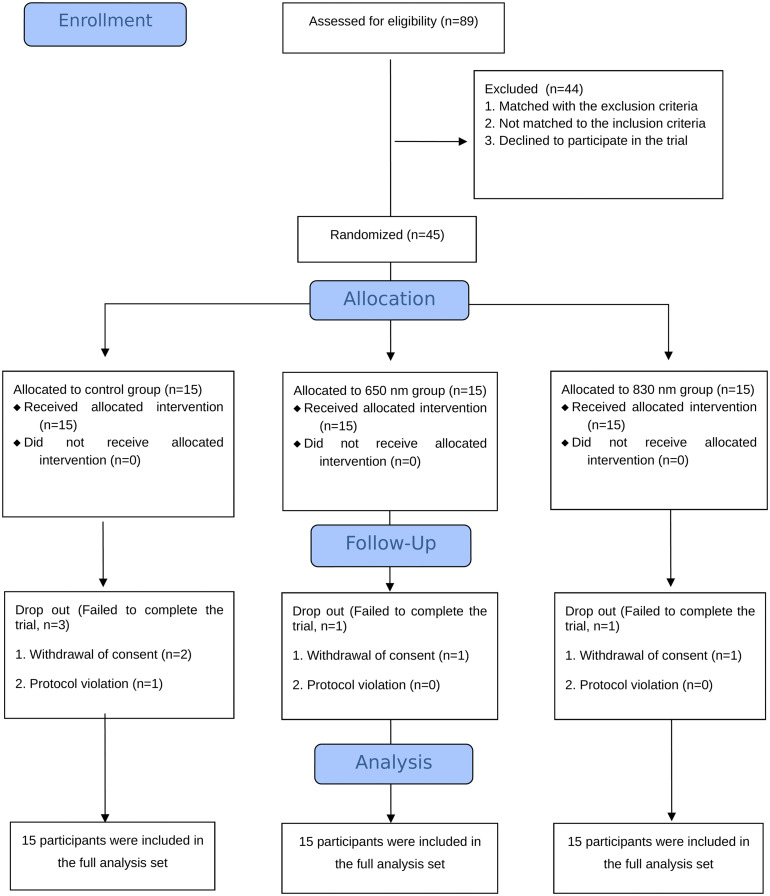
CONSORT 2010 Flow Diagram.

During the screening, of the 89 participants evaluated for eligibility, 44 were excluded. As a result, 45 participants were accepted as trial participants and were randomly divided into three groups (control group, 650 group, 830 group) of 15 each. During the treatment phase, 5 participants failed to finish the treatment with three dropouts in the control group, one in the 650 group, and one in the 830 group (Fig 1).

### Baseline characteristics

The demographic characteristics of the three groups are summarized in Table 2. No significant differences were observed among the three groups, except for body mass index (BMI; p = 0.0072).

**Table 2:** Baseline characteristics of participants at baseline.

Dependent Variables	Control (n = 15)	650 (n = 15)	830 (n = 15)	P-Value
Age (y)	69.7 (4.4)	67.9 (6.4)	70.7 (3.6)	0.3010^†^
Sex (Female)	13 (86.67%)	15 (100%)	14 (93.33%)	0.7622^*^
BMI (kg/m^2^)	23.8(22.4, 24.9)	26.3(23.5, 28.4)	23.8(20.4, 24.8)	**0.0072** ^**#**^
Smoking (No)	15 (100%)	15 (100%)	15 (100%)	ND
Drinking (No)	14 (93.33%)	14 (93.33%)	14 (93.33%)	1.0000^*^
Exercise (hour/week)	6.0 (4.0, 10.0)	5.0 (5.0, 7.0)	5.0 (3.0, 8.0)	0.6695^**#**^
VAS at rest	49.0 (40.0, 50.0)	49.0 (39.0, 50.0)	49.0 (40.0, 50.0)	0.9842^**#**^
VAS during activity	50.0 (45.0, 56.0)	49.0 (40.0, 51.0)	46.0 (40.0, 59.0)	0.7989^**#**^
WOMAC total	67.8 (18.2)	61.7 (13.7)	63.2 (11.5)	0.5007^†^
WOMAC Pain subscale	13.7 (3.9)	12.8 (3.2)	12.3 (2.9)	0.5483^†^
WOMAC function subscale	48.5 (13.7)	43.6 (10.1)	45.7 (8.0)	0.4742^†^
WOMAC stiffness subscale	6.0 (5.0, 7.0)	6.0 (4.0, 6.0)	5.0 (4.0, 6.0)	0.3323^**#**^
EQ-5D-5L	0.74 (0.66, 0.80)	0.75 (0.68, 0.80)	0.73 (0.68, 0.75)	0.6371^**#**^

Values are expressed as means (standard deviation), medians (Q1, Q3) or counts (%)

†, p-value for one-way ANOVA; #, p-value for Kruskal–Wallis test, *, p-value for Fisher’s exact test.

ANOVA, analysis of variance; BMI, body mass index; VAS, visual analog scale; WOMAC, Western Ontario and McMaster Universities Osteoarthritis Index; EQ-5D-5L, European Quality of Life Five Dimension Five Level Scale; Q1, first quartiles; Q3, third quartiles.

### Efficacy of the outcomes

To account for the baseline imbalance in BMI, we conducted ANCOVA for all outcome measures, using BMI as a covariate to minimize potential bias and ensure the robustness of the findings.

**Fig 2:**
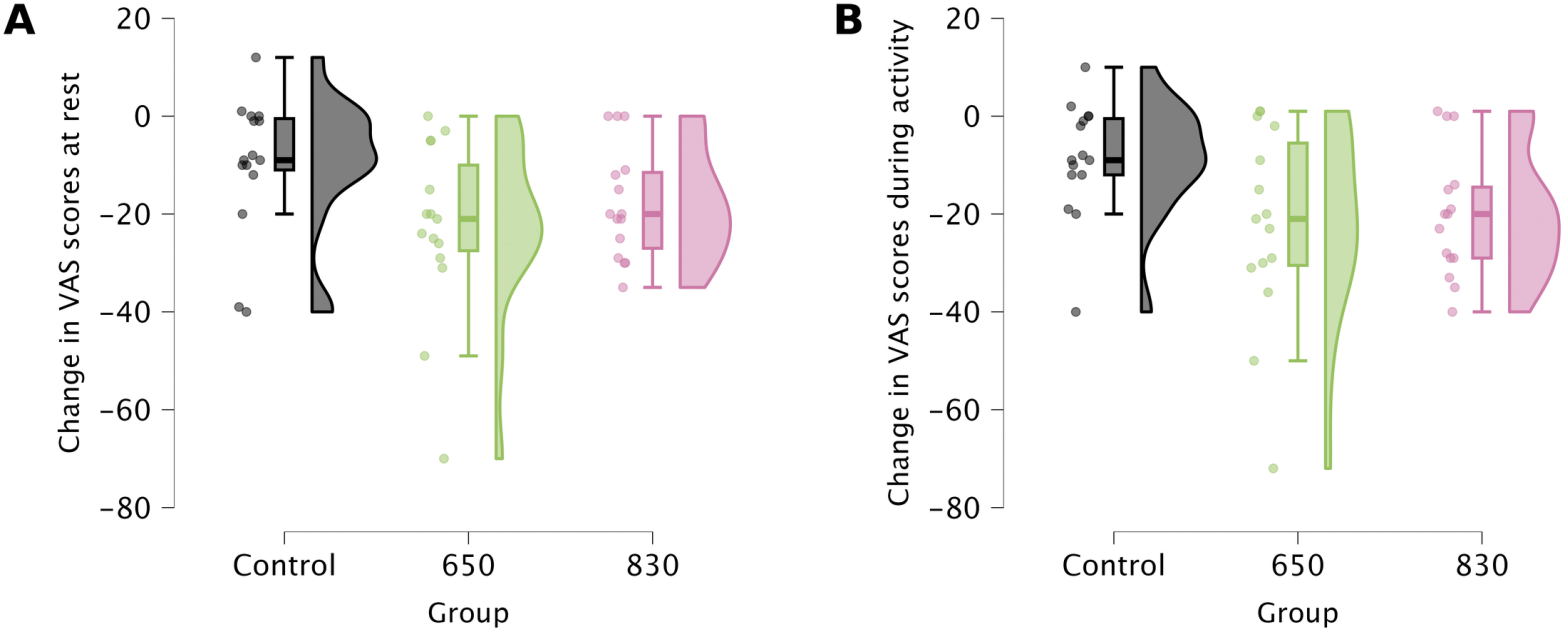
Raincloud plots of the change in VAS scores between Visit 13 and Visit 1. (A) VAS at rest; (B) VAS during activity. For each group (control, 650 nm, and 830; n = 15 per group), individual participant values (left), a box plot showing the median and interquartile range with whiskers (center), and a half-violin density estimate (right) are presented. More negative values indicate greater pain reduction. VAS, visual analog scale.

After completion of the intervention and 6 weeks later, we observed significant differences in changes in the VAS scores both at rest (p = 0.0044, Visit 13 – Visit 1; p = 0.0001, Visit 14 – Visit 1) and during activity (p = 0.0038, Visit 13 – Visit 1; p < 0.0001, Visit 14 – Visit 1) among the three groups (Table 3). The distributions of individual changes in the VAS scores at the primary endpoint (Visit 13) are shown in Fig 2, and the detailed scores at Visits 1, 7, 13, and 14 are provided in S2A Table.

**Table 3:** Changes in the VAS scores (Visit 7 vs. Visit 1, Visit 13 vs. Visit 1, Visit 14 vs. Visit 1) in the three groups.

Dependent Variable	Group	Difference (v7-v1)	P-value	Difference (v13-v1)	P-value	Difference (v14-v1)	P-value
VAS at rest	Control (n = 15)	−5.0 (−9.5, −0.6)	0.0733	−7.2 (−14.0, −0.3)	**0.0044**	−6.8 (−13.0, −0.0)	**0.0001**
	650 (n = 15)	−9.6 (−14.5, −4.8)		−20.7 (−28.2, −13.2)		−23.4 (−30.8, −16.0)	
	830 (n = 15)	−12.2 (−16.8, −7.6)		−22.6 (−29.7, −13.2)		−28.0 (−35.0, −21.0)	
VAS during activity	Control (n = 15)	−4.7 (−10.9, −1.6)	0.0781	−6.7 (−13.9, −0.5)	**0.0038**	−5.6 (−13.0, −1.9)	**<0.0001**
	650 (n = 15)	−10.0 (−16.9, −3.0)		−21.7 (−29.7, −13.7)		−22.3 (−30.6, −14.1)	
	830 (n = 15)	−14.7 (−21.3, −8.3)		−22.9 (−30.4, −15.4)		−31.3 (−39.1, −23.5)	

Values are expressed as least square adjusted means (95% confidence interval)

P-value for ANCOVA test adjusted baseline and BMI

VAS, visual analog scale

Additionally, the WOMAC total score (p = 0.0278, Visit 7 – Visit 1; p = 0.0485, Visit 13 – Visit 1; p = 0.0051, Visit 14 – Visit 1), WOMAC pain subscale (p = 0.0073, Visit 7 – Visit 1; p = 0.0210, Visit 13 – Visit 1; p = 0.0038, Visit 14 – Visit 1), WOMAC function subscale (p = 0.0055, Visit 14 – Visit 1), and EQ-5D-5L (p = 0.0131, Visit 14 – Visit 1) were significantly different among the three groups (Table 4). The detailed scores at Visits 7, 13, and 14 are provided in S2A Table.

**Table 4:** Changes in WOMAC scores and EQ-5D-5L (Visit7 vs. Visit1, Visit13 vs. Visit1, Visit14 vs. Visit 1) in the three groups.

Dependent Variable	Group	Difference (v7-v1)	P-value	Difference (v13-v1)	P-value	Difference (v14-v1)	P-value
WOMAC total	Control (n = 15)	−5.7 (−11.0, −0.3)	**0.0278**	−8.9 (−16.8, −1.0)	**0.0485**	−9.3 (−17.2, −1.5)	**0.0051**
	650 (n = 15)	−10.6 (−16.5, −4.6)		−22.0 (−30.7, −13.4)		−24.5 (−33.0, −15.9)	
	830 (n = 15)	−15.9 (−21.3, −10.5)		−20.7 (−28.6, −12.7)		−26.6 (−34.5, −18.8)	
WOMAC pain subscale	Control (n = 15)	−0.6 (−1.8, 0.7)	**0.0073**	−1.8 (−3.4, −0.1)	**0.0210**	−1.9 (−3.4, −0.4)	**0.0038**
	650 (n = 15)	−2.7 (−4.0, −1.3)		−5.2 (−7.0, −3.4)		−5.1 (−6.7, −3.5)	
	830 (n = 15)	−3.4 (−4.6, −2.1)		−4.2 (−5.9, −2.5)		−5.2 (−6.7, −3.7)	
WOMAC function subscale	Control (n = 15)	−4.4 (−8.6, −0.1)	0.0671	−6.5 (−12.3, −0.8)	0.0734	−6.3 (−12.2, −0.5)	**0.0055**
	650 (n = 15)	−6.6 (−11.3, −1.9)		−14.9 (−21.0, −8.3)		−17.1 (−23.6, −10.7)	
	830 (n = 15)	−11.3 (−15.6, −7.0)		−14.9 (−20.7, −9.1)		−19.4 (−25.3, −13.5)	
EQ-5D-5L	Control (n = 15)	0.04 (0.01, 0.08)	0.1667	0.06 (0.01, 0.11)	0.1568	0.03 (−0.02, 0.09)	**0.0131**
	650 (n = 15)	0.10 (0.05, 0.15)		0.10 (0.05, 0.15)		0.11 (0.05, 0.18)	
	830 (n = 15)	0.06 (0.02, 0.11)		0.13 (0.08, 0.18)		0.15 (0.10, 0.21)	

Values are expressed as least square adjusted means (95% confidence interval)

P-value for ANCOVA test adjusted baseline and BMI

WOMAC, Western Ontario and McMaster Universities Osteoarthritis Index; EQ-5D-5L, European Quality of Life Five Dimension Five Level Scale

The PGA (p = 0.0174, Visit 14 – Visit 7) was significantly different and rescue medication dosages were not significantly different among the three groups (Table 5). In addition, responder rate was not significantly different among the three groups (Table 6).

**Table 5:** Changes in the PGA and dosage of rescue medication (Visit13 vs. Visit7, Visit14 vs. Visit7) in the three groups.

Dependent Variable	Group	Difference (v13-v7)	P-value	Difference (v14-v7)	P-value
PGA	Control (n = 15)	−0.3 (−0.6, 0.0)	0.6695	0.0 (−0.3, 0.4)	**0.0174**
	650 (n = 15)	−0.5 (−0.8, −0.1)		−0.5 (−0.9, −0.2)	
	830 (n = 15)	−0.5 (−0.8, −0.2)		−0.7 (−1.0, −0.3)	
Dosage of rescue medication	Control (n = 15)	0.5 (−0.8, 1.9)	0.7904	1.7 (0.1, 3.3)	0.1042
	650 (n = 15)	0.3 (−1.1, 1.7)		−0.9 (−2.5, 0.8)	
	830 (n = 15)	1.0 (−0.4, 2.3)		0.4 (−1.1, 1.9)	

Values are expressed as least square adjusted means (95% confidence interval)

P-value for ANCOVA test adjusted baseline and BMI

PGA, patient’s global assessment

**Table 6:** Responder rate in one week after intervention completion.

	Control (n = 15)	650 (n = 15)	830 (n = 15)	χ²(p)
Responder	4 (26.67%)	8 (53.33%)	9 (60.00%)	0.1534
Non-responder	11 (73.33%)	7 (46.67%)	6 (40.00%)	

p-value for the among-group comparison using Chi–square test

Details of PGA and dosage of the rescue medication at Visits 7, 13, and 14 are provided in S2B Table.

A supplementary repeated-measures ANCOVA of efficacy outcomes is presented in S3 Table.

### Safety evaluation

Three AEs occurred during the study period. In the control group, one patient had general weakness and one had constipation. In the 830 group, one case of indigestion was reported. However, none of the AEs showed a causal relationship with the intervention and the patients recovered spontaneously. In addition, no ILA-related AEs were observed.

No significant abnormal changes were observed in either vital signs or laboratory test result except for the platelets (Visit 13 – screening visit, p = 0.0063) and creatinine (Visit 13 – screening Visit, p = 0.0178) levels. However, changes in the platelets and creatinine levels were within normal range.

Detailed changes in the vital signs and laboratory test results, as well as individual-level safety shifts, are shown in S4 Table

### Preliminary sample size estimation for future studies

Although we did not directly compare the superiority of the 830 nm and 650 nm wavelengths, we calculated the sample size for a larger future study focused on the 830 nm ILA based on the mean change observed in this group. Based on the differences in VAS score changes observed in the pilot trial, we conservatively set the mean difference (standard deviation) between the control and 830 nm groups at 12 (17) for the sample size estimation. With a 1:1 allocation ratio, 90% power, and a two-tailed significance level of 5%, 43 participants per group (86 in total) would be required. Considering the 15% dropout rate observed in the pilot trial, we estimated that 102 participants (51 per group) would need to be recruited.

## Discussion

We conducted the first RCT of ILA to investigate its safety and efficacy for KOA. This study provides a preliminary foundation for future research on this topic.

Our trial revealed several important findings. First, the 650 and 830 groups showed a significant reduction in VAS score at 1 week after intervention completion and until 6 weeks later. Second, the 650 and 830 groups showed a significant reduction in the WOMAC scores (total and pain and function subscales) 6 weeks after intervention completion. Third, a significant improvement in the EQ-5D-5L score at 6 weeks after intervention completion was observed in both the 650 and 830 groups. Fourth, no significant differences in the vital signs and laboratory test results were observed among the three groups, and no ILA-related AEs were reported.

The reasons behind the reduction in the VAS score and WOMAC total scores are likely attributed to the analgesic and anti-inflammatory effects of photobiomodulation (PBM). PBM exerts several effects on cells by therapeutically exposing cells to low levels of red or near-infrared light. First, in the intracellular compartment, PBM light is thought to modulate cytochrome c oxidase activity in mitochondria, thereby increasing adenosine triphosphate production and modulating intracellular levels of reactive oxygen species. Through this pathway, PBM has been reported to modulate the production of key pro-inflammatory mediators, including prostaglandin E2, interleukin-6, and tumor necrosis factor-alpha. Second, at the cell membrane, PBM may modulate ion channels such as transient receptor potential vanilloid 1, thereby attenuating nociceptor excitability and contributing to analgesia. Third, in the extracellular compartment, PBM may activate latent transforming growth factor-β1 through a redox-dependent activation mechanism, which promotes tissue repair and regeneration. Through these molecular mechanisms acting at multiple cellular compartments, PBM exerts analgesic, anti-inflammatory, and reparative effects, which may all contribute to the symptomatic improvement observed in patients with KOA [30–33].

In a KOA-induced rabbit model, LLL inhibited cartilage degeneration and promoted chondrocyte regeneration. Additionally, radiographic and three-dimensional computed tomography analyses revealed the restoration of bone density, articular surface, and joint surface and contour. These effects increased with prolongation of the treatment period [34]. Therefore, these changes are thought to be the reasons for pain and functional improvement in the ILA.

Effective delivery of energy to deeper structures—such as the synovium, articular cartilage, and muscle, which are pathologic sites in KOA—is important, as more than 90% of the energy from lasers used in musculoskeletal applications is absorbed within the first 10 mm of tissue [35]. Therefore, we adopted three key approaches. First, direct contact, which involves tightly pressing the light source onto the skin, is important for preventing dispersion and reflection [36]. ILA enables effective penetration beyond direct contact by allowing positioning of the acupuncture needle tip underneath the skin. Second, the power density, defined as the laser energy delivered per unit area (W/cm²), affects the depth of energy penetration [37]. High-level lasers are known to cause tissue damage, making it important to improve penetration without increasing the laser level [38]. By maintaining the same power output, the light source was positioned at the acupuncture needle tip to narrow the irradiated area. These methods effectively increased power density. Thus, our power density of 63.69 W/cm², which was higher than that employed in previous LA or LLL therapy trials [39], may have contributed to the observed significant results. Third, light wavelengths ranging from 650 to 900 nm were found to be optimal for penetrating the skin. Therefore, we adopted the wavelengths of 650 and 830 nm based on previous studies [14]. The depth of penetration depends not only on parameters, such as wavelength and power density, but also treatment technique, such as direct contact. These combined strategies may have contributed to the intergroup differences observed in multiple outcomes.

For the VAS outcomes, nominal between-group differences were not observed at Visit 7 but were observed at Visit 13 and were sustained at Visit 14. Although this time course might suggest a gradual response after repeated ILA sessions, the exploratory design and small sample size preclude any firm interpretation of this temporal pattern. The observation should therefore be regarded as a preliminary observation rather than evidence of a specific temporal effect.

Although no significant intergroup differences in the WOMAC function subscale and EQ-5D-5L scores were observed at Visits 7 and 13, substantial differences were observed at Visit 14. These findings may have been influenced by the common treatment in all groups, which included teaching of self-management and self-exercise—as strongly recommended in the ACR guidelines for KOA—during the intervention period [3].

The EQ-5D-5L findings in this trial should be interpreted cautiously. In this K-L grade 2–3 KOA population, baseline EQ-5D-5L scores were relatively high across the three groups (control, 0.74; 650 nm, 0.75; 830 nm, 0.73), leaving limited room for further improvement and raising the possibility of a ceiling effect. A previous study of patients with hip or knee OA established an MCID for improvement of 0.32 in surgically treated patients whose health improved, but among non-surgically managed patients the effect sizes were small and no clear minimal clinically important difference (MCID) exceeding the individual-level minimal detectable change could be established [40]. These findings suggest that EQ-5D-5L MCID estimates vary according to the patient population and treatment context. Accordingly, an established EQ-5D-5L MCID may not be directly applicable to a non-surgical, high-baseline population such as ours, which further limits the interpretation of the modest changes observed. Incorporating a complementary measure such as the EQ-VAS in future studies may help better characterize subtle patient-perceived changes in quality of life.

The responder rates were 26.67% in the control group, 53.33% in the 650 group, and 60% in the 830 group. Although the experimental groups showed numerically higher rates compared to the control group, the difference did not reach statistical significance. This lack of significance may be attributed to the small sample size. Minimal clinically important improvement (MCII), defined as the smallest change in measurement that signifies an important improvement in a patient’s symptom, for KOA has been established as −19.9 mm for pain VAS and −9.1 for the WOMAC function subscale [41]. In our study, the 650 group showed reductions of −20.7 mm in VAS at rest, −21.7 mm in VAS during activity, and −14.9 in the WOMAC function subscale. Similarly, the 830 group showed reductions of −22.6 mm in VAS at rest, −22.9 mm in VAS during activity, and −14.9 in the WOMAC function subscale. These improvements observed in both experimental groups exceeded the established MCII thresholds. Despite the lack of a statistically significant difference in responder rates, exceeding MCII thresholds suggests potential clinical relevance. In addition to MCII, MCID values for KOA have been reported as VAS 13.7 mm, WOMAC total 6.4, WOMAC pain 1.5, and WOMAC function 4.6 [42]. At the primary endpoint (Visit 13), the between group differences (vs. control) exceeded these MCID thresholds for most outcomes: VAS during activity (15.0 mm, 650 group; 16.2 mm, 830 group), WOMAC total (13.1, 650 group; 11.8, 830 group), WOMAC pain (3.4, 650 group; 2.4, 830 group), and WOMAC function (8.4 for both groups). For VAS at rest, the difference in the 830 group (15.4 mm) exceeded the MCID threshold, while the difference in the 650 group (13.5 mm) closely approached but marginally fell below the threshold. Considering the clinically meaningful differences across most outcomes, these findings also suggest potential clinical relevance. Nevertheless, further well-designed studies with larger sample sizes are necessary to substantiate these findings.

Despite these strengths, our study has several limitations. First, we conducted a single-center pilot study owing to the limitations of trial duration, research funding, and opportunities for recruitment. Therefore, the sample size and diversity were insufficient to fully validate the efficacy and safety of ILA in the treatment of KOA. These factors may have introduced bias into the trial results. In the future, multi-center trials with larger sample sizes should be conducted to encompass a more diverse patient population and enhance the external validity and clinical applicability of the results. Second, the follow-up duration in our study was relatively short. Consequently, this study was limited in assessing sustained efficacy, long-term safety, and potential AEs of ILA. To determine the long-term efficacy and safety of ILA, future studies should incorporate extended follow-up periods such as three to six months or longer. Third, owing to the characteristics of ILA, it was not possible to blind the practitioners, as they had to directly operate the laser device. To minimize this risk, we adopted a patient- and outcome assessor-blinded design. Nevertheless, because practitioners were aware of group allocation, their awareness and any associated intentions or expectations while administering the control treatment may have introduced performance bias across the treatment and control groups. The absence of practitioner blinding therefore remains a methodological limitation and may have influenced the trial results. In addition, although we implemented multiple measures to maintain participant and assessor blinding, the effectiveness of blinding was not evaluated using a validated blinding index. Therefore, we could not verify whether the blinding procedures were fully successful. Further research should explore strategies for blinding practitioners when possible, consider the inclusion of independent outcome assessments or more objective outcome measures, and assess blinding effectiveness to further mitigate potential bias. Fourth, various parameters are relevant to LLL (e.g., irradiation time, wavelength, intensity, frequency, wave type, peak power, and treatment interval). However, only the wavelength was changed and modifications to other parameters were limited in this trial. Thus, subsequent studies should involve varied parameter changes to determine the optimal parameters. Fifth, LLL therapy application is influenced by a complex combination of parameters, delivery techniques, and irradiation sites [38]. As this exploratory pilot trial was primarily designed to investigate the wavelength-dependent effects of ILA, we prioritized comparing the 650 and 830 nm groups with a sham laser control. Consequently, our study did not incorporate a non-acupoint sham control, limiting our ability to determine whether the observed improvements were attributable to specific acupoints or to non-specific deep-tissue stimulation. Future studies incorporating sham acupoint designs are warranted to clarify the role of site specificity. Sixth, missing data were managed using the LOCF method, which is considered suboptimal compared to more robust approaches, such as multiple imputation or mixed-effects modeling [43]. Future studies using more advanced statistical methods are needed to better establish the efficacy of ILA on KOA. Seventh, this exploratory pilot trial measured multiple outcomes at several time points, and the individual visit-wise p-values in the main ANCOVA analyses were not adjusted for multiple temporal comparisons, raising the possibility of an inflated type I error. To address this limitation, we additionally performed a supplementary repeated measures ANCOVA to evaluate the group-by-visit interaction across visits. The results supported longitudinal group differences for the primary VAS outcomes, whereas most secondary outcomes were less robust. As this analysis was not prespecified in the protocol and the study had a limited sample size, these supplementary findings should be regarded as exploratory. More broadly, the statistically significant findings of the main analyses should therefore be interpreted as preliminary and hypothesis-generating rather than confirmatory, and future adequately powered confirmatory trials with prespecified longitudinal analyses and multiplicity-control strategies are warranted. Eighth, we did not restrict enrollment to patients with unilateral KOA to reflect real-world clinical practice, while treatment was confined to a single predefined target knee and VAS/WOMAC assessments were conducted for the predefined target knee. However, symptoms in the contralateral knee were not monitored over time. Previous evidence indicates that patient-reported pain and physical function in KOA may be influenced by contralateral knee pain and by unilateral versus bilateral symptom status [44,45]. We therefore could not determine whether the relative severity between the two knees shifted during follow-up, and an influence of the untreated contralateral knee on the patient-reported outcomes cannot be excluded. Prior work also suggests that bilateral involvement may matter clinically: between-limb gait asymmetry has been reported to be more common in bilateral than in unilateral mild-to-moderate KOA [46], and coexisting contralateral KOA has been linked to faster structural progression and a higher subsequent arthroplasty rate in the index knee [47]. Because the symptomatic unilateral/bilateral KOA status was not recorded as a study variable, and given the small per-group sample of this exploratory pilot trial, a laterality-based subgroup or sensitivity analysis was not feasible. Future confirmatory trials should prospectively document unilateral versus bilateral involvement, collect outcomes for both knees, and consider stratified or sensitivity analyses based on laterality.

## Conclusions

In this exploratory pilot trial, ILA at 650 and 830 nm showed preliminary signals of short-term pain reduction among patients with KOA. However, because this was a pilot study with a small sample size and exploratory design, adequately powered, confirmatory trials are warranted to validate these preliminary observations.

## Supporting information

S1 Fig Medical device for low-level laser combined with acupuncture; Ellise. (TIF)

S1 Table Revised Standards for Reporting Intervention in Clinical Trials of Acupuncture (STRICTA). (DOCX)

S2 Table Efficacy outcomes across time points. *S2A Table*. VAS, WOMAC and EQ-5D-5L scores at Visits 1, 7, 13, and 14. *S2B Table*. PGA and Dosage of rescue medication at Visit 7, Visit 13, and Visit 14.
(DOCX)

S3 Table Supplementary repeated-measures ANCOVA of longitudinal outcomes. (DOCX)

S4 Table Safety Outcomes. *S4A Table*. Changes in vital signs among the control, 650, and 830 groups. *S4B Table*. Changes in laboratory test among the control, 650, and 830 groups. *S4C Table*. Descriptive summary of individual-level safety shifts in laboratory parameters and vital signs, by treatment group.
(DOCX)

S1 File CONSORT 2025 checklist. (DOCX)

S2 File Data. (DOCX)

S3 File Study protocol English and Korean version. (PDF)
