# Supplementary material for: Efficacy and safety of invasive laser acupuncture (650 and 830 nm) on knee osteoarthritis: A pilot randomized clinical trial
Source: PLoS One. 2026 Jul 20;21(7):e0353654. doi: 10.1371/journal.pone.0353654 (PMC13384278; doi:10.1371/journal.pone.0353654)
Supplement: S1 Table — (DOCX) [file pone.0353654.s002.docx]

**S1 Table. Revised Standards for Reporting Intervention in Clinical Trials of Acupuncture (STRICTA)**

|  | **Item Criteria** | **Description** |
| --- | --- | --- |
| 1.Acupuncture rationale | 1a) Style of acupuncture | Laser acupuncture |
|  | 1b) Reasoning for treatment provided – based on historical context, literature sources, and/or consensus methods, with references where appropriate | 1) Discussion among three doctors that practice Korean medicine (consensus)  2) Selection of treatment regions based on related papers and expert discussions [20,21] |
|  | 1c) Extent to which treatment varied | Standardized treatment |
| 2. Details of needling | 2a) Number of needle insertions per subject per session (mean and range where relevant) | 7 |
|  | 2b) Names (or location if no standard name) of points used (uni-/bilateral) | EX-LE4 (Neixiyan), ST35 (Dubi), ST34 (Liangqiu), SP10 (Xuehai), SP9 (Yinglingquan), GB34 (Yanglingquan), and EX-LE2 (Heding) on the lesion side (the more painful side if bilateral) |
|  | 2c) Depth of insertion, based on a specified unit of measurement or on a particular tissue level | Acupuncture needle was inserted vertically or obliquely at a depth of 1–3 cm, depending the location of the needle. |
|  | 2d) Responses sought | No de qi or muscle twitching,– only sensation due to needle insertion |
|  | 2e) Needle stimulation | The real laser parameters will be 20 mW power, pulse type wave, 12J/point energy dose, 50 Hz frequency, and 63.69 W/cm^2^ power density. |
|  | 2f) Needle retention time | 10-minute per session |
|  | 2g) Needle type | disposable acupuncture needle with optical fibers inserted inside |
| 3. Treatment regimen | 3a) Number of treatment sessions | 12 |
|  | 3b) Frequency and duration of treatment sessions | twice/week for 6weeks, 10-min per session |
| 4. Other treatment components | 4a) Details of other interventions administered to the acupuncture group | education on exercise and self-care during their treatment visits |
|  | 4b) Setting and context of treatment – including instructions to practitioners – as well as information and explanations given to patients | Practitioners who will conduct the intervention will be trained to ensure adherence to the intervention protocol. All patients will be educated on exercise and self-care during their treatment visits. |
| 5. Practitioner background | 5a) Description of participating acupuncturists | Korean medicine doctor with the following qualifications: 6 years of formal university training in Korean medicine, a license |
| 6. Control or comparator interventions | 6a) Rationale for the control or comparator in the context of the research question, with sources that justify the choice | Relevant articles [20,21] |
|  | 6b) Precise description of the control or comparator; details for items 1−3 above with the use of sham acupuncture or any other type of acupuncture-like control | In supine position with participant’s knees bent, the acupuncture needle will be inserted in EX-LE4 (Neixiyan), ST35 (Dubi), ST34 (Liangqiu), SP10 (Xuehai), SP9 (Yinglingquan), GB34 (Yanglingquan), and EX-LE2 (Heding) on the lesion side (the more painful side if bilateral) and then the laser output device will be turned on for 10 min (830 group, 20mW power; 650 group, 20mW power; control group, 0mW power). During the procedures, participants will be blindfolded, and no differences in sounds or feelings will be found among the three groups |
