# Supplementary material for: Efficacy and safety of invasive laser acupuncture (650 and 830 nm) on knee osteoarthritis: A pilot randomized clinical trial
Source: PLoS One. 2026 Jul 20;21(7):e0353654. doi: 10.1371/journal.pone.0353654 (PMC13384278; doi:10.1371/journal.pone.0353654)
Supplement: S2 Table — S2A Table. VAS, WOMAC and EQ-5D-5L scores at Visits 1, 7, 13, and 14. S2B Table. PGA and Dosage of rescue medication at Visit 7, Visit 13, and Visit 14. (DOCX) [file pone.0353654.s003.docx]

**S2A Table. VAS, WOMAC and EQ-5D-5L scores at Visits 1, 7, 13, and 14**

| Dependent Variable | Group | Visit 1  (Baseline) | Visit 7 | Visit 13 | Visit 14 |
| --- | --- | --- | --- | --- | --- |
| At rest VAS | Control (n=15) | 47.5 (8.9)  49.0 (40.0, 50.0) | 42.8 (10.6)  41.0 (37.0, 50.0) | 40.8 (13.5)  40.0 (30.0, 50.0) | 40.8 (16.3)  40.0 (31.0, 51.0) |
|  | 650 (n=15) | 48.5 (11.4)  49.0 (39.0, 50.0) | 38.5 (8.4)  39.0 (31.0, 47.0) | 27.1 (14.2)  30.0 (13.0, 39.0) | 25.3 (11.4)  29.0 (19.0, 30.0) |
|  | 830 (n=15) | 48.3 (10.4)  49.0 (40.0, 50.0) | 36.1 (10.5)  36.0 (30.0, 48.0) | 25.8 (12.1)  25.0 (20.0, 37.0) | 19.9 (12.9)  19.0 (10.0, 26.0) |
| Activity VAS | Control (n=15) | 49.9 (6.4)  50.0 (45.0, 56.0) | 45.9 (13.8)  50.0 (38.0, 54.0) | 43.6 (15.2)  46.0 (38.0, 51.0) | 44.3 (16.0)  45.0 (40.0, 56.0) |
|  | 650 (n=15) | 50.4 (14.2)  49.0 (40.0, 51.0) | 38.5 (16.2)  37.0 (29.0, 46.0) | 28.2 (12.4)  31.0 (17.0, 36.0) | 28.7 (12.8)  30.0 (22.0, 40.0) |
|  | 830 (n=15) | 50.9 (11.6)  46.0 (40.0, 59.0) | 37.4 (16.4)  33.0 (25.0, 50.0) | 28.1 (14.7)  23.0 (20.0, 39.0) | 19.0 (16.4)  10.0 (9.0, 32.0) |
| WOMAC total | Control (n=15) | 67.8 (18.2)  64.0 (60.0, 83.0) | 62.2 (17.6)  65.0 (40.0, 78.0) | 57.0 (19.3)  61.0 (39.0, 74.0) | 57.3 (20.5)  59.0 (38.0, 78.0) |
|  | 650 (n=15) | 61.7 (13.7)  64.0 (52.0, 68.0) | 48.8 (9.9)  50.0 (43.0, 54.0) | 40.6 (11.5)  39.0 (33.0,48.0) | 36.9 (12.4)  30.0 (27.0, 46.0) |
|  | 830 (n=15) | 63.2 (11.5)  64.0 (60.0, 71.0) | 49.5 (12.5)  51.0 (37.0, 61.0) | 43.4 (14.6)  37.0 (30.0, 55.0) | 37.9 (13.8)  32.0 (28.0, 48.0) |
| WOMAC pain subscale | Control (n=15) | 13.7 (3.9)  15.0 (12.0, 15.0) | 12.9 (3.0)  14.0 (10.0, 15.0) | 11.3 (4.0)  13.0 (6.0, 15.0) | 11.5 (4.0)  12.0 (9.0, 15.0) |
|  | 650 (n=15) | 12.8 (3.2)  13.0 (10.0, 14.0) | 9.5 (2.7)  9.0 (7.0, 12.0) | 7.9 (2.4)  7.0 (6.0, 9.0) | 7.5 (2.7)  6.0 (5.0, 9.0) |
|  | 830 (n=15) | 12.3 (2.9)  13.0 (11.0, 15.0) | 9.7 (2.4)  10.0 (7.0, 11.0) | 8.5 (2.7)  8.0 (6.0, 11.0) | 7.6 (2.6)  6.0 (3.0, 7.0) |
| WOMAC function subscale | Control (n=15) | 48.5 (13.7)  47.0 (42.0, 60.0) | 44.2 (13.6)  47.0 (29.0, 56.0) | 40.9 (13.9)  43.0 (27.0, 52.0) | 41.3 (15.0)  43.0 (26.0, 56.0) |
|  | 650 (n=15) | 43.6 (10.1)  44.0 (35.0, 48.0) | 35.5 (7.8)  36.0 (32.0, 42.0) | 29.5 (8.8)  27.0 (24.0, 35.0) | 26.6 (9.3)  22.0 (19.0, 35.0) |
|  | 830 (n=15) | 45.7 (8.0)  46.0 (42.0, 51.0) | 35.9 (10.0)  34.0 (27.0, 43.0) | 31.4 (11.2)  25.0 (22.0, 40.0) | 27.1 (10.4)  24.0 (20.0, 32.0) |
| EQ-5D-5L | Control (n=15) | 0.74 (0.66, 0.80) | 0.76 (0.68, 0.80) | 0.76 (0.74, 0.82) | 0.76 (0.70, 0.80) |
|  | 650 (n=15) | 0.75 (0.68, 0.80) | 0.84 (0.73, 0.86) | 0.80 (0.76, 0.86) | 0.81 (0.78, 0.86) |
|  | 830 (n=15) | 0.73 (0.68, 0.75) | 0.76 (0.73, 0.80) | 0.83 (0.76, 0.86) | 0.85 (0.76, 1.00) |

Values are expressed as means (standard deviation), medians (Q1, Q3)

VAS, visual analog scale; WOMAC, Western Ontario and McMaster Universities Osteoarthritis Index; EQ-5D-5L, European Quality of Life Five Dimension Five Level Scale; Q1, first quartiles; Q3, third quartiles.

**S2B Table.** **PGA and Dosage of rescue medication at Visits 7, 13, and 14**

| Dependent Variable | Group | Visit 7 | Visit 13 | Visit 14 |
| --- | --- | --- | --- | --- |
|  |  | m(Q1Q3) | m(Q1,Q3) | m(Q1,Q3) |
| PGA | Control (n=15) | 4.0 (3.0, 4.0) | 4.0 (4.0, 4.0) | 4.0 (3.0, 4.0) |
|  | 650 (n=15) | 4.0 (3.0, 4.0) | 4.0 (4.0, 5.0) | 4.0 (4.0, 5.0) |
|  | 830 (n=15) | 4.0 (4.0, 4.0) | 4.0 (4.0, 5.0) | 4.5 (4.0, 5.0) |
| Dosage of rescue medication | Control (n=15) | 0.0 (-2.5, 0.0) | 0.0 (0.0, 1.5) | 0.0 (0.0, 0.5) |
|  | 650 (n=15) | 0.0 (-2.0, 0.0) | 0.0 (0.0, 2.0) | 0.0 (0.0, 4.0) |
|  | 830 (n=15) | 0.0 (-4.0, 0.0) | 0.0 (0.0, 1.0) | 0.0 (0.0, 2.0) |

Values are expressed as medians (Q1, Q3)

PGA, patient's global assessment; Q1, first quartiles; Q3: third quartiles.
