# Supplementary material for: Efficacy and safety of invasive laser acupuncture (650 and 830 nm) on knee osteoarthritis: A pilot randomized clinical trial
Source: PLoS One. 2026 Jul 20;21(7):e0353654. doi: 10.1371/journal.pone.0353654 (PMC13384278; doi:10.1371/journal.pone.0353654)
Supplement: S3 Table — (DOCX) [file pone.0353654.s004.docx]

**S3 Table. Supplementary repeated-measures ANCOVA of longitudinal outcomes**

| Dependent Variables | Source | F value | P-value |  |
| --- | --- | --- | --- | --- |
| VAS at rest | Group | 5.77 | 0.0062 |  |
|  | Visit (week) | 0.48 | 0.6975 |  |
|  | Visit (week)×Group | 5.07 | **0.0001** |  |
| VAS during activity | Group | 5.52 | 0.0075 |  |
|  | Visit (week) | 0.36 | 0.7853 |  |
|  | Visit (week)×Group | 4.93 | **0.0001** |  |
| WOMAC total | Group | 6.06 | 0.0049 |  |
|  | Visit (week) | 1.48 | 0.2246 |  |
|  | Visit (week)×Group | 1.98 | 0.0733 |  |
| WOMAC  pain subscale | Group | 7.66 | 0.0015 |  |
|  | Visit (week) | 2.51 | 0.0615 |  |
|  | Visit (week)×Group | 1.38 | 0.2260 |  |
| WOMAC  Function subscale | | Group | 5.21 | 0.0097 |
|  |  | Visit (week) | 1.20 | 0.3122 |
|  |  | Visit (week)×Group | 2.10 | 0.0574 |
| EQ-5D-5L | Group | 1.92 | 0.1593 |  |
|  | Visit (week) | 0.13 | 0.9399 |  |
|  | Visit (week)×Group | 3.05 | **0.0082** |  |
| PGA | Group | 4.96 | 0.0123 |  |
|  | Visit (week) | 0.07 | 0.9281 |  |
|  | Visit (week)×Group | 1.40 | 0.2437 |  |

As a supplementary post hoc analysis, repeated-measures ANCOVA was performed to evaluate visit × group interaction effects across post-baseline visits.
