# Supplementary material for: Efficacy and safety of invasive laser acupuncture (650 and 830 nm) on knee osteoarthritis: A pilot randomized clinical trial
Source: PLoS One. 2026 Jul 20;21(7):e0353654. doi: 10.1371/journal.pone.0353654 (PMC13384278; doi:10.1371/journal.pone.0353654)
Supplement: S4 Table — S4A Table. Changes in vital signs among the control, 650, and 830 groups. S4B Table. Changes in laboratory test among the control, 650, and 830 groups. S4C Table. Descriptive summary of individual-level safety shifts in laboratory parameters and vital signs, by treatment group. (DOCX) [file pone.0353654.s005.docx]

**S4A Table. Changes in vital signs among the control, 650, and 830 groups**

|  | Control (n=15) | 650 (n=15) | 830 (n=15) | P-value |
| --- | --- | --- | --- | --- |
| Systolic blood pressure (mmHg) | | | | |
| Visit 1 (Baseline) | 125.2 (12.0) | 128.3 (12.0) | 127.9 (7.7) |  |
| Visit 13 | 125.3 (8.3) | 123.5 (13.5) | 125.5 (9.7) |  |
| Difference  (v13 – v1) | 0.3 (12.0) | -3.8 (11.1) | -3.1 (8.2) | 0.5899^†^ |
| Visit 14 | 125.0 (9.8) | 130.1 (10.9) | 122.8 (12.2) |  |
| Difference  (v14 - v1) | -0.1 (13.8) | 2.8 (10.2) | -5.8 (12.9) | 0.1859^†^ |
| Diastolic blood pressure (mmHg) | | | | |
| Visit 1 (Baseline) | 79.3 (8.8) | 80.3 (8.6) | 78.8 (8.6) |  |
| Visit 13 | 79.2 (9.8) | 79.1 (9.9) | 79.7 (7.5) |  |
| Difference  (v13 – v1) | -0.3 (8.4) | -1.4 (7.5) | -0.6 (6.3) | 0.9270^†^ |
| Visit 14 | 80.1 (8.2) | 82.7 (6.3) | 79.1 (7.0) |  |
| Difference  (v14 - v1) | 0.7 (7.2) | 2.2 (5.3) | -1.2 (7.6) | 0.4126^†^ |
| Pulse rate (per minute) | | | | |
| Visit 1 (Baseline) | 71.3 (13.5) | 67.7 (10.6) | 76.3 (9.5) |  |
| Visit 13 | 69.6 (12.1) | 70.1 (10.8) | 75.4 (8.5) |  |
| Difference  (v13 – v1) | 0.6 (7.5) | 2.8 (7.0) | -1.9 (10.6) | 0.3676^†^ |
| Visit 14 | 67.1 (10.1) | 70.9 (11.9) | 75.6 (6.4) |  |
| Difference  (v14 - v1) | -1.9 (9.0) | 3.6 (9.6) | -1.6 (9.2) | 0.2270^†^ |
| Body temperature (℃) | | | | |
| Visit 1 (Baseline) | 36.4 (0.4)  36.5 (36.0, 36.7) | 36.7 (0.4)  36.7 (36.4, 37.0) | 36.3 (0.3)  36.7 (36.3, 36.8) |  |
| Visit 13 | 36.4 (0.4)  36.3 (36.1, 36.8) | 36.5 (0.3)  36.6 (36.4, 36.7) | 36.5 (0.3)  36.5 (36.2, 36.7) |  |
| Difference  (v13 – v1) | 0 (0.3) | -0.1 (0.4) | -0.1 (0.5) | 0.5108^†^ |
| Visit 14 | 36.3 (0.3)  36.2 (36.0, 36.6) | 36.3 (0.4)  36.2 (36.0, 36.7) | 36.4 (0.3)  36.5 (36.1, 36.7) |  |
| Difference  (v14 - v1) | -0.4 (-0.5, 0.5) | -0.4 (-0.6, 0.0) | -0.1 (-0.5, 0.0) | 0.5624^#^ |

Values are expressed as means (standard deviation), medians (Q1, Q3)

†: p-value for ANOVA test, #: p-value for Kruskal-Wallis test

Q1, first quartiles; Q3, third quartiles.

**S4B Table. Changes in laboratory test among the control, 650, and 830 groups**

| Dependent Variable | Group | Screening visit (visit 0) | Visit 13 | Difference  (v13 – v0) | P-value |
| --- | --- | --- | --- | --- | --- |
| White blood count (10³/μL) | Control (n=15) | 6.1 (5.1, 7.1) | 5.6 (5.2, 6.3) | -0.4 (-1.1, 0) | 0.2382^#^ |
|  | 650 (n=15) | 5.6 (4.5, 6.6) | 5.5 (4.9, 6.1) | 0.2 (-0.9, 1.8) |  |
|  | 830 (n=15) | 5.7 (4.1, 6.9) | 4.8 (4.5, 6.2) | -0.4 (-1, 0) |  |
| Red blood count (10⁶/μL) | Control (n=15) | 4.2 (3.8, 4.4) | 4 (3.8, 4.3) | -0.1 (-0.3, 0) | 0.3088^#^ |
|  | 650 (n=15) | 4.1 (4, 4.4) | 4.2 (4, 4.3) | 0 (-0.1, 0.2) |  |
|  | 830 (n=15) | 4.1 (3.8, 4.5) | 4.2 (3.8, 4.3) | 0 (-0.1, 0.1) |  |
| Hemoglobin (g/dl) | Control (n=15) | 13.1 (0.9) | 12.7 (1.3) | -0.4 (0.8) | 0.4640^†^ |
|  | 650 (n=15) | 13.2 (0.8) | 13.1 (1) | -0.1 (0.6) |  |
|  | 830 (n=15) | 13.3 (0.8) | 13.2 (0.9) | -0.1 (0.4) |  |
| Hematocrit (%) | Control (n=15) | 40.0 (2.5) | 39.1 (3.7) | -0.9 (2.5) | 0.5795^†^ |
|  | 650 (n=15) | 40.4 (2.4) | 40.1 (2.8) | -0.2 (1.8) |  |
|  | 830 (n=15) | 40.5 (2.5) | 40.4 (2.6) | -0.2 (1.2) |  |
| Platelet (10³/μL) | Control (n=15) | 214.5 (41.5) | 192.9 (28) | -16.3 (21.1) | **0.0063^†^** |
|  | 650 (n=15) | 193.8 (34.6) | 210.5 (49) | 16.1 (31.5) |  |
|  | 830 (n=15) | 194.4 (47.4) | 186.6 (42.7) | -9.4 (23.3) |  |
| Glucose (mg/dl) | Control (n=15) | 123.1 (37.4) | 105.8 (42.8) | -17.9 (21.7) | 0.1855^†^ |
|  | 650 (n=15) | 123.1 (18.5) | 122.9 (19.1) | -0.2 (21.4) |  |
|  | 830 (n=15) | 126.2 (34.1) | 122.9 (27.5) | -1.7 (33.2) |  |
| BUN (mg/dl) | Control (n=15) | 19.2 (3.9) | 19.5 (4) | -0.1 (4.9) | 0.4138^†^ |
|  | 650 (n=15) | 15.2 (2.8) | 17.3 (5.3) | 2.1 (4.9) |  |
|  | 830 (n=15) | 16.2 (4.9) | 16 (3.1) | -0.1 (5.3) |  |
| Creatinine (mg/dl) | Control (n=15) | 0.7 (0.6, 0.8) | 0.8 (0.7, 0.8) | 0 (0, 0.1) | **0.0178^#^** |
|  | 650 (n=15) | 0.6 (0.6, 0.7) | 0.7 (0.6, 0.8) | 0.1 (0, 0.2) |  |
|  | 830 (n=15) | 0.7 (0.6, 0.7) | 0.6 (0.6, 0.7) | 0 (-0.1, 0) |  |
| Na (mmol/L) | Control (n=15) | 142 (141, 143) | 142.5 (140.5, 144) | 0 (-1, 1) | 0.9095^#^ |
|  | 650 (n=15) | 144 (143, 145) | 144 (143, 145) | -0.5 (-1,1) |  |
|  | 830 (n=15) | 143 (143, 144) | 143 (142, 143) | 0 (-1, 1) |  |
| K (mmol/L) | Control (n=15) | 4.3 (0.3) | 4.3 (0.3) | 0 (0.4) | 0.9868^†^ |
|  | 650 (n=15) | 4.2 (0.5) | 4.2 (0.4) | 0 (0.3) |  |
|  | 830 (n=15) | 4.3 (0.4) | 4.3 (0.3) | 0 (0.4) |  |
| Cl (mmol/L) | Control (n=15) | 105 (103, 106) | 105 (104.5, 106) | 0 (-0.5, 2) | 0.7989^#^ |
|  | 650 (n=15) | 106 (105, 107) | 106 (105, 107) | 0 (-1, 1) |  |
|  | 830 (n=15) | 106 (104, 107) | 106 (105, 106) | 0.5 (-1, 1) |  |
| Total protein (g/dl) | Control (n=15) | 7.6 (0.2) | 7.4 (0.3) | -0.3 (0.4) | 0.0796^†^ |
|  | 650 (n=15) | 7.5 (0.3) | 7.5 (0.3) | 0 (0.3) |  |
|  | 830 (n=15) | 7.3 (0.5) | 7.1 (0.4) | -0.1 (0.3) |  |
| Albumin (g/dl) | Control (n=15) | 4.3 (0.2) | 4.4 (0.2) | 0.1 (0.2) | 0.4259^†^ |
|  | 650 (n=15) | 4.3 (0.3) | 4.5 (0.4) | 0.2 (0.1) |  |
|  | 830 (n=15) | 4.3 (0.2) | 4.4 (0.4) | 0 (0.3) |  |
| ALP (U/L) | Control (n=15) | 85.0 (62.0, 102.0) | 81 (62, 100) | -2.5 (-15, -0.5) | 0.4688^#^ |
|  | 650 (n=15) | 89 (69, 103) | 76.5 (60, 90) | -2 (-19, 5) |  |
|  | 830 (n=15) | 81 (68, 93) | 74.5 (61, 88) | 0 (-10, 7) |  |
| AST (U/L) | Control (n=15) | 21.3 (6.0) | 21.8 (5) | 0.3 (4.8) | 0.3738^†^ |
|  | 650 (n=15) | 21.2 (3.2) | 22.2 (3.1) | 1.1 (3.4) |  |
|  | 830 (n=15) | 21.3 (4.8) | 24 (6.9) | 2.8 (5.2) |  |
| ALT (U/L) | Control (n=15) | 19.9 (7) | 18.2 (4.2) | -1.5 (6) | 0.4603^†^ |
|  | 650 (n=15) | 20.3 (7.7) | 20.6 (4.1) | 0.4 (7.7) |  |
|  | 830 (n=15) | 20.1 (6.1) | 22 (7.6) | 2.1 (8) |  |
| GGT (U/L) | Control (n=15) | 20 (15, 23) | 17 (12, 20) | -2 (-3, 0) | 0.0942^#^ |
|  | 650 (n=15) | 19 (17, 22) | 19 (17, 26) | 0.5 (-1, 5) |  |
|  | 830 (n=15) | 15 (11, 22) | 16.5 (14, 26) | 0.5 (-3, 2) |  |
| Total bilirubin (mg/dl) | Control (n=15) | 0.8 (0.7, 1) | 0.9 (0.6, 0.9) | 0 (-0.2, 0.1) | 0.8599^#^ |
|  | 650 (n=15) | 0.8 (0.7, 0.9) | 0.7 (0.6, 1) | -0.1 (-0.2, 0.1) |  |
|  | 830 (n=15) | 0.8 (0.7, 1) | 0.8 (0.7, 1.1) | -0.1 (-0.2, -0.1) |  |

Values are expressed as means (standard deviation), medians (Q1, Q3)

†, p-value for ANOVA test; #, p-value for Kruskal-Wallis test for between group.

BUN, blood urea nitrogen; ALP, alkaline phosphatase; AST, aspartate aminotransferase; ALT, alanine aminotransferase; GGT, gamma-glutamyl transferase.

**S4C Table. Descriptive summary of individual-level safety shifts in laboratory parameters and vital signs, by treatment group**

|  | Control (n=15) | 650 (n=15) | 830 (n=15) |
| --- | --- | --- | --- |
| **Summary of clinically significant safety findings** | | | |
| Any potentially clinically significant vital-sign shift | 0 (0.0) | 0 (0.0) | 0 (0.0) |
| Any clinically significant laboratory abnormality | 0 (0.0) | 0 (0.0) | 0 (0.0) |
| Any laboratory abnormality reported as an adverse event | 0 (0.0) | 0 (0.0) | 0 (0.0) |
| Any laboratory abnormality considered related to intervention | 0 (0.0) | 0 (0.0) | 0 (0.0) |
| Any safety abnormality requiring additional medical intervention or treatment discontinuation | 0 (0.0) | 0 (0.0) | 0 (0.0) |
| **Detailed potentially clinically significant vital-sign shifts** | | | |
| Systolic blood pressure | 0 (0.0) | 0 (0.0) | 0 (0.0) |
| Diastolic blood pressure | 0 (0.0) | 0 (0.0) | 0 (0.0) |
| Pulse rate | 0 (0.0) | 0 (0.0) | 0 (0.0) |
| Body temperature | 0 (0.0) | 0 (0.0) | 0 (0.0) |
| **Detailed laboratory out-of-range shifts** | | | |
| White blood count | 1 (6.7) | 1 (6.7) | 2 (13.3) |
| Red blood count | 1 (6.7) | 3 (20.0) | 1 (6.7) |
| Hemoglobin | 1 (6.7) | 1 (6.7) | 1 (6.7) |
| Hematocrit | 1 (6.7) | 0 (0.0) | 1 (6.7) |
| Platelet | 0 (0.0) | 1 (6.7) | 1 (6.7) |
| Glucose | 1 (6.7) | 2 (13.3) | 3 (20.0) |
| BUN | 1 (6.7) | 2 (13.3) | 0 (0.0) |
| Creatinine | 0 (0.0) | 0 (0.0) | 0 (0.0) |
| Na | 0 (0.0) | 2 (13.3) | 0 (0.0) |
| K | 0 (0.0) | 0 (0.0) | 0 (0.0) |
| Cl | 0 (0.0) | 0 (0.0) | 0 (0.0) |
| Total protein | 0 (0.0) | 0 (0.0) | 0 (0.0) |
| Albumin | 0 (0.0) | 0 (0.0) | 0 (0.0) |
| ALP | 0 (0.0) | 0 (0.0) | 0 (0.0) |
| AST | 0 (0.0) | 1 (6.7) | 0 (0.0) |
| ALT | 0 (0.0) | 0 (0.0) | 0 (0.0) |
| GGT | 0 (0.0) | 0 (0.0) | 0 (0.0) |
| Total bilirubin | 0 (0.0) | 1 (6.7) | 0 (0.0) |

Values are the number (%) of participants with treatment-emergent shifts

Laboratory out-of-range shifts were defined as values within the reference interval at screening visit that shifted outside the reference interval at the visit 13 laboratory assessment. laboratory out-of-range shifts were summarized descriptively and were not necessarily considered clinically significant abnormalities.

BUN, blood urea nitrogen; ALP, alkaline phosphatase; AST, aspartate aminotransferase; ALT, alanine aminotransferase; GGT, gamma-glutamyl transferase.
