# Supplementary material for: Efficacy and safety of invasive laser acupuncture (650 and 830 nm) on knee osteoarthritis: A pilot randomized clinical trial
Source: PLoS One. 2026 Jul 20;21(7):e0353654. doi: 10.1371/journal.pone.0353654 (PMC13384278; doi:10.1371/journal.pone.0353654)
Supplement: S2 File — (DOCX) [file pone.0353654.s007.docx]

**Raw data about efficacy outcomes**

VAS score at rest

|  | Group | VAS at rest at visit 1 | VAS at rest at visit 7 | VAS at rest at visit 13 | VAS at rest at visit 14 |
| --- | --- | --- | --- | --- | --- |
| DS-R-001 | 830 | 58 | 40 | 37 | 35 |
| DS-R-002 | 830 | 33 | 19 | 8 | 0 |
| DS-R-003 | 650 | 35 | 22 | 10 | 6 |
| DS-R-004 | Control | 49 | 41 | 40 | 40 |
| DS-R-005 | Control | 49 | 19 | 9 | 60 |
| DS-R-006 | 650 | 65 | 52 | 39 | 37 |
| DS-R-007 | 830 | 50 |  |  |  |
| DS-R-008 | 650 | 40 | 38 | 37 | 10 |
| DS-R-009 | 650 | 49 | 47 | 44 | 25 |
| DS-R-010 | Control | 39 | 39 | 39 | 25 |
| DS-R-011 | Control | 37 | 37 | 36 | 38 |
| DS-R-012 | Control | 49 | 40 | 39 | 40 |
| DS-R-013 | Control | 46 |  |  |  |
| DS-R-014 | 650 | 42 | 39 | 13 | 9 |
| DS-R-015 | 650 | 79 | 48 | 9 | 19 |
| DS-R-016 | 830 | 45 | 30 | 25 | 19 |
| DS-R-017 | 830 | 50 | 29 | 20 | 20 |
| DS-R-018 | 830 | 40 | 20 | 20 | 10 |
| DS-R-019 | Control | 49 | 40 | 40 | 29 |
| DS-R-020 | 830 | 51 |  |  |  |
| DS-R-021 | 830 | 50 | 50 |  |  |
| DS-R-022 | Control | 37 | 30 | 25 | 25 |
| DS-R-023 | 650 | 50 | 30 | 30 | 29 |
| DS-R-024 | Control | 36 | 31 | 28 | 31 |
| DS-R-025 | 830 | 75 | 51 | 40 | 25 |
| DS-R-026 | 650 | 51 | 30 | 30 | 29 |
| DS-R-027 | 650 | 49 | 31 | 0 | 32 |
| DS-R-028 | 650 | 50 |  |  |  |
| DS-R-029 | 830 | 40 | 30 | 28 | 19 |
| DS-R-030 | 650 | 70 | 40 | 50 | 51 |
| DS-R-031 | Control | 49 | 48 | 10 | 10 |
| DS-R-032 | 830 | 41 | 45 | 30 | 29 |
| DS-R-033 | Control | 49 | 40 | 29 | 10 |
| DS-R-034 | Control | 59 | 60 | 60 | 60 |
| DS-R-035 | 830 | 39 | 35 | 18 | 19 |
| DS-R-036 | 650 | 48 | 30 | 24 | 26 |
| DS-R-037 | 650 | 35 | 35 | 30 | 30 |
| DS-R-038 | 830 | 60 | 50 | 30 | 10 |
| DS-R-039 | Control | 50 | 50 | 49 | 40 |
| DS-R-040 | 830 | 50 | 39 | 35 | 29 |
| DS-R-041 | Control | 38 | 35 | 28 | 7 |
| DS-R-042 | 650 | 41 | 36 | 10 | 2 |
| DS-R-043 | 650 | 45 | 40 | 30 | 30 |
| DS-R-044 | Control | 47 | 59 | 59 | 59 |
| DS-R-045 | 830 | 49 | 39 | 20 | 19 |

VAS score during activity

|  | Group | VAS during activity at visit 1 | VAS during activity at visit 7 | VAS during activity at visit 13 | VAS during activity at visit 14 |
| --- | --- | --- | --- | --- | --- |
| DS-R-001 | 830 | 63 | 39 | 35 | 32 |
| DS-R-002 | 830 | 42 | 20 | 9 | 0 |
| DS-R-003 | 650 | 40 | 29 | 10 | 6 |
| DS-R-004 | Control | 51 | 39 | 41 | 42 |
| DS-R-005 | Control | 49 | 14 | 9 | 58 |
| DS-R-006 | 650 | 69 | 66 | 33 | 31 |
| DS-R-007 | 830 | 59 |  |  |  |
| DS-R-008 | 650 | 40 | 39 | 38 | 21 |
| DS-R-009 | 650 | 39 | 37 | 40 | 22 |
| DS-R-010 | Control | 38 | 39 | 36 | 25 |
| DS-R-011 | Control | 50 | 51 | 50 | 49 |
| DS-R-012 | Control | 49 | 38 | 40 | 40 |
| DS-R-013 | Control | 45 |  |  |  |
| DS-R-014 | 650 | 40 | 33 | 17 | 11 |
| DS-R-015 | 650 | 88 | 57 | 16 | 29 |
| DS-R-016 | 830 | 46 | 30 | 23 | 14 |
| DS-R-017 | 830 | 45 | 19 | 10 | 10 |
| DS-R-018 | 830 | 41 | 27 | 22 | 5 |
| DS-R-019 | Control | 59 | 50 | 50 | 39 |
| DS-R-020 | 830 | 56 |  |  |  |
| DS-R-021 | 830 | 49 | 50 |  |  |
| DS-R-022 | Control | 38 | 35 | 30 | 29 |
| DS-R-023 | 650 | 49 | 25 | 28 | 27 |
| DS-R-024 | Control | 43 | 33 | 31 | 45 |
| DS-R-025 | 830 | 70 | 45 | 30 | 10 |
| DS-R-026 | 650 | 50 | 29 | 30 | 30 |
| DS-R-027 | 650 | 50 | 0 | 0 | 48 |
| DS-R-028 | 650 | 45 |  |  |  |
| DS-R-029 | 830 | 40 | 19 | 20 | 10 |
| DS-R-030 | 650 | 50 | 50 | 51 | 51 |
| DS-R-031 | Control | 39 | 33 | 20 | 9 |
| DS-R-032 | 830 | 60 | 54 | 46 | 35 |
| DS-R-033 | Control | 39 | 34 | 27 | 10 |
| DS-R-034 | Control | 58 | 65 | 60 | 60 |
| DS-R-035 | 830 | 68 | 62 | 39 | 40 |
| DS-R-036 | 650 | 54 | 30 | 23 | 24 |
| DS-R-037 | 650 | 40 | 40 | 31 | 31 |
| DS-R-038 | 830 | 70 | 75 | 41 | 10 |
| DS-R-039 | Control | 50 | 50 | 49 | 40 |
| DS-R-040 | 830 | 50 | 35 | 35 | 30 |
| DS-R-041 | Control | 39 | 35 | 19 | 4 |
| DS-R-042 | 650 | 39 | 25 | 10 | 2 |
| DS-R-043 | 650 | 51 | 46 | 36 | 45 |
| DS-R-044 | Control | 59 | 70 | 69 | 69 |
| DS-R-045 | 830 | 59 | 55 | 39 | 32 |

WOMAC total score

|  | Group | WOMAC total at visit 1 | WOMAC total at visit 7 | WOMAC total at visit 13 | WOMAC total at visit 14 |
| --- | --- | --- | --- | --- | --- |
| DS-R-001 | 830 | 82 | 55 | 48 | 41 |
| DS-R-002 | 830 | 60 | 32 | 27 | 24 |
| DS-R-003 | 650 | 64 | 50 | 55 | 52 |
| DS-R-004 | Control | 82 | 64 | 74 | 78 |
| DS-R-005 | Control | 60 | 39 | 30 | 38 |
| DS-R-006 | 650 | 68 | 51 | 31 | 30 |
| DS-R-007 | 830 | 69 |  |  |  |
| DS-R-008 | 650 | 86 | 78 | 68 | 47 |
| DS-R-009 | 650 | 44 | 46 | 48 | 43 |
| DS-R-010 | Control | 48 | 24 | 24 | 24 |
| DS-R-011 | Control | 34 | 31 | 30 | 28 |
| DS-R-012 | Control | 87 | 71 | 51 | 84 |
| DS-R-013 | Control | 77 |  |  |  |
| DS-R-014 | 650 | 64 | 43 | 34 | 30 |
| DS-R-015 | 650 | 98 | 54 | 24 | 24 |
| DS-R-016 | 830 | 60 | 52 | 71 | 26 |
| DS-R-017 | 830 | 71 | 33 | 44 | 39 |
| DS-R-018 | 830 | 62 | 51 | 30 | 28 |
| DS-R-019 | Control | 63 | 43 | 33 | 27 |
| DS-R-020 | 830 | 83 |  |  |  |
| DS-R-021 | 830 | 64 | 62 |  |  |
| DS-R-022 | Control | 73 | 72 | 55 | 55 |
| DS-R-023 | 650 | 77 | 57 | 33 | 26 |
| DS-R-024 | Control | 61 | 40 | 39 | 59 |
| DS-R-025 | 830 | 73 | 51 | 35 | 36 |
| DS-R-026 | 650 | 45 | 43 | 46 | 46 |
| DS-R-027 | 650 | 59 | 42 | 35 | 40 |
| DS-R-028 | 650 | 64 |  |  |  |
| DS-R-029 | 830 | 35 | 36 | 32 | 32 |
| DS-R-030 | 650 | 77 | 79 | 43 | 48 |
| DS-R-031 | Control | 64 | 61 | 37 | 29 |
| DS-R-032 | 830 | 61 | 55 | 57 | 50 |
| DS-R-033 | Control | 52 | 61 | 43 | 30 |
| DS-R-034 | Control | 62 | 65 | 61 | 62 |
| DS-R-035 | 830 | 68 | 50 | 34 | 29 |
| DS-R-036 | 650 | 65 | 46 | 56 | 48 |
| DS-R-037 | 650 | 56 | 54 | 39 | 27 |
| DS-R-038 | 830 | 54 | 37 | 30 | 28 |
| DS-R-039 | Control | 60 | 66 | 68 | 36 |
| DS-R-040 | 830 | 64 | 39 | 45 | 35 |
| DS-R-041 | Control | 31 | 39 | 28 | 25 |
| DS-R-042 | 650 | 47 | 44 | 30 | 28 |
| DS-R-043 | 650 | 54 | 54 | 54 | 54 |
| DS-R-044 | Control | 92 | 84 | 84 | 83 |
| DS-R-045 | 830 | 70 | 61 | 54 | 59 |

WOMAC pain subscale score

|  | Group | WOMAC pain subscale at visit 1 | WOMAC pain subscale at visit 7 | WOMAC pain subscale at visit 13 | WOMAC pain subscale at visit 14 |
| --- | --- | --- | --- | --- | --- |
| DS-R-001 | 830 | 16 | 9 | 7 | 8 |
| DS-R-002 | 830 | 11 | 7 | 6 | 5 |
| DS-R-003 | 650 | 12 | 6 | 7 | 7 |
| DS-R-004 | Control | 19 | 15 | 16 | 16 |
| DS-R-005 | Control | 15 | 8 | 6 | 12 |
| DS-R-006 | 650 | 14 | 8 | 7 | 6 |
| DS-R-007 | 830 | 11 |  |  |  |
| DS-R-008 | 650 | 15 | 14 | 15 | 9 |
| DS-R-009 | 650 | 8 | 9 | 8 | 10 |
| DS-R-010 | Control | 10 | 5 | 5 | 5 |
| DS-R-011 | Control | 7 | 8 | 6 | 5 |
| DS-R-012 | Control | 20 | 16 | 10 | 18 |
| DS-R-013 | Control | 15 |  |  |  |
| DS-R-014 | 650 | 13 | 14 | 6 | 6 |
| DS-R-015 | 650 | 21 | 11 | 5 | 5 |
| DS-R-016 | 830 | 13 | 14 | 15 | 5 |
| DS-R-017 | 830 | 14 | 8 | 11 | 11 |
| DS-R-018 | 830 | 12 | 7 | 6 | 6 |
| DS-R-019 | Control | 11 | 7 | 7 | 5 |
| DS-R-020 | 830 | 15 |  |  |  |
| DS-R-021 | 830 | 13 | 13 |  |  |
| DS-R-022 | Control | 16 | 11 | 9 | 12 |
| DS-R-023 | 650 | 15 | 10 | 6 | 6 |
| DS-R-024 | Control | 12 | 8 | 7 | 11 |
| DS-R-025 | 830 | 15 | 12 | 8 | 8 |
| DS-R-026 | 650 | 12 | 9 | 9 | 9 |
| DS-R-027 | 650 | 14 | 7 | 7 | 9 |
| DS-R-028 | 650 | 13 |  |  |  |
| DS-R-029 | 830 | 7 | 7 | 6 | 6 |
| DS-R-030 | 650 | 15 | 17 | 6 | 10 |
| DS-R-031 | Control | 13 | 10 | 8 | 6 |
| DS-R-032 | 830 | 9 | 13 | 11 | 9 |
| DS-R-033 | Control | 10 | 12 | 8 | 5 |
| DS-R-034 | Control | 12 | 14 | 13 | 13 |
| DS-R-035 | 830 | 15 | 10 | 7 | 5 |
| DS-R-036 | 650 | 13 | 10 | 11 | 10 |
| DS-R-037 | 650 | 8 | 9 | 8 | 6 |
| DS-R-038 | 830 | 8 | 9 | 7 | 6 |
| DS-R-039 | Control | 13 | 13 | 15 | 7 |
| DS-R-040 | 830 | 14 | 7 | 9 | 7 |
| DS-R-041 | Control | 7 | 10 | 6 | 5 |
| DS-R-042 | 650 | 9 | 10 | 5 | 5 |
| DS-R-043 | 650 | 13 | 13 | 13 | 13 |
| DS-R-044 | Control | 18 | 15 | 15 | 15 |
| DS-R-045 | 830 | 16 | 14 | 10 | 10 |

WOMAC function subscale score

|  | Group | WOMAC function subscale at visit 1 | WOMAC function subscale at visit 7 | WOMAC function subscale at visit 13 | WOMAC function subscale at visit 14 |
| --- | --- | --- | --- | --- | --- |
| DS-R-001 | 830 | 59 | 41 | 37 | 29 |
| DS-R-002 | 830 | 45 | 23 | 19 | 17 |
| DS-R-003 | 650 | 48 | 42 | 43 | 41 |
| DS-R-004 | Control | 57 | 45 | 52 | 56 |
| DS-R-005 | Control | 39 | 29 | 22 | 24 |
| DS-R-006 | 650 | 48 | 38 | 22 | 22 |
| DS-R-007 | 830 | 52 |  |  |  |
| DS-R-008 | 650 | 66 | 58 | 47 | 35 |
| DS-R-009 | 650 | 34 | 35 | 36 | 31 |
| DS-R-010 | Control | 33 | 17 | 17 | 17 |
| DS-R-011 | Control | 24 | 21 | 22 | 21 |
| DS-R-012 | Control | 60 | 49 | 37 | 59 |
| DS-R-013 | Control | 56 |  |  |  |
| DS-R-014 | 650 | 45 | 25 | 25 | 22 |
| DS-R-015 | 650 | 69 | 37 | 17 | 17 |
| DS-R-016 | 830 | 42 | 34 | 51 | 19 |
| DS-R-017 | 830 | 52 | 23 | 31 | 26 |
| DS-R-018 | 830 | 44 | 41 | 21 | 20 |
| DS-R-019 | Control | 48 | 33 | 23 | 20 |
| DS-R-020 | 830 | 61 |  |  |  |
| DS-R-021 | 830 | 45 | 43 |  |  |
| DS-R-022 | Control | 51 | 55 | 42 | 39 |
| DS-R-023 | 650 | 56 | 42 | 24 | 17 |
| DS-R-024 | Control | 42 | 27 | 27 | 45 |
| DS-R-025 | 830 | 51 | 34 | 24 | 25 |
| DS-R-026 | 650 | 28 | 32 | 35 | 35 |
| DS-R-027 | 650 | 40 | 33 | 25 | 28 |
| DS-R-028 | 650 | 47 |  |  |  |
| DS-R-029 | 830 | 26 | 27 | 24 | 24 |
| DS-R-030 | 650 | 55 | 55 | 34 | 34 |
| DS-R-031 | Control | 45 | 45 | 25 | 20 |
| DS-R-032 | 830 | 47 | 37 | 39 | 36 |
| DS-R-033 | Control | 38 | 45 | 32 | 22 |
| DS-R-034 | Control | 44 | 47 | 44 | 44 |
| DS-R-035 | 830 | 47 | 36 | 24 | 21 |
| DS-R-036 | 650 | 46 | 32 | 39 | 32 |
| DS-R-037 | 650 | 42 | 39 | 27 | 19 |
| DS-R-038 | 830 | 42 | 25 | 21 | 20 |
| DS-R-039 | Control | 42 | 48 | 47 | 26 |
| DS-R-040 | 830 | 44 | 29 | 33 | 25 |
| DS-R-041 | Control | 22 | 25 | 20 | 18 |
| DS-R-042 | 650 | 34 | 30 | 22 | 19 |
| DS-R-043 | 650 | 35 | 35 | 35 | 35 |
| DS-R-044 | Control | 67 | 62 | 62 | 61 |
| DS-R-045 | 830 | 49 | 43 | 40 | 44 |

EQ-5D-5L score

|  | Group | EQ-5D-5L at visit 1 | EQ-5D-5L at visit 7 | EQ-5D-5L at visit 13 | EQ-5D-5L at visit 14 |
| --- | --- | --- | --- | --- | --- |
| DS-R-001 | 830 | 0.303 | 0.763 | 0.745 | 0.763 |
| DS-R-002 | 830 | 0.707 | 0.837 | 1 | 1 |
| DS-R-003 | 650 | 0.706 | 0.872 | 0.73 | 0.73 |
| DS-R-004 | Control | 0.563 | 0.784 | 0.795 | 0.795 |
| DS-R-005 | Control | 0.795 | 0.883 | 0.883 | 0.795 |
| DS-R-006 | 650 | 0.692 | 0.733 | 0.816 | 0.816 |
| DS-R-007 | 830 | 0.73 |  |  |  |
| DS-R-008 | 650 | 0.657 | 0.73 | 0.772 | 0.804 |
| DS-R-009 | 650 | 0.841 | 0.841 | 0.816 | 0.795 |
| DS-R-010 | Control | 0.73 | 1 | 1 | 1 |
| DS-R-011 | Control | 0.795 | 0.862 | 0.862 | 0.862 |
| DS-R-012 | Control | 0.659 | 0.659 | 0.762 | 0.795 |
| DS-R-013 | Control | 0.74 |  |  |  |
| DS-R-014 | 650 | 0.762 | 0.809 | 0.837 | 0.772 |
| DS-R-015 | 650 | 0.467 | 0.776 | 1 | 0.829 |
| DS-R-016 | 830 | 0.73 | 1 | 1 | 1 |
| DS-R-017 | 830 | 0.689 | 0.795 | 0.841 | 0.841 |
| DS-R-018 | 830 | 0.73 | 0.749 | 0.829 | 1 |
| DS-R-019 | Control | 0.688 | 0.73 | 0.762 | 0.871 |
| DS-R-020 | 830 | 0.646 |  |  |  |
| DS-R-021 | 830 | 0.783 | 0.816 |  |  |
| DS-R-022 | Control | 0.795 | 0.751 | 0.721 | 0.677 |
| DS-R-023 | 650 | 0.676 | 0.73 | 0.717 | 0.783 |
| DS-R-024 | Control | 0.718 | 0.763 | 0.762 | 0.689 |
| DS-R-025 | 830 | 0.473 | 0.505 | 0.784 | 0.795 |
| DS-R-026 | 650 | 0.795 | 0.858 | 0.795 | 0.795 |
| DS-R-027 | 650 | 0.751 | 0.841 | 0.858 | 0.862 |
| DS-R-028 | 650 | 0.646 |  |  |  |
| DS-R-029 | 830 | 0.83 | 0.83 | 0.862 | 1 |
| DS-R-030 | 650 | 0.659 | 0.763 | 0.763 | 0.763 |
| DS-R-031 | Control | 0.73 | 0.733 | 0.851 | 0.851 |
| DS-R-032 | 830 | 0.795 | 0.677 | 0.784 | 0.762 |
| DS-R-033 | Control | 0.816 | 0.851 | 0.795 | 0.862 |
| DS-R-034 | Control | 0.762 | 0.795 | 0.7 | 0.762 |
| DS-R-035 | 830 | 0.677 | 0.689 | 0.795 | 0.809 |
| DS-R-036 | 650 | 0.795 | 0.795 | 0.763 | 0.795 |
| DS-R-037 | 650 | 0.762 | 1 | 0.862 | 1 |
| DS-R-038 | 830 | 0.751 | 0.762 | 0.862 | 0.883 |
| DS-R-039 | Control | 0.718 | 0.689 | 0.763 | 0.703 |
| DS-R-040 | 830 | 0.795 | 0.841 | 0.762 | 0.841 |
| DS-R-041 | Control | 0.751 | 0.762 | 0.862 | 0.871 |
| DS-R-042 | 650 | 0.646 | 0.734 | 1 | 1 |
| DS-R-043 | 650 | 0.763 | 0.763 | 0.763 | 0.795 |
| DS-R-044 | Control | 0.751 | 0.676 | 0.664 | 0.419 |
| DS-R-045 | 830 | 0.677 | 0.763 | 0.763 | 0.73 |

Dosage of rescue medication and PGA scores at Visits 7, 13, and 14

|  | Group | Dosage at visit 7 | Dosage at visit 13 | Dosage at visit 14 | PGA at visit 7 | PGA at visit 13 | PGA at visit 14 |
| --- | --- | --- | --- | --- | --- | --- | --- |
| DS-R-001 | 830 | 5 | 8 | 5 | 4 | 4 | 4 |
| DS-R-002 | 830 | 2 | 0 | 0 | 5 | 5 | 5 |
| DS-R-003 | 650 | 0 | 0 | 0 | 4 | 4 | 4 |
| DS-R-004 | Control | 0 | 0 | 0 | 4 | 4 | 4 |
| DS-R-005 | Control | 0 | 0 | 0 | 4 | 5 | 3 |
| DS-R-006 | 650 | 4 | 0 | 0 | 3 | 5 | 5 |
| DS-R-007 | 830 |  |  |  |  |  |  |
| DS-R-008 | 650 | 4 | 0 | 0 | 3 | 4 | 5 |
| DS-R-009 | 650 | 0 | 0 | 0 | 3 | 4 | 4 |
| DS-R-010 | Control | 0 | 0 | 0 | 3 | 4 | 4 |
| DS-R-011 | Control | 0 | 0 | 0 | 3 | 3 | 3 |
| DS-R-012 | Control | 2 | 4 | 0 | 4 | 4 | 4 |
| DS-R-013 | Control |  |  |  |  |  |  |
| DS-R-014 | 650 | 0 | 3 | 2 | 4 | 4 | 4 |
| DS-R-015 | 650 | 0 | 0 | 0 | 5 | 5 | 5 |
| DS-R-016 | 830 | 0 | 0 | 0 | 4 | 4 | 5 |
| DS-R-017 | 830 | 0 | 1 | 1 | 4 | 4 | 4 |
| DS-R-018 | 830 | 0 | 0 | 0 | 4 | 5 | 5 |
| DS-R-019 | Control | 0 | 0 | 0 | 4 | 4 | 4 |
| DS-R-020 | 830 |  |  |  |  |  |  |
| DS-R-021 | 830 | 0 |  |  | 3 |  |  |
| DS-R-022 | Control | 0 | 0 | 0 | 3 | 4 | 4 |
| DS-R-023 | 650 | 2 | 0 | 0 | 4 | 4 | 5 |
| DS-R-024 | Control | 0 | 0 | 0 | 4 | 4 | 4 |
| DS-R-025 | 830 | 20 | 10 | 10 | 4 | 5 | 4 |
| DS-R-026 | 650 | 10 | 10 | 10 | 4 | 4 | 4 |
| DS-R-027 | 650 | 2 | 2 | 4 | 4 | 5 | 4 |
| DS-R-028 | 650 |  |  |  |  |  |  |
| DS-R-029 | 830 | 4 | 2 | 0 | 4 | 4 | 5 |
| DS-R-030 | 650 | 3 | 10 | 1 | 4 | 5 | 4 |
| DS-R-031 | Control | 0 | 0 | 2 | 3 | 5 | 5 |
| DS-R-032 | 830 | 0 | 0 | 1 | 4 | 5 | 4 |
| DS-R-033 | Control | 0 | 0 | 0 | 4 | 4 | 5 |
| DS-R-034 | Control | 0 | 0 | 0 | 3 | 3 | 3 |
| DS-R-035 | 830 | 0 | 0 | 10 | 4 | 4 | 5 |
| DS-R-036 | 650 | 0 | 0 | 0 | 4 | 4 | 4 |
| DS-R-037 | 650 | 0 | 0 | 0 | 3 | 4 | 4 |
| DS-R-038 | 830 | 0 | 0 | 0 | 4 | 5 | 5 |
| DS-R-039 | Control | 12 | 3 | 0 | 3 | 4 | 3 |
| DS-R-040 | 830 | 1 | 0 | 1 | 4 | 4 | 4 |
| DS-R-041 | Control | 0 | 0 | 0 | 4 | 4 | 5 |
| DS-R-042 | 650 | 0 | 0 | 0 | 4 | 4 | 5 |
| DS-R-043 | 650 | 0 | 3 | 7 | 4 | 5 | 4 |
| DS-R-044 | Control | 0 | 0 | 1 | 4 | 4 | 3 |
| DS-R-045 | 830 | 10 | 1 | 12 | 4 | 4 | 4 |
