# Supplementary material for: Efficacy and safety of invasive laser acupuncture (650 and 830 nm) on knee osteoarthritis: A pilot randomized clinical trial
Source: PLoS One. 2026 Jul 20;21(7):e0353654. doi: 10.1371/journal.pone.0353654 (PMC13384278; doi:10.1371/journal.pone.0353654)
Supplement: S3 File — (PDF) [file pone.0353654.s008.pdf]

A randomized, outcome assessor-blinded, placebo-controlled, prospective single center pilot clinical trial to evaluate the safety and short-term efficacy of different wavelength(650nm,830nm) of invasive laser medical device (Ellise) on knee osteoarthritis

Introduction

Knee osteoarthritis (KOA) is a degenerative joint disease that leads to functional limitation and pain and necessitates long-term management.1,2 Patients with KOA worldwide increased by 122.42%, from 163,910,000 in 1990 to 36,4580,000 in 2019.3 With increasing obesity and aging of the global population, the incidence and years lived with disability, the burden will increase.4

The main objectives of KOA treatment are pain management, functional recovery, and prevention of progression.5,6 Recommendations for KOA can be divided into surgical, pharmacological, and non-pharmacological interventions.7,8 Several guidelines recommended topical non-steroidal anti-inflammatory drugs (NSAIDs) as first-line treatment, exercise, patient education, self-management, weight reduction as core treatments, and intra-articular injections and oral NSAIDs for persistent pain.5,7-10 Owing to the drug-related side effects and limited benefits of conventional pharmacological interventions, it is necessary to develop new treatments that can provide long-lasting pain reduction and functional recovery.11,12

Low-level laser therapy (LLLT) is a therapeutic approach for treating musculoskeletal diseases using light at wavelengths ranging from 632 nm to 904 nm, with an output power of less than 500 mW.13 The therapeutic effects of LLLT may be attributed to nonthermal and photochemical reactions (photobiomodulation).14 LLLT induces intracellular biochemical

changes, such as the release of anti-inflammatory mediators, cellular oxygenation, and neurotransmitters involved in pain modulation.14,15 Laser photobiomodulation with appropriate parameters had a positive effect on cartilage defects in animal models of KOA .16 According to KOA guidelines, laser therapy is not recommended as a core intervention for the treatment of KOA.1,5,9,10,17 This may be due to the controversial clinical efficacy of LLLT in the treatment of KOA.18 Rayegani et al. reported that despite some positive findings, significant heterogeneity in the meta-analysis prevented the conclusion that LLLT significantly improves function and reduces pain in individuals with KOA.19 A systematic review and meta-analysis reported a lack of evidence for the efficacy of LLLT for KOA,20 whereas another meta-analysis demonstrated that LLLT with a 785-860 nm wavelength at 4-8 J per treatment site and 904 nm wavelength at 1-3 J per treatment site is effective in improving function and reducing pain in individuals with KOA. 21 Laser acupuncture (LA), which is noninvasive irradiation of a low-level laser at an acupoint, can be effective in reducing knee pain in individuals with KOA when performed with proper parameters.22

Invasive laser acupuncture (ILA) is a new modality of LLLT, which is a laser irradiation from the tip of an acupuncture needle inserted into the acupoint using an acupuncture needle containing an optic fiber inside and attached to a laser emitting machine.23 A previous study demonstrated that 650 nm ILA inhibited the production of collagenase-induced inflammatory mediators of osteoarthritis in rat models.24 Our previous study revealed that 650 nm ILA significantly reduced pain and improved function in individuals with chronic low back pain. 25

Despite the potential efficacy of ILA for KOA, clinical evidence regarding their usefulness in KOA is insufficient. Therefore, we aim to explore the effects of ILA on KOA through the pilot randomized controlled trial (RCT) described in this manuscript. Our findings will provide a rationale for a rigorous RCT to validate the effects of ILA on KOA.

Methods/Design

Aims

1) This trial aims to explore the efficacy of ILA (830 and 650 nm wavelengths) for KOA in reducing pain, recovering function, and improving the quality of life.

2) We also intend to evaluate the safety of ILA (830 and 650 nm wavelength) in individuals with KOA

Hypothesis

1) ILA (830 and 650 nm wavelengths) will be effective in improving quality of life and functional disability and reducing pain in individuals with KOA.

2) The ILA (830 and 650 nm wavelength) will be a safe therapy for individuals with KOA

Study design and setting

Our trial is a parallel-arm, prospective, single-center, patient-blinded pilot RCT. Forty-five enrolled patients will be equally randomized to the 830, 650, or control groups. All participants will be educated on self-care and exercise. They will undergo the ILA treatment corresponding to their group (control group, sham; 830 group, 830 nm; 650 group, 650 nm wavelength laser) for 10 minutes once daily, twice weekly for six weeks. Efficacy outcomes will be assessed at baseline (week 0), visit after six treatments (week 4), one week after the last treatment (week 7), and six weeks after the last treatment (week 12). The details are presented in Table 1 and Figure 1.

	**STUDY PERIOD**

	Enrollment	Allocation	Post-allocation					Close-out
**TIMEPOINT**	Screening		Visit 1	Visit2-6	Visit 7	Visit8-12	Visit13	Visit 14
	Week		1	1-3	4	4-6	7	12
**ENROLLMENT**								
Informed consent	X							
Sociodemographic profile	X							
Medical history	X							
Vital signs	X	X	X	X	X	X	X	X
Inclusion/exclusion criteria	X							
Allocation		X						
Visual Analogue Scale	X							
**INTERVENTIONS**								
Invasive laser acupuncture (sham, 830, or 650 nm)			X	X	X	X		
Education on self management and exercise			X	X	X	X		
**ASSESSMENTS**								
Change of medical history			X	X	X	X	X	X
Safety assessment (Incidence of AEs)			X	X	X	X	X	X
Clinical laboratory test	X						X	
Visual Analogue Scale			X		X		X	X
European Quality of Life Five Dimension Five Level Scale			X		X		X	X
Western Ontario and McMaster			X		X		X	X

Universities Osteoarthritis Index				
Patient’s Global Assessment	X	X	X	X
Doses of resuce medication	X	X	X	X

Table 1. Study design in accordance with SPIRIT guidelines

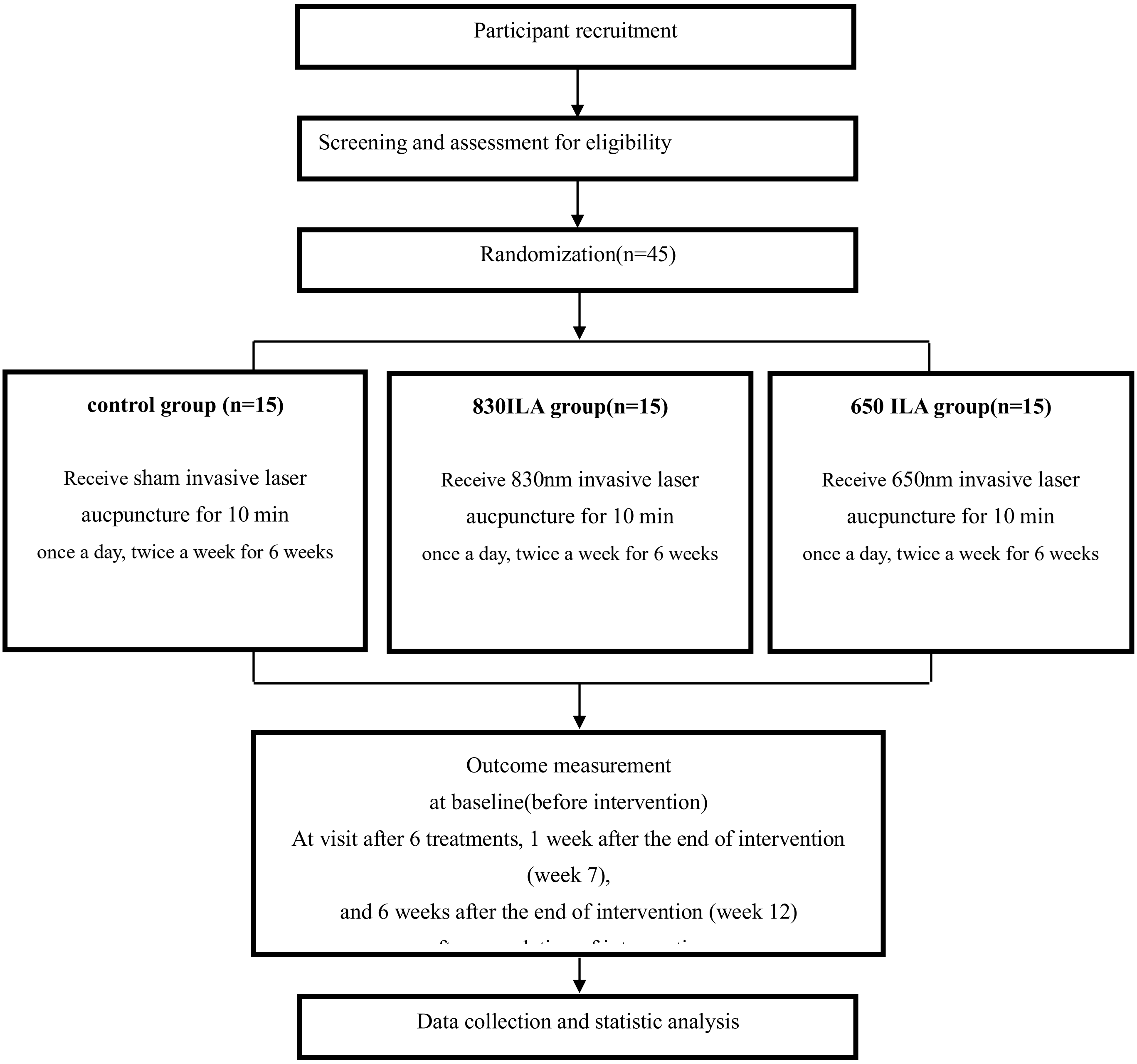

Figure 1. Study design flow chart

Recruitment

Recruitment will be conducted at the Dongshin University Gwangju Korean Medicine

Hospital, Republic of Korea, using advertisements in local newspapers, community posters, and the Internet. The clinical research coordinator (CRC) will explain the purposes and harms of the study to interested individuals, and they will provide voluntarily written informed consent before participating. Patients will be screened using the inclusion and exclusion criteria. The CRC will notify and adjust the schedule at each visit to ensure protocol compliance.

Inclusion criteria

1) Elderly individuals aged between 55 and 85 years, 2) patients diagnosed with KOA in accordance with the American College of Rheumatology diagnostic criteria,27 3) patients with knee pain persisting more than 14 days per month for more than the preceding three months,, 4) presence of moderate knee pain (a mean pain visual analogue scale [VAS] score had a range of 35-74 at rest and during activity during a week prior to screening), 5) patients with stage two or stage three KOA in accordance with Kellgren-Lawrence grade,28 6) voluntary provision of consent.

Exclusion criteria

1) Patients who have undergone total knee replacement, 2) patients with posttraumatic KOA, 3) serious diseases (cancer, diabetic neuropathy, severe liver, kidney cardiovascular, or central nervous system disease), 4) knee pain caused by trauma, tumor, inflammatory disease, autoimmune disease, rheumatoid arthritis, severe hip joint disease, severe bow leg or knock knee, or gout, 5) history of drug or alcohol abuse or mental illness (depression, serious anxiety, or schizophrenia) within six months prior to screening, 6) has been injected with intraarticular mucus supplement (hylan, sodium hyaluronate, and hyalulonan) for the purpose of treating KOA in the 6 months preceding screening, 7) has been injected with steroid into

the knee joint, or drained knee joint fluid, or received prolotherapy in the 3 months preceding screening, 8) currently receiving treatment for KOA, such as Korean medicine treatment, physical therapy, or medication within 4 weeks prior to screening, 9) has other diseases that require treatment for pain control, such as steroids or NSAIDs, 10) not suitable for use of ILA, such as presence of metallic devices in the knee, severe skin disease in the knee region, electronic medical devices, or blood clotting abnormalities, 11) has knee surgery within one year or is scheduled to have knee surgery during the study, 12) are breastfeeding, pregnant, or planning for pregnancy, and 13) participation in other study within 8 weeks prior to screening or participation in other trial.

Violation and dropout criteria

The violation criteria: 1) remarkable protocol violation or serious deviation in implementation or 2) intervention compliance rate of <75% (completed less than 9 of the 12 scheduled intervention sessions).

Dropout criteria: 1) incidence of any serious adverse event (SAE), 2) incomplete data that could have an impact on results, 3) withdrawal of consent for participation, or 4) exclusion from the trial deemed necessary by the principal investigator (PI) or Institutional Review Board (IRB).

Participants who met the dropout or violation criteria will be excluded from the per protocol set analysis.

Ethics

The study has been conducted in accordance with the Declaration of Helsinki (revised in 2013). Our protocol (version. 1.0) was approved by by the Ministry of Food and Drug Safety (date: November 15, 2022; Medical Device Approval # 1427). It (version 1.1) was approved

by the IRB of Dongshin University Gwangju Korean Medicine Hospital (date: July 18, 2023; approval NO: DSGOH-2023-002) and was registered with the Clinical Research Information Service (Registration No. KCT0008860). The CRC will explain the purpose and risk of this trial to the participants and their companions. All the participants provided written informed consent before participation.

Randomization and Allocation

After initial screening and baseline evaluations, forty-five enrolled participants will be equally randomized to the 830, 650, or control groups in accordance with the randomization sequence generated using SAS ® version 9.4 (SAS Institute. Inc., Cary, NC, USA). The random number will be sealed in an opaque envelope and kept in a cabinet with a double lock.

Implementation

Randomization and allocation will be performed by an independent investigator. The enrolment procedures will be conducted by the CRC.

Blinding

Because the practitioner administering the intervention would know the group assignment, a patient- and assessor-blinded design will be adopted. Sham and real lasers have no differences in sound or sensation, and the assessor can only contact the participants at the time of evaluation. Hence, the assessor and participants will not know the group allocation until the trial completion. If SAEs occur, unblinding will be permitted with the approval of the IRB. Only investigators with no conflicts of interest will be included in our trial, and all investigators will be trained in the blinding procedures.

Interventions

The intervention will be administered once daily and twice weekly for six weeks by trained Korean medical doctors. Practitioners who will conduct the intervention will be trained to ensure compliance with the intervention protocol. The intervention will be carried out using medical equipment (Ellise; Wontech Co. Ltd., Daejeon, Republic of Korea) composed of an optical fiber-coupled Laser Diode (830 nm used GaAIAs; 650 nm used InGaAIP), a disposable acupuncture needle with optical fibers inserted inside, and a laser output device.

In supine position with participant's knees bent, the acupuncture needle will be vertically inserted in EX-LE4 (Neixiyan), ST35 (Dubi), ST34 (Liangqiu), SP10 (Xuehai), SP9 (Yinglingquan), GB34 (Yanglingquan), and EX-LE2 (Heding) on the lesion side (the more painful side if bilateral) and then the laser output device will be turned on for 10 min (830 group, 20 mW power; 650 group, 20 mW power; control group, 0 mW power). Manual stimulation will not be conducted. The real laser parameters will be 20 mW power, pulse type wave, 12J/point energy dose, 50 Hz frequency, and 63.69 W/cm2 power density. During the procedures, participants will be blindfolded, and no differences in sounds or feelings will be found among the three groups (Figure 2).

All participants will be educated on self-care and exercise during their treatment visits. Acetaminophen (500 mg) will be provided as rescue medication. The medical conditions of the patients will be monitored at each visit to ensure compliance with the treatment and evaluation procedures. The treatment and assessment schedule will be changed at the request of the patient or as deemed necessary by the investigator.

During the participation, patients will not be permitted to receive other treatments to ameliorate KOA symptoms. However, they will be allowed to take existing medications and receive treatments for other symptoms that do not influence the findings of the trial.

Outcome measures

The primary endpoint will be the changes in VAS scores at rest and during activity (walking on a flat surface) one week after the last treatment (week 7). The secondary outcomes will include the Outcome Measures in Rheumatology Clinical Trials (OMERACT)-Osteoarthritis Research Society International (OARSI)sets of responder rates at one week after last treatment (week 7), Patient Global Assessment (PGA), and doses of rescue medication at visit after six treatments (week 4), one week after last treatment (week 7), and six weeks after last treatment (week 12). The changes in Western Ontario and McMaster Universities Osteoarthritis Index (WOMAC) total, WOMAC function subscale, WOMAC pain subscale, and European Quality of Life Five Dimension Five Level scale (EQ-5D-5L) at visit after six treatments (week 4), one week after last treatment (week 7), and six weeks after last treatment (week 12), and VAS at rest and during an activity at visit after six treatments (week 4) and six weeks after last treatment (week 12) will also be the secondary outcomes.

The VAS is a patient rating scale, a 100 mm-long straight line marked 100 for the worst pain imaginable and 0 for no pain.29 It is commonly used as a primary efficacy endpoint to assess pain intensity in clinical trials of KOA.19-22

The OMERACT-OARSI set of responder criteria are high improvement in function or pain ≥ 50% and absolute change ≥ 20, or improvement in at least two of the three following: 1) PGA ≥ 20% and absolute change ≥ 10; 2) function ≥ 20% and absolute change ≥ 10; 3) pain ≥ 20% and absolute change ≥ 10.30

The WOMAC is a self-administered questionnaire commonly used in KOA that assesses physical function (17 items), pain (5 items), and stiffness (2 items). Each item is scored 0-4. Higher scores indicate increased stiffness, pain, and impaired physical function.31

The PGA is a five-step measurement tool used to quantify disease improvement.32

The EQ-5D-5L is a generic questionnaire used to assess health-related quality of life.33 The quality weights of the EQ-5D-5L will be based on a previous study.34

Safety assessment

The safety outcomes will be the occurrence of adverse events (AEs) and changes in blood chemistry parameters, blood pressure, and pulse rate.

Adverse events

AEs related to laser irradiation did not occur in previous RCT on KOA with LA 19,21 and in our pilot RCT on low back pain with ILA.25 The possible AEs included pallor, dizziness, fainting, skin irritation, bleeding, or local hematoma. The CRC will record all SAEs and AEs, including the severity, time of incidence, treatment procedure, and the relationship between the intervention and AE. All SAEs and AEs will be reported to the IRB and PI and monitored until they stabilize. In the case of SAEs and AEs related to the treatment, participants will be compensated in accordance with the applicable regulations.

Quality control

Experts in LA, statistics, methodology, and KOA have developed and reviewed our protocol. All investigators will receive training before the study to fully understand the protocols and standard operating procedures (SOPs). An independent clinical research associate will check all relevant documents to ensure the trial complies with the protocol and SOPs. Any revisions to the protocol will be reviewed and approved by the IRB.

Sample size

We had no adequate previous or pilot studies from which the sample size could be calculated. Thus, we adopted a pilot study design that considered the limited funds and recruitment opportunities. The appropriate sample size for a three-arm pilot study was >12.[35,36]

As our trial is a pilot study, a formal sample size estimation has not been conducted. Instead, assuming the sample size of our previous pilot study that investigated the efficacy and safety of ILA for nonspecific chronic low back pain,[25] 45 participants (15 per group) will be included. The sample size can be insufficient to determine the effects of ILA on KOA because this trial is a pilot RCT. However, the findings of our study will provide preliminary data for the effects of ILA on KOA and will be used to calculate the sample size for rigorously designed RCT.

Statistical analysis

An independent statistician will analyze the final data using SAS ® version 9.4 (SAS Institute. Inc., Cary, NC, USA). A full analysis set will be used to assess efficacy, and missing values will be filled using the last observation carried forward method. The significance level was set to 5% (two-tailed). Sub-analyses and interim analyses will not be conducted.

The Friedman test or one-way repeated-measures analysis of variance for intragroup comparisons and analysis of covariance with the baseline score as a covariate will be used for intergroup comparisons to analyze changes in efficacy outcomes. The Bonferroni method will be used for significance level correction in the post-hoc tests.

The responder rate and occurrence of AEs among the groups will be compared using Fisher’s exact test or chi-square test. The Kruskal-Wallis test or one-way analysis of variance will be used for intergroup comparisons to analyze changes in blood chemistry parameters, pulse rate, and blood pressure.

Data management and confidentiality

All documents will be recorded and labeled using identification codes to conceal their names. All data will be securely kept in the myTrial data management system (NIKOM, Republic of Korea). Electronic data will be managed by the Data management team, which has no conflicts of interest and is not affiliated with the sponsor. All identification records and data will be only accessible with IRB approval. Written informed consent for the dissemination of personal information will be obtained from all the patients.

Dissemination

We will submit the final data to the IRB and publish the findings of this protocol in a reputed journal following a peer review.

Abbreviations

AEs, adverse events; CRC, clinical research coordinator; EQ-5D-5L, European Quality of Life Five Dimension Five Level scale; HILT, high intensity laser therapy; ILA, invasive laser acupuncture; IRB, Institutional Review Board; KOA, knee osteoarthritis; LA, laser acupuncture; LLLT, low level laser therapy; NSAIDs, non-steroidal anti-inflammatory drugs; OMERACT, Outcome Measures in Rheumatology Clinical Trials; OARSI, Osteoarthritis Research Society International; PGA, Patient Global Assessment; PI, principal investigator; RCT, randomized controlled trial; SAE, serious adverse event; SOPs, standard operating procedures; VAS, visual analogue scale; WALT, World Association of LASER Therapy; WOMAC, Western Ontario and McMaster Universities Osteoarthritis Index.

Footnote

Reporting Checklist

The authors have completed the SPIRIT reporting checklist.

Conflicts of interest

All authors have completed the ICMJE uniform disclosure form. The authors have no conflicts of interest to declare.

References

1. Kolasinski SL, Neogi T, Hochberg MC, et al. 2019 American College of Rheumatology/Arthritis Foundation Guideline for the Management of Osteoarthritis of the Hand, Hip, and Knee. Arthritis Care Res (Hoboken). 2020;72(2):149-162. doi: 10.1002/acr.24131.

2. Sharma L. Osteoarthritis of the Knee. N Engl J Med. 2021;384(1):51-59. doi: 10.1056/NEJMcp1903768.

3. Long H, Liu Q, Yin H, et al. Prevalence Trends of Site-Specific Osteoarthritis From 1990 to 2019: Findings From the Global Burden of Disease Study 2019. Arthritis Rheumatol. 2022;74(7):1172-1183. doi: 10.1002/art.42089.

4. Safiri S, Kolahi AA, Smith E, et al. Global, regional and national burden of osteoarthritis 1990-2017: a systematic analysis of the Global Burden of Disease Study 2017. Ann Rheum Dis. 2020;79(6):819-828. doi: 10.1136/annrheumdis-2019-216515.

5. Brophy RH, Fillingham YA. AAOS Clinical Practice Guideline Summary:

Management of Osteoarthritis of the Knee (Nonarthroplasty), Third Edition. J Am Acad Orthop Surg. 2022;30(9):e721-e729. doi: 10.5435/JAAOS-D-21-01233.

6. Katz JN, Arant KR, Loeser RF. Diagnosis and Treatment of Hip and Knee Osteoarthritis: A Review. JAMA. 2021;325(6):568-578. doi: 10.1001/jama.2020.22171.

7. Arden NK, Perry TA, Bannuru RR, et al. Non-surgical management of knee osteoarthritis: comparison of ESCEO and OARSI 2019 guidelines. Nat Rev Rheumatol. 2021;17(1):59-66. doi: 10.1038/s41584-020-00523-9.

8. Kan HS, Chan PK, Chiu KY, et al. Non-surgical treatment of knee osteoarthritis. Hong Kong Med J. 2019;25(2):127-133. doi: 10.12809/hkmj187600.

9. Bruyère O, Honvo G, Veronese N, et al. An updated algorithm recommendation for the management of knee osteoarthritis from the European Society for Clinical and Economic Aspects of Osteoporosis, Osteoarthritis and Musculoskeletal Diseases (ESCEO). Semin Arthritis Rheum. 2019;49(3):337-350. doi: 10.1016/j.semarthrit.2019.04.008.

10. Bannuru RR, Osani MC, Vaysbrot EE, et al. OARSI guidelines for the non-surgical management of knee, hip, and polyarticular osteoarthritis. Osteoarthritis Cartilage. 2019;27(11):1578-1589. doi: 10.1016/j.joca.2019.06.011.

11. Kloppenburg M, Berenbaum F. Osteoarthritis year in review 2019: epidemiology and therapy. Osteoarthritis Cartilage. 2020;28(3):242-248. doi: 10.1016/j.joca.2020.01.002.

12. Luo X, Liu J, Li Q, Zhao J, Hao Q, Zhao L, Chen Y, Yin P, Li L, Liang F, Sun X. Acupuncture for treatment of knee osteoarthritis: A clinical practice guideline. J Evid Based Med. 2023;16(2):237-245. doi: 10.1111/jebm.12526.

13. Farivar S, Malekshahabi T, Shiari R. Biological effects of low level laser therapy. J

Lasers Med Sci. 2014;5(2):58-62.

14. Cotler HB, Chow RT, Hamblin MR, Carroll J. The Use of Low Level Laser Therapy (LLLT) For Musculoskeletal Pain. MOJ Orthop Rheumatol. 2015;2(5):00068. doi: 10.15406/mojor.2015.02.00068.

15. Tam SY, Tam VCW, Ramkumar S, Khaw ML, Law HKW, Lee SWY. Review on the Cellular Mechanisms of Low-Level Laser Therapy Use in Oncology. Front Oncol. 2020;10:1255. doi: 10.3389/fonc.2020.01255.

16. Xiang A, Deng H, Cheng K, et al. Laser photobiomodulation for cartilage defect in animal models of knee osteoarthritis: a systematic review and meta-analysis. Lasers Med Sci. 2020;35(4):789-796. doi: 10.1007/s10103-019-02937-8.

17. Krishnamurthy A, Lang AE, Pangarkar S, Edison J, Cody J, Sall J. Synopsis of the 2020 US Department of Veterans Affairs/US Department of Defense Clinical Practice Guideline: The Non-Surgical Management of Hip and Knee Osteoarthritis. Mayo Clin Proc. 2021;96(9):2435-2447. doi: 10.1016/j.mayocp.2021.03.017.

18. Ganjeh S, Rezaeian ZS, Mostamand J. Low Level Laser Therapy in Knee Osteoarthritis: A Narrative Review. Adv Ther. 2020;37(8):3433-3449. doi: 10.1007/s12325-020-01415-w.

19. Rayegani SM, Raeissadat SA, Heidari S, Moradi-Joo M. Safety and Effectiveness of Low-Level Laser Therapy in Patients With Knee Osteoarthritis: A Systematic Review and Meta-analysis. J Lasers Med Sci. 2017;8(Suppl 1):S12-S19. doi: 10.15171/jlms.2017.s3.

20. Huang Z, Chen J, Ma J, Shen B, Pei F, Kraus VB. Effectiveness of low-level laser therapy in patients with knee osteoarthritis: a systematic review and meta-analysis. Osteoarthritis Cartilage. 2015;23(9):1437-1444. doi: 10.1016/j.joca.2015.04.005.

21. Stausholm MB, Naterstad IF, Joensen J, et al. Efficacy of low-level laser therapy on pain and disability in knee osteoarthritis: systematic review and meta-analysis of randomised placebo-controlled trials. BMJ Open. 2019;9(10):e031142. doi: 10.1136/bmjopen-2019-031142.

22. Chen Z, Ma C, Xu L, et al. Laser Acupuncture for Patients with Knee Osteoarthritis: A Systematic Review and Meta-Analysis of Randomized Placebo-Controlled Trials. Evid Based Complement Alternat Med. 2019;2019:6703828. doi: 10.1155/2019/6703828.

23. Kim JH, Yang C, Yoo J, et al. Safety and efficacy of 650 nm invasive laser acupuncture on nonspecific chronic low back pain: A protocol for a multicenter randomized placebo-controlled trial. Front Med (Lausanne). 2023;10:1021255. doi: 10.3389/fmed.2023.1021255.

24. Kim MR, Lee YM, Choi DH, Youn DH, Na CS. Effects of laser and electro acupuncture treatment with GB30 · GB34 on Change in Arthritis Rat. Korean Journal of Acupuncture. 2019;36: 189–199. doi: 10.14406/acu.2019.023.

25. Kim JH, Na CS, Cho MR, Park GC, Lee JS. Efficacy of invasive laser acupuncture in treating chronic nonspecific low back pain: A randomized controlled trial. PLoS One. 2022;17(5):e0269282. doi: 10.1371/journal.pone.0269282.

26. Chan AW, Tetzlaff JM, Altman DG, et al. SPIRIT 2013 statement: defining standard protocol items for clinical trials. Ann Intern Med. 2013;158(3):200-7. doi: 10.7326/0003-4819-158-3-201302050-00583.

27. Altman R, Asch E, Bloch D, et al. Development of criteria for the classification and reporting of osteoarthritis. Classification of osteoarthritis of the knee. Diagnostic and Therapeutic Criteria Committee of the American Rheumatism Association. Arthritis Rheum.

1986;29(8):1039-49. doi: 10.1002/art.1780290816.

28. Kohn MD, Sassoon AA, Fernando ND. Classifications in Brief: Kellgren-Lawrence Classification of Osteoarthritis. Clin Orthop Relat Res. 2016;474(8):1886-93. doi: 10.1007/s11999-016-4732-4.

29. Huskisson EC. Measurement of pain. J Rheumatol. 1982;9:768–9. doi: 10.1016/S0140-6736(74)90884-8

30. Pham T, van der Heijde D, Altman RD, et al. OMERACT-OARSI initiative: Osteoarthritis Research Society International set of responder criteria for osteoarthritis clinical trials revisited. Osteoarthritis Cartilage. 2004;12(5):389-99. doi: 10.1016/j.joca.2004.02.001.

31. Collins NJ, Misra D, Felson DT, Crossley KM, Roos EM. Measures of knee function: International Knee Documentation Committee (IKDC) Subjective Knee Evaluation Form, Knee Injury and Osteoarthritis Outcome Score (KOOS), Knee Injury and Osteoarthritis Outcome Score Physical Function Short Form (KOOS-PS), Knee Outcome Survey Activities of Daily Living Scale (KOS-ADL), Lysholm Knee Scoring Scale, Oxford Knee Score (OKS), Western Ontario and McMaster Universities Osteoarthritis Index (WOMAC), Activity Rating Scale (ARS), and Tegner Activity Score (TAS). Arthritis Care Res (Hoboken). 2011;63 Suppl 11(0 11):S208-28. doi: 10.1002/acr.20632.

32. Rohekar G, Pope J. Test-retest reliability of patient global assessment and physician global assessment in rheumatoid arthritis. J Rheumatol. 2009;36(10):2178-82. doi: 10.3899/jrheum.090084.

33. Herdman M, Gudex C, Lloyd A, et al. Development and preliminary testing of the new five-level version of EQ-5D (EQ-5D-5L). Qual Life Res. 2011;20(10):1727-36. doi:

10.1007/s11136-011-9903-x.

34. Kim SH, Ahn J, Ock M, et al. The EQ-5D-5L valuation study in Korea. Qual Life Res. 2016;25(7):1845-52. doi: 10.1007/s11136-015-1205-2.

35. Johanson GA, Brooks GP. Initial scale development: sample size for pilot studies. Educ Psychol Meas. 2010;70: 394-400. doi: 10.1177/0013164409355692.

36. Julious SA. Sample size of 12 per group rule of thumb for a pilot study. Pharm Stat. 2005;4:287-291. doi: 10.1002/pst.185.

37. Law D, McDonough S, Bleakley C, Baxter GD, Tumilty S. Laser acupuncture for treating musculoskeletal pain: a systematic review with meta-analysis. J Acupunct Meridian Stud. 2015;8:2–16. doi: 10.1016/j.jams.2014.06.015.

38. Wu M, Luan L, Pranata A, et al. Is high intensity laser therapy more effective than other physical therapy modalities for treating knee osteoarthritis? A systematic review and network meta-analysis. Front Med (Lausanne). 2022;9:956188. doi: 10.3389/fmed.2022.956188.

39. Cai P, Wei X, Wang W, Cai C, Li H. High-intensity laser therapy on pain relief in symptomatic knee osteoarthritis: A systematic review and meta-analysis. J Back Musculoskelet Rehabil. 2023;36(5):1011-1021. doi: 10.3233/BMR-220228.

40. Joensen J, Ovsthus K, Reed RK, et al. Skin penetration time-profiles for continuous 810 nm and Superpulsed 904 nm lasers in a rat model. Photomed Laser Surg. 2012;30(12):688-94. doi: 10.1089/pho.2012.3306.

41. Chon TY, Mallory MJ, Yang J, Bublitz SE, Do A, Dorsher PT. Laser Acupuncture: A Concise Review. Med Acupunct. 2019;31(3):164-168. doi: 10.1089/acu.2019.1343.

42. Chen N, Wang J, Mucelli A, Zhang X, Wang C. Electro-Acupuncture is Beneficial for Knee Osteoarthritis: The Evidence from Meta-Analysis of Randomized Controlled Trials. Am J Chin Med. 2017;45(5):965-985. doi: 10.1142/S0192415X17500513.

43. Dworkin RH, Turk DC, Farrar JT, et al Core outcome measures for chronic pain clinical trials: IMMPACT recommendations. Pain. 2005;113(1-2):9-19. doi: 10.1016/j.pain.2004.09.012.

임 상 시 험 계 획 서

무릎 골관절염에 대한 의료용레이저조사기(Ellise)의 파장별 (650nm, 830nm) 안전성과 단기 통증 완화 효과를 탐색하기 위한 대조군(거짓레이저), 단일기관, 평가자 눈가림, 무작위배정 전향적 탐색 임상시험

A randomized, outcome assessor-blinded, placebo-controlled, prospective single center pilot clinical trial to evaluate the safety and short-term efficacy of different wavelength(650nm,830nm) of invasive laser medical device (Ellise) on knee osteoarthritis

목 차

1. 임상시험의 명칭
2. 임상시험의 목적 및 배경
3. 임상시험용 의료기기의 개요
4. 대상자의 선정기준·제외기준·인원 및 그 근거
5. 임상시험방법
6. 방문별 임상시험 진행일정
7. 예측되는 부작용 및 사용 시 주의사항
8. 중지·탈락기준
9. 유효성의 평가기준 및 해석방법
10. 부작용을 포함한 안전성의 평가기준·평가방법 및 보고방법
참고문헌

1. 임상시험의 명칭

무릎 골관절염에 대한 의료용레이저조사기(Ellise)의 파장별(650nm, 830nm) 안전성과 단기 통증 완화 효과를 탐색하기 위한 대조군(거짓레이저), 단일기관, 평가자 눈가림, 무작위배정 전향적 탐색 임상시험

2. 임상시험의 목적 및 배경

2.1 임상시험의 목적

본 연구는 '탐색 임상시험'으로서 무릎골관절염에 대해 의료용레이저조사기(Ellise)의 650nm와 830nm레이저 파장과 대조군의 거짓레이저를 비교하여 의료용레이저조사기의 파장별(650nm, 830nm) 안전성과 단기 통증 완화 효과를 탐색함이 목적이다.

본 임상시험의 1차 목적은 의료용레이저조사기(Ellise)의 650nm와 830nm 레이저 파장으로 치료를 받은 시험군과 거짓레이저 치료를 받는 대조군 간의 비교를 통해 Ellise 650nm와 830nm 레이저 치료의 단기 통증 완화 효과를 탐색하는 것이다. 1차 유효성 평가변수는 기저시점 대비 치료종료 (week 6)후 1주(±3일) 시점의 휴식시 통증 (100mm pain Visual Analogue Scale[VAS])의 변화량과 활동시(평지를 걸을 때) 통증 (100mm pain VAS)의 변화량 이다. 2차 유효성 평가변수로 각 군의 기저시점 대비 치료종료 후 1주(±3일) 시점에서 OMERACT-OARSI set에서 반응자(responder)의 범주에 해당하는 반응자의 각 군별 비율차이, 기저시점 대비 6회 치료 후 시점(week 4), 치료종료 후 6주 (week 12) (±3일) 시점의 휴식시 통증 (100mm pain VAS)의 변화량과 활동시(평지를 걸을 때) 통증 (100mm pain VAS)의 변화량, 기저시점 대비 6회 치료 후 시점(week 4), 치료종료 (week 6) 후 1주(±3일) 시점, 치료종료 후 6주 (week 12)(±3일) 시점의 WOMAC total 점수의 변화량, WOMAC pain subscale의 변화량, WOMAC function subscale의 변화량, 시험대상자 만족도 평가(PGA), European Quality of Life Five Dimension. Five Level Scale (EQ-5D-5L)의 변화량, 구제약 투여량을 군 간 비교할 것이다. 그리고, 이상반응, 피부상태, 활력징후, 임상병리검사의 변화를 통해 안전성을 평가할 것이다.

2.2 임상시험의 배경

골관절염(Osteoarthritis, OA)은 국소 염증과 관절의 구조적 변화를 특징으로 하는 흔한 만성 관절 질환으로, 통증, 기능 제한 및 삶의 질 저하를 유발한다. 특히 무릎 골관절염(Knee osteoarthritis, KOA)은 관절연골의 변성 및 마모, 골극 형성, 골경화 등을 동반하며 통증, 강직, 압통, 종창, 마찰음 및 운동장애 등의 증상이 나타날 수 있다 [1-7].

무릎 골관절염 치료의 주요 목표는 통증 조절, 관절 기능 개선, 삶의 질 향상 및 치료 관련 부작용의 최소화이다 [8]. 무릎 골관절염의 치료는 수술적 치료와 비수술적 치료로 구분되며, 비수술적 치료에는 교육, 자가관리, 운동, 체중감량 등의 비약물치료와 비스테로이드성 항염증제(non-steroidal anti-inflammatory drugs, NSAIDs) 등의 약물치료가 포함된다 [2-7]. 그러나 기존의 보존적 치료에 충분히 반응하지 않거나 약물치료의 부작용이 우려되는 경우, Low-level laser therapy(LLLT)와 같은 대체적 치료법이 하나의 선택지가 될 수 있다.

LLLT는 저출력의 단파장 광선을 이용하여 조직에 비열성 광생체조절(photobiomodulation) 효과를 유도하는 치료법이다. LLLT는 세포 내 광수용체를 자극하여 ATP 합성, 산화질소 및 활성산소 조절, 염증매개물질 조절 등에 관여할 수 있으며, 이를 통해 항염증, 조직 회복 및 진통 효과를 나타낼 수 있는 것으로 알려져 있다 [9,10]. 레이저침(Laser Acupuncture, LA)은 LLLT의 일종으로, 침

구학적 이론에 근거하여 경혈 부위에 침 삽입 대신 저출력 레이저를 조사하는 방법이다 [11]. 레이저 치료효과는 조직에 전달되는 에너지량에 영향을 받으므로, 파장, 출력, 에너지량 및 치료부위까지의 전달 효율이 중요하다. 그러나 피부 표면에서 시행하는 비침습 레이저 조사에서는 반사, 산란 및 흡수로 인해 심부 조직까지 충분한 에너지가 전달되기 어려울 수 있다 [9,12].
무릎 골관절염에 대한 LLLT 및 LA의 임상적 효과에 대해서는 아직 일관된 결론이 확립되어 있지 않다. 일부 메타분석에서는 LLLT의 통증 감소 및 기능 개선 효과가 불충분하다고 보고한 반면 [13], 다른 연구에서는 특정 파장과 에너지량을 사용한 LLLT가 무릎 골관절염의 통증과 기능 개선에 효과적일 수 있다고 보고하였다 [10,14]. 또한 LA에 대한 메타분석에서는 적절한 치료 조건에서 단기 통증 감소 효과가 있을 가능성이 제시되었다 [11]. 이러한 근거는 무릎 골관절염에 대한 레이저 기반 치료에서 적절한 광생체자극 효과를 유도할 수 있는 에너지 전달 방법이 중요함을 시사한다.
본 임상시험에서 사용되는 의료용레이저조사기(Ellise)는 광섬유가 삽입된 멸균침(직경 0.3 mm, 길이 30 mm, 내경 0.15 mm)을 이용하여 침 끝에서 레이저를 조사하는 방식의 침습 레이저침 장치이다. 이 장치는 피부 표면에서 발생하는 레이저 에너지의 반사, 산란 및 흡수로 인한 한계를 줄이고, 경혈 및 심부 조직에 레이저 자극을 직접 전달하도록 설계되었다. 본 연구에서는 650 nm와 830 nm 파장을 사용하며, 대조군에는 동일한 침 삽입 절차를 적용하되 레이저 출력은 0 mW로 설정한 거짓레이저를 사용한다.
선행 동물실험에서 650 nm 침습 레이저침은 collagenase로 유발된 골관절염 모델에서 염증매개물질 생성을 억제하고 조직학적 손상을 완화하는 효과를 보였다 [15]. 또한 비특이적 만성요통 환자를 대상으로 한 선행 탐색 임상시험에서 Ellise를 이용한 650 nm 침습 레이저침은 통증 및 기능장애 개선 가능성을 보였고, 650 nm 및 830 nm 파장 모두에서 레이저 조사와 관련된 이상반응은 보고되지 않았다 [16].
따라서 본 탐색 임상시험은 무릎 골관절염 환자를 대상으로 의료용레이저조사기(Ellise)의 650 nm 및 830 nm 침습 레이저침의 안전성과 파장별 단기 통증 완화 효과를 탐색하고, 향후 확증 임상시험 설계를 위한 근거자료를 마련하고자 한다.

3. 임상시험용 의료기기의 개요 (사용목적, 대상질환)

3.1 임상시험용 의료기기의 개요

본 임상시험에서 사용되는 시험기기와 대조기기는 동일하며 다만 군에 따라 의료용레이저조사기(Ellise)로 거짓 레이저, 650nm 파장, 830nm 파장의 레이저를 조사한다는 점에서 차이가 있다. 거짓 레이저와 650nm, 830nm 레이저 시술은 모두 대상자가 특이감각을 느낄 수 없고, 시술과정중에는 임상시험용 의료기기에서 동일한 소리가 나도록 조작하여 대상자 눈가림을 시행한다.

● 모델명 : Ellise
● 제조원 : 원텍㈜
● 모양 및 구조
- 외관 및 구조

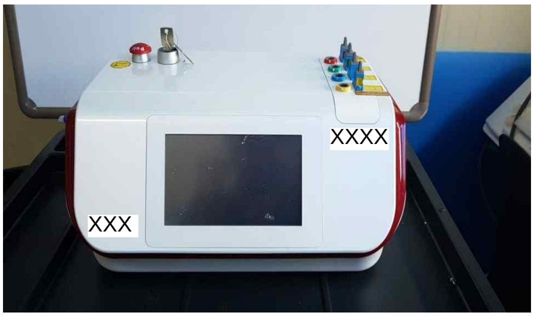	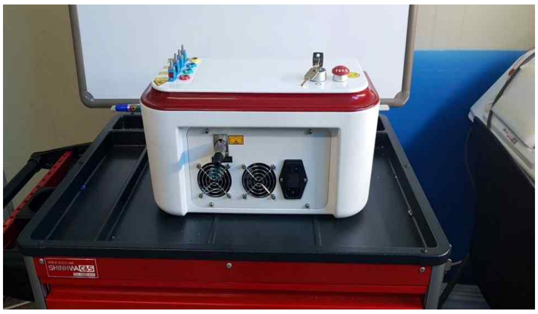
정 면	후 면
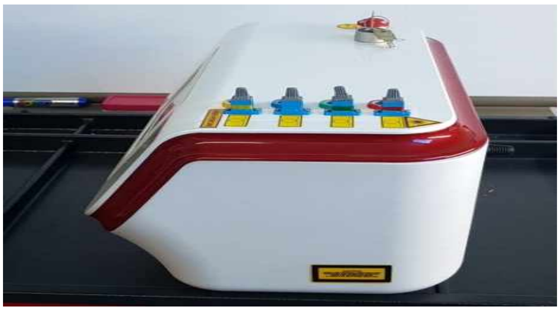	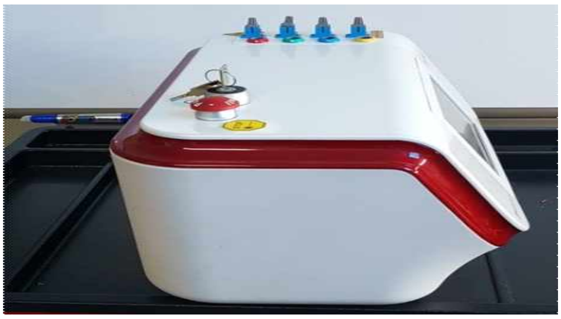
우-측면	좌-측면

- 치수 및 중량
  ① 본체 : 400mm(W) X 259mm(D) X 200mm(H) / 4.58kg
  ② 광섬유 멸균침 - 레이저 전달 장치

기호	명칭	치수
A	총고(mm)	2,000 ± 5%
B	침체 길이(mm)	30 ± 5%
C	침체 지름(mm)	0.30 ± 5%
D	침자루 길이(mm)	20 ± 5%
E	침자루 종류	Spring
	중량(g)	5.4g ± 5%
	침의 내경	0.15mm ± 20%

③ 전원 코드 : 2.8m / 260g

● 주성분(의료용품)

	부분품의 명칭		원재료명 또는 성분명
1	침체		Stainless steel
2	침자루		Stainless steel
3	광섬유 케이블	파이버	Amorphous fused silica
		버퍼	Polyvinyl chloride

● 사용목적 : 의료용레이저조사기(Ellise)의 650, 830nm 레이저파장을 이용하여 무릎 골관절염에 대한 단기 통증 완화에 사용하는 기구

● 대상질환 : 무릎 골관절염

● 저장방법 및 사용기한 : 5℃ ~ 40℃의 상온에서 보관하며, 고온다습한 환경에 저장하지 않는다. 사용기한은 본체는 해당사항 없으며, 광섬유 멸균침의 경우 제조일로부터 2년(24개월)이다.

4. 대상자의 선정기준・제외기준・인원 및 그 근거

4.1 선정기준

1) 만 50세 이상 만 85세 이하의 성인 남녀
2) 스크리닝 기준 3개월 이전에 American College of Rheumatology criteria의 원발성 무릎골관절염의 임상 진단기준 (clinical criteria)에 부합하는자 [17]
   a. 현재 한쪽 혹은 양쪽 무릎의 통증이 있으며,
   b. 다음 6가지 기준 중 최소 3가지 이상 해당하는 자
      ① 만 50세 이상
      ② 무릎관절의 조조강직이 30분 미만인 자(Morning Stiffness)
      ③ 무릎 관절 염발음(Crepitus)
      ④ 압통(Bony tenderness)
      ⑤ 무릎관절 골비대(Bony enlargement)
      ⑥ 촉진상 열감 없음(No palpable warmth)
3) 무릎통증 발생시점이 스크리닝 시점에서 3개월 이상 경과하였고, 통증이 한달에 14일 이상 지속되며, 스크리닝 당시 해당 무릎관절에 대한 지난 1주일간 휴식시와 활동시(평지를 걸을 때) 각각의 일일 평균 통증이 100mm 통증척도(100mm Visual Analogue Scale, VAS) 35mm에서 74mm 사이인 중등도의 무릎통증인 자 [18]
4) 스크리닝에서 방사선촬영(x-ray)을 통해 관절강 협착정도가 Kellgren & Lawrence grade로 2-3 등급인 자 [19]
   - 2등급: 관절강의 적은 감소가 있으면서 최소한 골극 존재 (경증)
   - 3등급: 관절강의 중등도 감소가 있으면서 중등도 골극 존재 (중등도)
5) 임상시험이 시작되기 전에 본 임상시험의 연구 참여 대상자 서면동의서에 자발적으로 서명한 자

4.2 제외기준

1) 퇴행성 무릎관절염으로 대상 무릎에 인공관절치환술을 받은 자
2) 외상 후 2차 유발된 퇴행성 무릎관절염 환자
3) 심각한 기저질환(암, 중증 신장질환, 중증 간장질환, 중증 심혈관계질환, 중증 뇌신경계질환,

당뇨병성 신경병증)이 있는 경우
4) 염증성 질환, 종양, 외상, 류마티스성 관절염, 자가면역질환, 무릎관절의 심한 외반 및 내반 변성, 심한 고관절 질환, 통풍 등 다른 염증성 관절염이 있는 경우
5) 최근 6개월 이내에 알코올 및 약물중독, 심각한 정신과적 병변(치매, 정신분열, 뇌전증)으로 치료받은 자
6) 스크리닝 기준 6개월 이내 무릎 골관절염 치료 목적의 관절강내 점액 보충제 (viscosupplement; hylan, sodium hyaluronate, hyalulonan 등) 주사를 맞은 자
7) 스크리닝 기준 3개월 이내 무릎 관절강내 주사를 통해 관절액을 배출한 자
8) 스크리닝 기준 3개월 이내 무릎 관절강내, 무릎 주위에 프롤로테라피 또는 스테로이드 등의 주사 치료를 받은 자
9) 스크리닝 기준 4주 이내 무릎 골관절염 치료 목적의 한의치료(침, 뜸, 부항, 한약 등), 비약물적 국소치료(물리치료 등), 골관절염 치료제, 비스테로이드성 소염진통제 및 글루코사민을 포함한 관절/연골건강 개선 건강기능식품을 복용하고 있는 자
10) 다른 질환으로 부신피질호르몬제제 및 비스테로이드성 소염진통제 등 통증제어 목적의 치료를 필요로 하는 자
11) 침습 레이저침 시술에 주의가 필요한 자(혈액응고장애, 시술부위의 중증의 피부질환자, 무릎부위에 metalic device가 있는 자, 심전계 등 장착용 전자의료기기를 부착한 자)
12) 최근 1년이내 외상, 연골, 인대손상으로 인해 대상 무릎에 수술을 시행하였거나 수술이 예정되어 있는 자
13) 임신 중이거나 본 임상시험 기간동안 임신 계획이 있는 자 또는 수유부
14) 스크리닝 기준 2개월 이내 타 임상시험에 참여했거나 시험 기간 중에 다른 임상시험에 참가할 계획이 있는 자 (단, 비중재 관찰연구에 참여한 경우, 시험자 및 시험책임자의 판단 하에 참여 가능)
15) 기타 사유로 인하여 시험자 및 시험책임자가 임상시험 참여가 부적합하다고 판단한 자

4.3 인원 및 근거

본 연구는 '안전성 및 유효성 탐색을 위한 탐색 임상시험'으로, 무릎 골관절염 환자에 대한 의료용레이저조사기(Ellise)의 안전성과 파장별 단기 통증 완화효과를 탐색하기 위하여 의료용레이저조사기(Ellise)의 650nm와 830nm의 레이저파장의 단기 통증 완화효과를 대조군의 거짓레이저 치료와 비교하고자 한다. 단기 통증 완화효과를 판단할 수 있는 평가는 휴식시와 활동시(평지를 걸을 때) 100mm pain Visual Analogue Scale(VAS)을 사용하기로 하였다.

본 연구는 안전성과 유효성 탐색을 위한 pilot study로서 pilot study의 경우 군당 최소 12명의 대상자가 필요하다는 기존연구 [20], 연구기간, 임상시험실시기관의 모집 가능성을 고려하여 대조군, 650군, 830군의 3군에 한 군당 15명의 대상자를 배정한다. 따라서 본 임상시험에 필요한 대상자 수는 45명으로 설정한다.

5. 임상시험방법

5.1 임상시험 디자인 및 대상자 배정

1) 디자인 : 무작위배정, 대조군, 평행설계, 평가자눈가림 (평가자, 대상자눈가림)

2) 시험군 : 650군 (650nm 레이저 치료), 830군 (830nm 레이저 치료)
대조군 : 거짓 레이저 치료
3) 눈가림 대상 : 의료기기 시술자 및 무작위배정 봉투 관리자를 제외한 임상시험 연구자 (임상시험책임자 [시술자 및 무작위배정 봉투관리자가 아닌 경우], 유효성 평가자, 임상시험담당자 [시술자 및 무작위배정 봉투 관리자 제외], 공동연구자) 및 대상자
4) 중간분석 : 임상시험 종료 전까지 중간분석을 시행하지 않음

본 임상시험은 의료용레이저조사기(Ellise)의 650 nm 및 830 nm 레이저 파장이 무릎 골관절염의 안전성과 단기 통증 완화 효과에 미치는 영향을 탐색하기 위한 단일기관, 평행군, 무작위배정 탐색 임상시험이다. 의료기기 시술 특성상 시술자의 눈가림은 불가능하였으나, 대상자와 유효성 평가자는 눈가림 상태를 유지하도록 설계하였다.
서면동의를 제공하고 선정·제외기준을 충족한 대상자는 1:1:1 비율로 650 nm 레이저 치료군, 830 nm 레이저 치료군 또는 거짓레이저 대조군에 무작위 배정된다. 각 군은 주 2회, 6주간 총 12회의 치료를 받으며, 전체 임상시험 기간은 치료 및 추적관찰을 포함하여 12주로 설정하였다. 평가는 기저시점, 6회 치료 후(Visit 8), 치료종료 후 1주(±3일, Visit 14), 치료종료 후 6주(±3일, Visit 15)에 시행한다.
모든 대상자에게 치료 방문 시 무릎 골관절염 관련 일상생활 관리 및 운동교육을 제공하고, 필요시 구제약물로 아세트아미노펜 500 mg을 사용할 수 있도록 하였다. 시술자와 무작위배정 봉투 관리자 외에는 배정 정보를 알지 못하도록 하였으며, 중간분석은 시행하지 않는다.

5.2 임상시험용 의료기기 사용방법
1) 사용부위 : 무릎 골관절염의 침치료에 관한 체계적 문헌고찰과 메타분석 [21], 무릎 골관절염의 침치료에 대한 임상시험 [22], 퇴행성 슬관절염 한의표준임상진료지침 [23]에서 무릎 골관절염에 주로 사용되며, 무릎 골관절염의 주요 통증 발현부위인 EX-LE4(내슬안), ST35(독비), SP10(혈해), ST34(양구), SP9(음릉천), GB34(양릉천), EX-LE2(학정) 7곳에 시술한다. (단측 무릎 골관절염의 경우 진단받은 단측하지에, 양측 무릎 골관절염의 경우 가장 심한 통증을 보이는 단측 하지에 시술한다.)

2) 출력파장 : 650nm, 830nm 레이저는 출력강도 : 20㎽, 출력주파수 : 50㎐, wave : pulse wave로 설정한 후 10분/회, 2회/주, 6주간 총 12회 사용부위에 시술한다. 대조군의 거짓레이저는 혈위자입 후 레이저 출력을 0mW로 하여 실제 레이저 치료는 시행하지 않는다. 650nm, 830nm 레이저의 출력, 주파수, wave등은 비특이적 만성요통에 단기 통증 완화효과를 보인 650nm 레이저의 parameter를 참고하여 설정하였다 [16].

3) 의료기기 시술자 : 동신대학교 광주한방병원 소속의 한의사

4) 치료방법
① 대조군 : 광섬유 멸균침을 시술부위에 자입한 후 7개의 광섬유 멸균침에 의료용레이저조사기(Ellise)의 거짓레이저 치료를 시행한다.
② 650군 : 광섬유 멸균침을 시술부위에 자입한 후 7개의 광섬유 멸균침에 의료용레이저조사기(Ellise)의 650nm 레이저 치료를 시행한다.
③ 830군 : 광섬유 멸균침을 시술부위에 자입한 후 7개의 광섬유 멸균침에 의료용레이저조사기

(Ellise)의 830nm 레이저 치료를 시행한다.

혈자리	치료부위	자침깊이
EX-LE4(내슬안)	무릎부위, 무릎뼈 아래모서리, 무릎인대(ligamentum patellae)의 안쪽 오목한 곳	무릎 중앙을 향해 비스듬히 1.5-3 cm
ST35(독비)	무릎 앞쪽면, 무릎인대(patella ligament)의 가쪽 오목한 곳	수직으로 0.9-1.5cm
SP10(혈해)	넓적다리 앞 안쪽면, 안쪽넓은근(vastus medialis muscle)이 튀어나온 곳으로, 무릎뼈바닥(base of patella) 안쪽 끝에서 위쪽으로 2촌 부위	수직으로 1.5-2.4cm<br>비스듬히 3-4.5cm
ST34(양구)	넓적다리 앞가쪽면, 가쪽넓은근(vastus lateralis muscle)과 넙다리곧은근힘줄(rectus femoris tendon) 가쪽모서리사이, 무릎뼈바닥(base of patellae)에서 위쪽으로 2촌	수직으로 1.5-3cm<br>비스듬히 3-4.5cm
SP9(음릉천)	종아리 정강뼈면, 정강뼈 안쪽관절융기 아래모서리와 정강뼈 안쪽모서리(medial border of the tibia)사이의 오목한 곳	수직으로 1.5-3cm<br>비스듬히 3-4.5cm
GB34(양릉천)	종아리의 종아리뼈쪽면에, 종아리뼈 머리(head of the fibula)에서 앞먼쪽(anterior and distal)의 오목한 곳	수직으로 2.4-3.6cm<br>경골 후연을 향해 비스듬히 아래로 3-6cm
EX-LE2(학정)	무릎위, 무릎뼈바닥(base of patellae) 중간지점 위의 오목한 곳	수직으로 1.5-2.4cm

5) 치료일정

각 군은 1일 1회, 1주 2회, 6주(+2주)내에 총 12회의 치료일정을 완료한다. 단 대상자의 건강 상태 및 제반 여건에 따라 주 2회의 치료원칙은 조정될 수 있다.

5.3 무작위배정, 할당은닉, 눈가림 방법

서면동의를 제공하고 선정·제외기준을 충족한 대상자에게는 고유한 대상자 식별코드를 부여한다. 대상자 식별코드는 실시기관 코드, 스크리닝 또는 등록 구분, 일련번호로 구성되며, 한 번 부여된 번호는 재사용하지 않는다.

무작위배정은 블록 무작위배정 방법으로 시행하며, SPSS 프로그램을 이용하여 무작위배정 일련번호를 생성한다. 무작위배정 코드는 투과되지 않는 봉함된 봉투에 넣어 이중 잠금장치가 있는 캐비닛에 보관한다.

무작위배정 봉투 관리자는 대상자 선정, 치료 및 유효성 평가 과정에 관여하지 않는 독립된 담당자로 지정한다. 선정·제외기준 확인 및 치료 전 평가가 완료된 대상자는 Visit 2에서 순서대로 무작위배정 봉투를 개봉하여 650 nm군, 830 nm군 또는 대조군 중 한 군에 배정된다. 배정 정보는 시술

자에게만 전달되며, 대상자와 유효성 평가자는 배정 정보를 알지 못하도록 관리한다.

5.4 중간분석

본 임상시험에서는 사전 정의되지 않은 중간분석을 시행하지 않는다. 임상시험 진행 중에는 필요한 경우를 제외하고 눈가림을 해제하지 않으며, 모든 데이터 수집이 완료되고 데이터베이스가 확정된 후 눈가림 해제 절차를 수행한다. 임상시험 중 생성된 자료는 접근권한을 제한하여 관리하고, 유효성 평가 자료는 눈가림 유지를 위해 다른 임상시험 자료와 분리하여 보관한다.

5.5 임상시험 방법

임상시험담당자는 스크리닝, 치료 및 추적관찰 단계에서 동의서 취득, 선정·제외기준 확인, 활력징후 측정, 병력·약물력·치료력 확인, 유효성 평가, 이상반응 확인, 구제약 복용 여부 및 복용량 확인, 일상생활 관리 및 운동치료 교육 등을 수행한다. 임상시험책임자는 전체 임상시험이 계획서에 따라 수행되도록 감독하며, 필요한 업무를 임상시험담당자에게 위임할 수 있다.
무작위배정 코드를 관리하고 결과를 분석하는 독립적 통계학자, 의료기기 시술자 및 무작위배정 봉투 관리자는 눈가림을 시행하지 않는다. 반면, 시술자와 무작위배정 봉투 관리자를 제외한 임상시험담당자, 임상시험책임자, 공동연구자, 유효성 평가자 및 대상자는 배정 정보에 대해 눈가림을 유지한다.

6. 방문별 임상시험 진행일정

임상시험 평가항목	스크리닝	Treatment phase						7wks (+2주)	F/U
		1wks	2wks	3wks	4wks	5wks	6wks		12wks (+2주)
방문 차수 (Visit)[1]	Visit 1	Visit 2-3	Visit 4-5	Visit 6-7	Visit 8-9	Visit 10-11	Visit 12-13	Visit 14	Visit 15
Visit window[2]	-2주	1-6주 (+2주)						V13+1주 (±3일)	V13+6주 (±3일)
동의서 서명	●								
인구·사회학적 정보[3]	●								
활력 징후	●	●	●	●	●	●	●	●	●
병력·치료력[4]	●								
선정/제외 기준 판정 (방사선검사[5], 신체검진[6])	●								
무작위 배정		●							
임상병리검사[7]	●							●	
임신반응검사[8]	●								
Visual Analogue Scale(VAS) of pain[9]	●	●			●			●	●
Western Ontario and McMaster Universities Osteoarthritis Index (WOMAC)		●			●			●	●

Patient's Global Assessment (PGA)					●			●	●
European Quality of Life Five Dimension Five Level Scale (EQ-5D-5L)		●			●			●	●
구제약 제공[10]		●						●	
구제약 복용 여부 및 복용량 확인[11]					●			●	●
의료용레이저조사기 (Ellise)[12]		●	●	●	●	●	●		
무릎 골관절염 관련 일상생활관리 및 운동 치료 교육[13]		●	●	●	●	●	●		
이상반응 확인[14]		●	●	●	●	●	●	●	●
방문일정 교육		●	●	●	●	●	●	●	
병력, 약물력, 치료력 변화[15]		●	●	●	●	●	●	●	●
증례 결론									●

.

7. 예측되는 부작용

치료에서 발생한 모든 유해하고 의도되지 않은 반응으로서, 임상시험에 사용된 치료와의 인과 관계를 배제할 수 없는 경우를 말한다. 부작용은 사람에 따라 다양하며 이들 부작용을 모두 또는 일부를 경험하거나 전혀 경험하지 않을 수도 있다.
본 임상시험에서 사용되는 의료용레이저조사기(Ellise)는 침습적인 침치료를 시행한 후 레이저 조사가 이뤄지므로 예측되는 부작용은 침치료와 레이저치료시의 부작용과 유사할 것으로 생각된다. 구제약물(아세트아미노펜)과 대상자가 기존에 사용하고 있던 약물은 기존에 해당 약물에 대해 보고된 부작용과 유사할 것으로 생각된다. 본 임상시험 실시 중 발생할 수 있는 부작용과 이에 대한 평가 방법은 다음과 같다.

Table 4. 시험용의료기기와 시술 및 구제약 투여에 따라 예측되는 부작용

	부작용	평가방법
레이저조사기	무릎 골관절염에 시술시 보고된 특이 이상반응 없음 [10, 14]	문진과 육안으로 평가
침습적인 침치료	자침 부위의 통증, 국소출혈이나 혈종, 피부의 과민 반응, 감염, 국소감염증후(발적, 종창, 국소통증) 오심, 구토, 두통, 어지러움 [24,25]	문진과 육안으로 평가
구제약 투여	심혈관계 위험(심혈관계 혈전 반응, 심근경색증 및 뇌졸중), 위장관계 위험(위장관 출혈, 궤양, 천공, 설사, 복통, 변비, 오심), 중추신경계(두통, 어지러움), 간담도계(AST, ALT 상승), 피부계(고정발진), 기타 과민반응 등	문진과 육안으로 평가<br>필요시 임상병리 검사

* 대상자마다 다를 수 있는 기존 복용 중인 약물의 예측되는 부작용은 해당 약물의 기존에 보고된 부작용 정보에 의거하여 판단함
* 비특이적 만성요통에 대한 650nm, 830nm 레이저 파장의 유효성 및 안전성을 탐색한 탐색임상연구 [16]에서 대조군에서 혈종 1건과 오심 1건의 예측되는 부작용이 발생했으나 2건 모두 경증으로 치료 없이 회복되었으며, 레이저 조사로 인한 의료기기이상반응은 발생하지 않음

이러한 부작용 경험 시 임상시험 담당 한의사 또는 시험책임자는 해당 증상을 치료하거나 경험한 불편을 해소하기 위해 다른 치료를 제공할 수 있다.
예측되는 부작용이 발생하였을 때에는 그 정도에 따라 3단계로 구분하고 시험자는 부작용명 및 그 정도를 증례기록서에 기록한다.

(1) 경증(mild) : 부작용의 정도가 경미한 경우
(2) 중등도(moderate) : 부작용의 정도가 중등도인 경우
(3) 중증(Severe) : 부작용의 정도가 심한 경우

8. 중지・탈락기준

임상시험 중 부작용, 이상반응 또는 기타 대상자의 안전을 위협할 수 있는 상황이 발생한 경우 시험책임자의 판단에 따라 임상시험을 일시 중지하거나 중단할 수 있다. 임상시험이 중지된 경우 시험책임자는 관련 자료를 기록·보관하고, 필요한 의학적 조치와 추적관찰이 이루어지도록 한다.
대상자가 참여 동의를 철회한 경우, 중대한 이상반응이 발생한 경우, 이상반응으로 인해 연구자 또는 대상자가 연구 중단이 필요하다고 판단한 경우, 무작위배정 이후 유효성 평가자료가 수집되지 않은 경우, 추적이 불가능한 경우, 또는 기타 시험책임자가 임상시험 지속이 부적합하다고 판단한 경우 조기탈락할 수 있다. 중도탈락 시 탈락 사유와 탈락 전까지 수집된 자료를 기록·보관하며, 중지 및 탈락 대상자는 새로운 대상자로 대체하지 않는다. 단, 타당한 이유가 없는 한 중도탈락자의 자료는 유효성 및 안전성 분석에 포함한다.
선정기준 위반, 제외기준 해당, 연구자 또는 대상자의 중대한 연구계획 위반, 또는 치료 순응도 75% 미만(총 12회 치료 중 9회 미만)인 경우 중대한 계획서 위반으로 판단하여 PPS 분석에서 제

외한다. 경미한 계획서 위반은 위반 내용과 사유를 기록하고, 연구 결과에 미친 영향을 검토하여 PPS 분석 포함 여부를 결정한다.

9. 유효성의 평가기준, 해석방법

9.1 유효성 평가기준

1) 일차 유효성 평가변수

일차 유효성 평가변수는 기저시점 대비 치료종료 후 1주(±3일) 시점의 휴식시 통증(100 mm Pain VAS) 변화량과 활동시 통증(평지를 걸을 때의 100 mm Pain VAS) 변화량이다.

2) 이차 유효성 평가변수

이차 유효성 평가변수는 다음과 같다.

치료종료 후 1주(±3일) 시점의 OMERACT-OARSI responder rate

6회 치료 후 시점 및 치료종료 후 6주(±3일) 시점의 휴식시 및 활동시 통증(100 mm Pain VAS) 변화량

6회 치료 후 시점, 치료종료 후 1주(±3일) 시점 및 치료종료 후 6주(±3일) 시점의 WOMAC total, WOMAC pain subscale, WOMAC function subscale 변화량

6회 치료 후 시점, 치료종료 후 1주(±3일) 시점 및 치료종료 후 6주(±3일) 시점의 시험대상자 만족도 평가(PGA), EQ-5D-5L 변화량 및 구제약 투여량

OMERACT-OARSI responder는 다음 중 하나를 충족하는 대상자로 정의한다: 1) WOMAC pain 또는 function subscale이 50% 이상, 동시에 100점 만점 기준 20점 이상 호전된 경우; 또는 2) WOMAC pain, WOMAC function, PGA 중 두 항목 이상에서 각각 20% 이상, 동시에 100점 만점 기준 10점 이상 호전된 경우 [26].

9.2 해석방법

본 임상시험의 통계분석은 본 연구의 치료 및 평가 과정에 참여하지 않은 독립적 통계학자가 수행한다. 임상시험 종료 전에는 사전 정의되지 않은 중간분석을 시행하지 않는다.

분석군은 ITT, FAS, PPS 및 Safety set으로 구분한다. ITT는 무작위배정 후 최소 한 번 이상 임상시험용 의료기기를 적용받은 모든 대상자로 정의한다. FAS는 ITT 원칙에 따라 무작위배정 후 최소 한 번 이상 의료기기를 적용받고, 기저시점을 포함하여 적어도 한 번 이상 유효성 평가자료가 수집된 대상자로 구성한다. PPS는 FAS 중 계획된 치료와 평가를 완료하고 중대한 계획서 위반이 없는 대상자로 구성한다. Safety set은 무작위배정 후 최소 한 번 이상 임상시험용 의료기기를 적용받은 모든 대상자로 정의한다.

유효성 분석은 FAS를 주 분석군으로 하고 PPS 분석을 보조적으로 시행한다. 안전성 분석은 Safety set을 대상으로 한다. FAS 분석에서 결측치가 발생한 경우, 결측 시점 이전의 가장 최근 관측값으로 대체하는 LOCF(Last Observation Carried Forward) 방법을 적용한다.

연속형 변수는 대상자 수, 평균, 표준편차, 중앙값, 25% 및 75% 사분위수로 제시하고, 범주형 변수는 빈도와 백분율로 제시한다. 통계적 검정은 양측검정으로 시행하며, 유의수준은 $p<0.05$로 설정한다.

인구학적 및 치료 전 임상적 특성은 FAS를 대상으로 기술통계량을 제시하고 세 군 간 비교를 시행한다. 범주형 변수는 카이제곱 검정 또는 피셔의 정확검정을 사용하고, 연속형 변수는 정규성 여부

에 따라 one-way ANOVA 또는 Kruskal-Wallis test를 사용한다.
일차 유효성 평가변수인 기저시점 대비 치료종료 후 1주(±3일) 시점의 휴식시 및 활동시 100 mm Pain VAS 변화량은 군별 기술통계량을 제시하고, 기저시점 VAS 값을 공변량으로 포함한 ANCOVA model을 이용하여 군 간 비교한다. 필요 시 유효성 평가변수에 영향을 미칠 수 있는 인구학적 또는 임상적 특성을 공변량으로 추가할 수 있다. 대조군 대비 650 nm군 및 830 nm군의 least-square mean 차이, 양측 95% 신뢰구간 및 p-value를 제시하며, 사후검정 시 유의수준 보정은 Bonferroni 방법을 적용한다.
연속형 이차 유효성 평가변수는 일차 유효성 평가변수와 동일한 방식으로 분석한다. OMERACT-OARSI responder rate는 군별 빈도와 백분율을 제시하고, 카이제곱 검정 또는 피셔의 정확검정을 이용하여 군 간 비교한다.

10. 부작용을 포함한 안전성의 평가기준 · 평가방법 및 보고방법

안전성 평가는 Safety set을 대상으로 시행한다. Safety set은 무작위배정 이후 최소 한 번 이상 임상시험용 의료기기를 적용받은 모든 대상자로 정의한다. 안전성 평가항목은 이상반응 및 중대한 이상반응의 발생, 의료기기와의 관련성, 피부상태, 활력징후, 임상병리검사 결과로 구성된다.
이상사례(adverse event, AE)는 임상시험 중 대상자에게 발생한 모든 의도하지 않은 증후, 증상 또는 질병을 의미하며, 임상시험용 의료기기와 반드시 인과관계가 있어야 하는 것은 아니다. 의료기기이상반응(adverse device effect, ADE)은 임상시험용 의료기기와의 인과관계를 배제할 수 없는 유해하고 의도하지 않은 반응으로 정의한다. 중대한 이상사례 또는 중대한 의료기기이상반응은 사망, 생명 위협, 입원 또는 입원기간 연장, 영구적 또는 중대한 장애·기능저하, 태아 기형 또는 이상, 기타 의학적으로 중요한 상황을 초래한 경우로 정의한다.
이상반응의 중증도는 경증, 중등도, 중증으로 구분한다. 경증은 별도의 처치가 필요하지 않고 정상 생활 또는 기능을 크게 저해하지 않는 경우, 중등도는 정상 생활 또는 기능을 유의하게 저해하거나 처치가 필요할 수 있는 경우, 중증은 고도의 처치가 필요하거나 후유증이 남을 수 있는 경우로 정의한다. 임상시험용 의료기기와의 인과관계는 명확히 관련 있음, 관련이 있다고 생각됨, 관련 가능성 있음, 관련이 없다고 생각됨, 명확히 관련 없음, 불명으로 평가한다.
시험담당자는 임상시험 기간 동안 매 방문 시 이상반응 발생 여부, 병력·약물력·치료력 변화, 피부상태 및 활력징후를 확인한다. 임상병리검사는 계획된 평가시점에 시행하며, 임상적으로 의미 있는 이상치가 확인되는 경우 시험책임자의 판단에 따라 추가 평가 또는 재검사를 시행할 수 있다. 스크리닝 시점의 임상병리검사 이상치는 이상반응으로 수집하지 않으며, 치료 이후 새로 발생하거나 악화된 이상치 중 증상을 동반하거나 임상적으로 의미 있다고 판단되는 경우 이상반응으로 기록한다.
이상반응 발생 시 시험담당자는 발생일, 증상, 중증도, 처치, 경과 및 임상시험용 의료기기와의 인과관계를 증례기록서에 기록하고 시험책임자에게 보고한다. 중대한 이상사례 또는 중대한 의료기기이상반응이 발생한 경우에는 관련 규정과 기관 절차에 따라 의뢰자 및 임상시험심사위원회에 보고하고, 해당 이상반응이 소실되거나 추적이 불가능해질 때까지 추적관찰한다. 필요한 경우 시험책임자는 대상자의 안전을 위해 임상시험용 의료기기 적용 중단, 추가 치료 또는 임상시험 중단 여부를 결정한다.
안전성 분석에서는 이상반응을 경험한 대상자 수와 비율을 군별로 제시하고, 의료기기와 관련성이 있다고 판단된 이상반응을 별도로 요약한다. 활력징후 및 임상병리검사 결과는 기술통계량으로 제시하며, 필요 시 정규성 여부에 따라 ANOVA, Kruskal-Wallis test, 카이제곱 검정 또는 피셔의 정확검정을 이용하여 군 간 비교를 시행한다.

참고문헌

1. Katz JN, Arant KR, Loeser RF. Diagnosis and Treatment of Hip and Knee Osteoarthritis: A Review. JAMA. 2021 Feb 9;325(6):568-578. doi: 10.1001/jama.2020.22171.
2. Kolasinski SL, Neogi T, Hochberg MC, Oatis C, Guyatt G, Block J, Callahan L, Copenhaver C, Dodge C, Felson D, Gellar K, Harvey WF, Hawker G, Herzig E, Kwoh CK, Nelson AE, Samuels J, Scanzello C, White D, Wise B, Altman RD, DiRenzo D, Fontanarosa J, Giradi G, Ishimori M, Misra D, Shah AA, Shmagel AK, Thoma LM, Turgunbaev M, Turner AS, Reston J. 2019 American College of Rheumatology/Arthritis Foundation Guideline for the Management of Osteoarthritis of the Hand, Hip, and Knee. Arthritis Rheumatol. 2020 Feb;72(2):220-233. doi: 10.1002/art.41142.
3. Brophy RH, Fillingham YA. AAOS Clinical Practice Guideline Summary: Management of Osteoarthritis of the Knee (Nonarthroplasty), Third Edition. J Am Acad Orthop Surg. 2022 May 1;30(9):e721-e729. doi: 10.5435/JAAOS-D-21-01233.
4. Bannuru RR, Osani MC, Vaysbrot EE, Arden NK, Bennell K, Bierma-Zeinstra SMA, Kraus VB, Lohmander LS, Abbott JH, Bhandari M, Blanco FJ, Espinosa R, Haugen IK, Lin J, Mandl LA, Moilanen E, Nakamura N, Snyder-Mackler L, Trojian T, Underwood M, McAlindon TE. OARSI guidelines for the non-surgical management of knee, hip, and polyarticular osteoarthritis. Osteoarthritis Cartilage. 2019 Nov;27(11):1578-1589. doi: 10.1016/j.joca.2019.06.011.
5. Arden NK, Perry TA, Bannuru RR, Bruyère O, Cooper C, Haugen IK, Hochberg MC, McAlindon TE, Mobasheri A, Reginster JY. Non-surgical management of knee osteoarthritis: comparison of ESCEO and OARSI 2019 guidelines. Nat Rev Rheumatol. 2021 Jan;17(1):59-66. doi: 10.1038/s41584-020-00523-9.
6. Krishnamurthy A, Lang AE, Pangarkar S, Edison J, Cody J, Sall J. Synopsis of the 2020 US Department of Veterans Affairs/US Department of Defense Clinical Practice Guideline: The Non-Surgical Management of Hip and Knee Osteoarthritis. Mayo Clin Proc. 2021 Sep;96(9):2435-2447. doi: 10.1016/j.mayocp.2021.03.017.
7. Kan HS, Chan PK, Chiu KY, Yan CH, Yeung SS, Ng YL, Shiu KW, Ho T. Non-surgical treatment of knee osteoarthritis. Hong Kong Med J. 2019 Apr;25(2):127-133. doi: 10.12809/hkmj187600.
8. Korean knee society subcommittee on osteoarthritis guidelines. Guidelines for the treatment of osteoarthritis of the knee. J Korean Knee Soc. 2010;22(1):69-73.
9. 장인수외 역. 레이저치료학. 서울; 도서출판 정담, 2006; 75-78, 110-112, 380-385
10. Rayegani SM, Raeissadat SA, Heidari S, Moradi-Joo M. Safety and Effectiveness of Low-Level Laser Therapy in Patients With Knee Osteoarthritis: A Systematic Review and Meta-analysis. J Lasers Med Sci. 2017 Summer;8(Suppl 1):S12-S19. doi: 10.15171/jlms.2017.s3.
11. Chen Z, Ma C, Xu L, Wu Z, He Y, Xu K, Moqbel SAA, Wu L. Laser Acupuncture for Patients with Knee Osteoarthritis: A Systematic Review and Meta-Analysis of Randomized Placebo-Controlled Trials. Evid Based Complement Alternat Med. 2019 Nov 3;2019:6703828.

doi: 10.1155/2019/6703828.
12. Chon TY, Mallory MJ, Yang J, Bublitz SE, Do A, Dorsher PT. Laser Acupuncture: A Concise Review. Med Acupunct. 2019 Jun 1;31(3):164-168. doi: 10.1089/acu.2019.1343.
13. Huang Z, Chen J, Ma J, Shen B, Pei F, Kraus VB. Effectiveness of low-level laser therapy in patients with knee osteoarthritis: a systematic review and meta-analysis. Osteoarthritis Cartilage. 2015 Sep;23(9):1437-1444. doi: 10.1016/j.joca.2015.04.005.
14. Stausholm MB, Naterstad IF, Joensen J, Lopes-Martins RÁB, Sæbø H, Lund H, Fersum KV, Bjordal JM. Efficacy of low-level laser therapy on pain and disability in knee osteoarthritis: systematic review and meta-analysis of randomised placebo-controlled trials. BMJ Open. 2019 Oct 28;9(10):e031142. doi: 10.1136/bmjopen-2019-031142.
15. 김미래, 이유미, 최동희, 윤대환, 나창수, 환도,양릉천 레이저 및 전침이 관절염에 미치는 영향. Korean Journal of acupuncture. 2019;36(4):189-199. doi.org/10.14406/acu.2019.023.
16. Kim JH, Na CS, Cho MR, Park GC, Lee JS. Efficacy of invasive laser acupuncture in treating chronic non-specific low back pain: A randomized controlled trial. PLoS One. 2022 May 31;17(5):e0269282. doi: 10.1371/journal.pone.0269282.
17. Altman R, Asch E, Bloch D, Bole G, Borenstein D, Brandt K, Christy W, Cooke TD, Greenwald R, Hochberg M, et al. Development of criteria for the classification and reporting of osteoarthritis. Classification of osteoarthritis of the knee. Diagnostic and Therapeutic Criteria Committee of the American Rheumatism Association. Arthritis Rheum. 1986 Aug;29(8):1039-49. doi: 10.1002/art.1780290816.
18. Boonstra AM, Schiphorst Preuper HR, Balk GA, Stewart RE. Cut-off points for mild, moderate, and severe pain on the visual analogue scale for pain in patients with chronic musculoskeletal pain. Pain. 2014 Dec;155(12):2545-2550. doi: 10.1016/j.pain.2014.09.014.
19. Kohn MD, Sassoon AA, Fernando ND. Classifications in Brief: Kellgren-Lawrence Classification of Osteoarthritis. Clin Orthop Relat Res. 2016 Aug;474(8):1886-93. doi: 10.1007/s11999-016-4732-4.
20. Julious SA. Sample size of 12 per group rule of thumb for a pilot study. Pharmaceut. Statist. 2005;4:287-291. doi.org/10.1002/pst.185.
21. Tian H, Huang L, Sun M, Xu G, He J, Zhou Z, Huang F, Liu Y, Liang F. Acupuncture for Knee Osteoarthritis: A Systematic Review of Randomized Clinical Trials with Meta-Analyses and Trial Sequential Analyses. Biomed Res Int. 2022 Apr 21;2022:6561633. doi: 10.1155/2022/6561633.
22. Zhang Q, Fang J, Chen L, Wu J, Ni J, Liu F, Sun J. Different kinds of acupuncture treatments for knee osteoarthritis: a multicentre, randomized controlled trial. Trials. 2020 Mar 14;21(1):264. doi: 10.1186/s13063-019-4034-8.
23. 한의표준임상진료지침 개발사업단. 퇴행성 슬관절염 한의표준임상진료지침. 파주: 군자출판사. 2021; 3,5
24. Furuse N, Shinbara H, Uehara A, Sugawara M, Yamazaki T, Hosaka M, Yamashita H. A Multicenter Prospective Survey of Adverse Events Associated with Acupuncture and Moxibustion in Japan. Med Acupunct. 2017 Jun 1;29(3):155-162. doi: 10.1089/acu.2017.1230.
25. Zhao L, Zhang FW, Li Y, Wu X, Zheng H, Cheng LH, Liang FR. Adverse events

associated with acupuncture: three multicentre randomized controlled trials of 1968 cases in China. Trials. 2011 Mar 24;12:87. doi: 10.1186/1745-6215-12-87.
26. Pham T, van der Heijde D, Altman RD, Anderson JJ, Bellamy N, Hochberg M, Simon L, Strand V, Woodworth T, Dougados M. OMERACT-OARSI initiative: Osteoarthritis Research Society International set of responder criteria for osteoarthritis clinical trials revisited. Osteoarthritis Cartilage. 2004 May;12(5):389-99. doi: 10.1016/j.joca.2004.02.001.
