# Supplementary material for: Efficacy and safety of invasive laser acupuncture (650 and 830 nm) on knee osteoarthritis: A pilot randomized clinical trial
Source: PLoS One. 2026 Jul 20;21(7):e0353654. doi: 10.1371/journal.pone.0353654 (PMC13384278; doi:10.1371/journal.pone.0353654)
Supplement: Attachment — Submitted filename: renamed_f8e96.docx [file pone.0353654.s010.docx]

**POINT -BY -POINT RESPONSES**

Dear editor and reviewers

We thank you for reviewing our manuscript and for your constructive comments, which have helped improve the manuscript. We have carefully reviewed the comments and have accordingly revised the manuscript. All changes are highlighted yellow in the revised manuscript. Below is a detailed, point-by-point response to your comments. We hope that our revisions meet your expectations.

**Additional Editor Comments:**

**Control design: The study lacks a non-acupoint laser control group, making it difficult to determine whether effects are acupoint-specific or due to nonspecific laser stimulation.**

**Response:** We apologize for the confusion

The objective of our study is to explore the effects of non-specific invasive laser stimulation (650 nm and 830 nm) on KOA, rather than focusing on acupoint-specific effects. Therefore, all three groups received acupuncture at the same acupoints. Following this, the control group received a sham laser, while the experimental groups received 650 nm and 830 nm laser stimulation, respectively. (Page 10, Line 166-168)

**BMI imbalance: Significant baseline BMI differences (p = 0.0072) were not adjusted for; this could confound the outcomes.**

**Response**: We appreciate your insightful comment regarding the baseline BMI difference

In accordance with the suggestion, we have re-analyzed our data using Analysis of Covariance (ANCOVA), with BMI included as a covariate for all primary and secondary outcomes.

After adjustment for BMI, the primary outcome remained statistically significant among the three groups, consistent with the original analysis. Although minor changes were observed in some secondary outcomes, the overall interpretation and main conclusions of the study were not materially altered.

Based on the re-analysis results, we have revised the relevant tables (Table 3, 4 and 5) and updated the corresponding sections of the manuscript accordingly. (Page 12, Line 255-256; Page 16, Lines 284-286)

**Sample size: The small sample (n = 45) limits statistical power and generalizability; findings should be interpreted as preliminary.**

**Response:** We thank you for pointing this out

We have explicitly acknowledged this limitation in the revised manuscript. Furthermore, we have strengthened our conclusion (Page 24, Line 429-433) to emphasize that "well-designed studies with larger sample sizes are required to validate these preliminary findings."

(Page 22, Line 407-413)

First, we conducted only a pilot single-center study owing to the limitations of trial duration, research funding, and opportunities for recruitment. Therefore, the sample size and diversity were insufficient to fully validate the efficacy and safety of ILA in the treatment of KOA. These factors may have introduced bias into the trial results. In future studies, multi-center trials with larger sample sizes should be conducted to encompass a more diverse patient population, thereby enhancing the external validity and clinical applicability of the results.

(Page 24, Line 429-433)

The ILA at 650 and 830 nm suggested the potential of short-term improvement in pain reduction among patients with KOA. Additionally, partial but exploratory short-term improvements were observed in pain-related functional disability and quality of life. However, as we conducted a pilot study with a small sample size, further well–designed studies with larger sample are required to validate these preliminary findings.

**Missing data handling: Use of LOCF is suboptimal; more robust methods such as multiple imputation or mixed-effects modeling are recommended**.

**Response:** We appreciate the reviewer’s valuable methodological suggestion

In this study, we employed the Last Observation Carried Forward (LOCF) method because it has been utilized in previous clinical trials investigating the effects of laser therapy or acupuncture for KOA [1,2]. Our study protocol was originally designed and approved by the Institutional Review Board (IRB) based on the LOCF approach, following these established precedents.

While we adhered to the pre-specified protocol for the primary analysis, we acknowledge that LOCF is not the most robust method for handling missing data. Accordingly, we will adopt more advanced statistical methods, such as multiple imputation or mixed-effects models, in the design of our future large-scale trials.

**Interpretation of results: The conclusions appear overstated given the pilot design; results should be described more cautiously as exploratory.**

**Response:** We completely agree with your feedback.

We have revised the conclusion to use more conservative and exploratory language, as suggested. We have also explicitly stated that these preliminary findings should be validated with larger sample size.

(Page 24, Line 429-433)

The ILA at 650 and 830 nm suggested the potential of short-term improvement in pain reduction among patients with KOA. Additionally, partial but exploratory short-term improvements were observed in pain-related functional disability and quality of life. However, as we conducted a pilot study with a small sample size, further well–designed studies with larger sample are required to validate these preliminary findings.

**Journal requirements:**

When submitting your revision, we need you to address these additional requirements.

**1. Please ensure that your manuscript meets PLOS ONE's style requirements, including those for file naming. The PLOS ONE style templates can be found at**

**https://journals.plos.org/plosone/s/file?id=wjVg/PLOSOne_formatting_sample_main_body.pdf and**

**https://journals.plos.org/plosone/s/file?id=ba62/PLOSOne_formatting_sample_title_authors_affiliations.pdf**

**Response:** We apologize for the oversight.

We have checked PLOS ONE’s style requirements again and have ensured that our manuscript meets those requirements, including those for file naming.

**2. We note that you have selected “Clinical Trial” as your article type. PLOS ONE requires that all clinical trials are registered in an appropriate registry (the WHO list of approved registries is at “https://www.who.int/clinical-trials-registry-platform/network/primary-registries" “https://www.who.int/clinical-trials-registry-platform/network/primary-registries and more information on trial registration is at http://www.icmje.org/about-icmje/faqs/clinical-trials-registration/). Please state the name of the registry and the registration number (e.g. ISRCTN or ClinicalTrials.gov) in the submission data and on the title page of your manuscript. a) Please provide the complete date range for participant recruitment and follow-up in the methods section of your manuscript. b) If you have not yet registered your trial in an appropriate registry, we now require you to do so and will need confirmation of the trial registry number before we can pass your paper to the next stage of review. Please include in the Methods section of your paper your reasons for not registering this study before enrolment of participants started. Please confirm that all related trials are registered by stating: “The authors confirm that all ongoing and related trials for this drug/intervention are registered”. Please seehttp://journals.plos.org/plosone/s/submission-guidelines#loc-clinical-trials for our policies on clinical trials.**

Response: We regret the oversight in the original manuscript.

The name of the registry and the registration number have been added to the title page of the manuscript.

(Page 2; Line 33-34)

Trial registration: CRIS registration number: KCT0008860; registered on 10/11/2023 (https://cris.nih.go.kr/cris/search/detailSearch.do/25506).

The complete date range for participant recruitment and follow-up has been moved from the Results (Participants) section to the Methods (Participant Recruitment) section.

(Page 8; Line 126-127)

Participants were recruited through community posters from October 4, 2023 to April 1, 2024 and were treated and followed up from October 10, 2023 to June 26, 2024.

We have added the statement to the Ethical Considerations subsection of the Methods section:

(Page 8; Line 122)

The authors confirm that all ongoing and related trials for this intervention are registered.

**3. Thank you for stating the following financial disclosure:**

[This work was supported by a grant (RS-2023-KH139215) from the Korea Health Technology R&D Project through the Korea Health Industry Development Institute (KHIDI), funded by the Ministry of Health and Welfare, Republic of Korea.].

Please state what role the funders took in the study. If the funders had no role, please state: "The funders had no role in study design, data collection and analysis, decision to publish, or preparation of the manuscript."

If this statement is not correct you must amend it as needed.

Please include this amended Role of Funder statement in your cover letter; we will change the online submission form on your behalf.

**Response:** Thank you for help.

As recommended, we added “The funders had no role in study design, data collection and analysis, decision to publish, or preparation of the manuscript” in our cover letter to change the online submission form (Resubmission cover letter)

**4. Your ethics statement should only appear in the Methods section of your manuscript. If your ethics statement is written in any section besides the Methods, please move it to the Methods section and delete it from any other section. Please ensure that your ethics statement is included in your manuscript, as the ethics statement entered into the online submission form will not be published alongside your manuscript.**

**Response:** We greatly appreciate your specific and constructive guidance.

We have ensured that the ethics statement is included exclusively within the "Ethical Considerations" subsection of the Methods section. (Page 8, 113-122)

**5. If the reviewer comments include a recommendation to cite specific previously published works, please review and evaluate these publications to determine whether they are relevant and should be cited. There is no requirement to cite these works unless the editor has indicated otherwise.**

**Response:** Thank you for the useful advice.

We have noted the instruction and will carefully evaluate and incorporate any recommended citations only if they are relevant to the study.

**6. Please review your reference list to ensure that it is complete and correct. If you have cited papers that have been retracted, please include the rationale for doing so in the manuscript text, or remove these references and replace them with relevant current references. Any changes to the reference list should be mentioned in the rebuttal letter that accompanies your revised manuscript. If you need to cite a retracted article, indicate the article’s retracted status in the References list and also include a citation and full reference for the retraction notice.**

**Response:** We have reviewed our reference list and ensured that it is complete and correct. We confirm that no retracted papers have been cited in this manuscript.

**Reviewer #1:** This pilot randomized clinical trial investigates the efficacy and safety of invasive laser acupuncture (ILA) at wavelengths of 650 nm and 830 nm compared to a sham control in patients with knee osteoarthritis (KOA). The study employs a robust design, adhering closely to CONSORT and SPIRIT guidelines, including proper randomization and effective blinding of patients and outcome assessors. Clinically relevant outcomes such as pain (VAS), functional impairment (WOMAC), and quality of life (EQ-5D-5L) were assessed using validated tools. Findings indicate significant short-term improvements in pain relief, functional outcomes, and quality of life with ILA treatment, and no substantial adverse events were reported. However, I have the following concerns:

**1. The small sample size (n=45) is adequate for a pilot study but insufficient for drawing definitive conclusions regarding efficacy and safety. I would recommend explicitly acknowledging this limitation in the manuscript, providing clear justification for the chosen sample size, and including preliminary sample-size estimations for future large-scale studies based on the variability and effect sizes observed.**

**Response:** We fully agree with the point you have raised.

We have explicitly stated the justification for the chosen sample size and acknowledged that it is insufficient to fully evaluate the efficacy and safety of the intervention (Page 13, Line 233-239).

And, we have added a new subsection titled "Preliminary sample size estimation for future studies." In this section, we provided a calculated sample size for future large-scale trials based on the effect sizes and variability observed in the current study.

(Page 20; Line 339-347)

**Preliminary sample size estimation for future studies**

Following our pilot trial, in which the 830 nm ILA group showed a larger mean change in the primary outcome compared with the 650 nm group, we calculated the sample size for a future larger study focused on the 830 nm ILA. Based on the differences in VAS score changes observed in the pilot trial, we conservatively set the mean difference (standard deviation) between the control and 830 nm groups at 12 (17) for the sample size estimation. With a 1:1 allocation ratio, 90% power, and a two-tailed significance level of 5%, 43 participants per group (86 in total) would be required. Considering the 15% dropout rate observed in the pilot trial, we estimated that 102 participants (51 per group) would need to be recruited.

**2. The use of the last observation carried forward (LOCF) method for handling missing data may introduce bias and potentially affect the validity of findings. I suggest employing multiple imputation or conducting sensitivity analyses to examine the robustness of the results to different methods of missing-data handling, thereby strengthening the credibility of your conclusions.**

**Response:** We appreciate the reviewer’s valuable methodological suggestion

In this study, we employed the Last Observation Carried Forward (LOCF) method because it has been utilized in previous clinical trials investigating the effects of laser therapy or acupuncture for KOA [1,2]. Our study protocol was originally designed and approved by the Institutional Review Board (IRB) based on the LOCF approach, following these established precedents.

While we adhered to the pre-specified protocol for the primary analysis, we acknowledge that LOCF is not the most robust method for handling missing data. Accordingly, we will adopt more advanced statistical methods, such as multiple imputation or mixed-effects models, in the design of our future large-scale trials.

**3. Due to the intervention’s nature, investigators delivering the treatment were not blinded, potentially introducing performance bias. I recommend clearly discussing this limitation and proposing strategies, such as independent outcome assessments or objective outcome measures, to mitigate bias in future trials.**

**Response:** We sincerely thank you for your constructive feedback regarding performance bias.

As suggested, we have revised the limitation section (in the discussion) to more clearly acknowledge the difficulty of blinding practitioners due to the nature of the ILA intervention.

To mitigate this potential bias, we adopted an outcome assessor-blinded design, ensuring that outcome evaluations were conducted by assessors who were not involved in the treatment delivery. We have also clarified in the Discussion that future trials should explore strategies to blind practitioners where feasible and consider incorporating independent outcome assessments or more objective outcome measures to further reduce potential bias.

(Page 23, Line 416-422)

Third, owing to the characteristics of ILA, it was not possible to blind the practitioners, as they had to directly operate the laser device. To minimize this risk, we adopted an outcome assessor-blinded design. Nevertheless, the absence of practitioner blinding remains a methodological limitation and may have influenced the trial results. Future studies should explore strategies to blind practitioners when possible and consider the inclusion of independent outcome assessments or more objective outcome measures to further mitigate potential bias.

**4. There was a significant baseline imbalance in body mass index (BMI) among groups (p=0.0072), which could confound the outcomes related to pain and function. I suggest explicitly addressing this issue in the manuscript and performing analyses adjusted for BMI to account for possible confounding effects.**

**Response**: We appreciate your insightful comment regarding the baseline BMI difference

In accordance with the suggestion, we have re-analyzed our data using Analysis of Covariance (ANCOVA), with BMI included as a covariate for all primary and secondary outcomes.

After adjustment for BMI, the primary outcome remained statistically significant among the three groups, consistent with the original analysis. Although minor changes were observed in some secondary outcomes, the overall interpretation and main conclusions of the study were not materially altered.

Based on the re-analysis results, we have revised the relevant tables (Table 3, 4 and 5) and updated the corresponding sections of the manuscript accordingly. (Page 12, Line 255-256; Page 16, Lines 284-286)

**5. Although significant differences were observed in primary outcomes (VAS and WOMAC), the responder rate defined by OMERACT-OARSI criteria was not significantly different among groups, raising questions regarding clinical significance. I recommend clearly interpreting these results in the context of minimal clinically important differences (MCID) to enhance clinical relevance and interpretability.**

**Response:** We agree that the recommended addition/change significantly strengthens the manuscript.

As suggested, we have expanded the Discussion section to interpret our results using the Minimal Clinically Important Improvement (MCII). We have clarified that despite the non-significant responder rate, the improvement in primary outcomes (VAS and WOMAC pain subscale) surpassed the MCII thresholds. These findings suggest that ILA treatment may offer clinically meaningful improvements for patients with KOA, even if those improvements do not meet the OMERACT-OARSI responder criteria.

(Page 22-23; Line 393-406)

The responder rates were 26.67% in the control group, 53.33% in the 650 group, and 60% in the 830 group. Although the experimental groups showed numerically higher rates compared to the control group, the difference did not reach statistical significance. This lack of significance may be attributed to the small sample size. Minimal clinically important improvement (MCII), defined as the smallest change in measurement that signifies an important improvement in a patient’s symptom, for KOA is established at -19.9 mm for pain VAS and -9.1 for the WOMAC function subscale [40]. In our study, the 650 group showed reductions of -20.7 mm in VAS at rest, -21.7 mm in VAS during activity, and -14.9 in the WOMAC function subscale. Similarly, the 830 group showed reductions of -22.6 mm in VAS at rest, -22.9 mm in VAS during activity, and -14.9 in the WOMAC function subscale. These improvements observed in both experimental groups exceeded the established MCII thresholds. Despite the lack of a statistically significant difference in responder rates, exceeding MCII thresholds suggested potential clinical relevance. Nevertheless, further well-designed studies with larger sample sizes are necessary to substantiate these findings.

**6. The short follow-up period (6 weeks post-intervention) limits assessment of the sustained effects of ILA treatment. I suggest clearly addressing this limitation and recommending longer follow-up durations (3–6 months or longer) in future studies to determine long-term treatment effects.**

**Response:** We thank the reviewer for highlighting this important limitation

As suggested, we have clearly addressed this in the Limitation section of the Discussion and emphasized the necessity for extended follow-up durations (e.g., 3 to 6 months or longer) in future studies to better determine the long-term clinical outcomes.

(Page 23, Line 413-416)

Second, the follow-up duration in our study was relatively short. Consequently, this study has limitations in assessing sustained efficacy, long-term safety, and potential AEs of ILA. To determine the long-term efficacy and safety of ILA, future studies should incorporate extended follow-up periods such as three to six months or longer.

**7. The evaluation of safety data is limited due to the study’s small sample size and short duration. I recommend explicitly noting this limitation in the manuscript and advising larger-scale trials with extended follow-up periods to robustly assess treatment safety and potential adverse events.**

**Response:** We appreciate this comment, as it has allowed us to provide a more balanced view.

As recommended, we have revised the first and second paragraphs of the Limitation section to explicitly acknowledge that the small sample size and short follow-up period limit the robustness of safety assessment and the detection of potential adverse events. We have also emphasized the need for larger, multi-center trials with extended follow-up durations to more comprehensively evaluate long-term safety outcomes as per your suggestion.

(Page 23; Line 407-416)

Despite these advantages, our study has several limitations. First, we conducted only a pilot single-center study owing to the limitations of trial duration, research funding, and opportunities for recruitment. Therefore, the sample size and diversity were insufficient to fully validate the efficacy and safety of ILA in the treatment of KOA. These factors may have introduced bias into the trial results. In future studies, multi-center trials with larger sample sizes should be conducted to encompass a more diverse patient population, thereby enhancing the external validity and clinical applicability of the results. Second, the follow-up duration in our study was relatively short. Consequently, this study has limitations in assessing sustained efficacy, long-term safety, and potential AEs of ILA. To determine the long-term efficacy and safety of ILA, future studies should incorporate extended follow-up periods such as three to six months or longer.

**8. This was a single-center study, limiting the generalizability of the results. I suggest clearly discussing this limitation and recommending that future research involve multicenter studies with more diverse patient populations to improve external validity and applicability of the findings.**

**Response:** We thank the reviewer for pointing this out**.**

As suggested, we have revised the first paragraph of the Limitation section in the Discussion to clearly address that this was a pilot single-center study conducted under constraints related to funding and trial duration. We have also emphasized that future research should involve multi-center studies with larger and more diverse patient populations to enhance external validity and the broader applicability of the findings.

(Page 23; Line 407-413)

First, we conducted only a pilot single-center study owing to the limitations of trial duration, research funding, and opportunities for recruitment. Therefore, the sample size and diversity were insufficient to fully validate the efficacy and safety of ILA in the treatment of KOA. These factors may have introduced bias into the trial results. In future studies, multi-center trials with larger sample sizes should be conducted to encompass a more diverse patient population, thereby enhancing the external validity and clinical applicability of the results.

**Reviewer #2**:

**To meet STRICTA Guidelines:**

**1. Identify depth of insertion**

**2. Name as well as number acupuncture points**

**3. Description of participating acupuncturists (qualification or professional affiliation, years in acupuncture practice, other relevant experience)**

**Response:** We apologize for our ignorance and sincerely thank the reviewer for pointing out the need to align with the STRICTA guidelines.

As suggested, we have added a comprehensive STRICTA checklist as S1 Table and information in manuscript (Page 11, Line 196-198).

**S1 Table. Revised Standards for Reporting Intervention in Clinical Trials of Acupuncture (STRICTA)**

|  | **Item Criteria** | **Description** |
| --- | --- | --- |
| 1.Acupuncture rationale | 1a) Style of acupuncture | Laser acupuncture |
|  | 1b) Reasoning for treatment provided – based on historical context, literature sources, and/or consensus methods, with references where appropriate | 1) Discussion among three doctors that practice Korean medicine (consensus)  2) Selection of treatment regions based on related papers and expert discussions [20,21] |
|  | 1c) Extent to which treatment varied | Standardized treatment |
| 2. Details of needling | 2a) Number of needle insertions per subject per session (mean and range where relevant) | 7 |
|  | 2b) Names (or location if no standard name) of points used (uni-/bilateral) | EX-LE4 (Neixiyan), ST35 (Dubi), ST34 (Liangqiu), SP10 (Xuehai), SP9 (Yinglingquan), GB34 (Yanglingquan), and EX-LE2 (Heding) on the lesion side (the more painful side if bilateral) |
|  | 2c) Depth of insertion, based on a specified unit of measurement or on a particular tissue level | Acupuncture needle was inserted vertically or obliquely at a depth of 1–3 cm, depending the location of the needle. |
|  | 2d) Responses sought | No de qi or muscle twitching,– only sensation due to needle insertion |
|  | 2e) Needle stimulation | The real laser parameters will be 20 mW power, pulse type wave, 12J/point energy dose, 50 Hz frequency, and 63.69 W/cm^2^ power density. |
|  | 2f) Needle retention time | 10-minute per session |
|  | 2g) Needle type | disposable acupuncture needle with optical fibers inserted inside |
| 3. Treatment regimen | 3a) Number of treatment sessions | 12 |
|  | 3b) Frequency and duration of treatment sessions | twice/week for 6weeks, 10-min per session |
| 4. Other treatment components | 4a) Details of other interventions administered to the acupuncture group | education on exercise and self-care during their treatment visits |
|  | 4b) Setting and context of treatment – including instructions to practitioners – as well as information and explanations given to patients | Practitioners who will conduct the intervention will be trained to ensure adherence to the intervention protocol. All patients will be educated on exercise and self-care during their treatment visits. |
| 5. Practitioner background | 5a) Description of participating acupuncturists | Korean medicine doctor with the following qualifications: 6 years of formal university training in Korean medicine, a license |
| 6. Control or comparator interventions | 6a) Rationale for the control or comparator in the context of the research question, with sources that justify the choice | Relevant articles [20,21] |
|  | 6b) Precise description of the control or comparator; details for items 1−3 above with the use of sham acupuncture or any other type of acupuncture-like control | In supine position with participant’s knees bent, the acupuncture needle will be inserted in EX-LE4 (Neixiyan), ST35 (Dubi), ST34 (Liangqiu), SP10 (Xuehai), SP9 (Yinglingquan), GB34 (Yanglingquan), and EX-LE2 (Heding) on the lesion side (the more painful side if bilateral) and then the laser output device will be turned on for 10 min (830 group, 20mW power; 650 group, 20mW power; control group, 0mW power). During the procedures, participants will be blindfolded, and no differences in sounds or feelings will be found among the three groups |

**Language/clarity issues:**

**Line 67: Define invasive laser acupuncture – in intro – needle size, diode used, etc.**

**Response:** We thank the reviewer for this helpful suggestion.

As suggested, we have added a detailed description of the device and needle specifications in the Introduction

(Page 4-5, Line 78-82)

Invasive laser acupuncture (ILA), invasive irradiation and acupuncture stimulation using an optical fiber-coupled laser diode (InGaAIP 650 nm; GaAIAs 830 nm) inserted inside the needle (material, stainless steel; length, 30 mm; inner diameter, 0.15 mm; external diameter, 0.3 mm) demonstrated that the 650 nm ILA restricted the formation of inflammatory mediators in osteoarthritis [13].

**Line 127 "daily mean pain visual analogue scale [VAS] score during activity and at rest over the past week up to screening is between 35 and 74." VAS is usually a score of 0-10. If you are using a 0-100 VAS, this needs to be explained here.**

**Response:** We apologize for not having explained it and creating the confusion.

We used a 100 mm Visual Analogue Scale (VAS) with a range of 0 to 100. To clarify this, we have revised the text to specify the scale

(Page 9, Line 136-137)

(4) existence of moderate knee pain (daily mean pain 0-100 mm visual analogue scale [VAS] score during activity and at rest over the past week up to screening is between 35 and 74)

**Line 158 assigned to three groups (control, 650, or 830) – need to add nm not just the number. And "control" should also be expressed as "0"**

**Response:** Thank you for the suggestion to clarify.

The terms "control," "650," and "830" were originally used as group labels; however, to improve clarity we have revised the text to explicitly indicate the corresponding wavelengths.

(Page 10; Line 166-168)

the 45 recruited participants were randomly assigned to three groups (control [0 mW power sham laser], 650 [20 mW power, 650 nm wavelength laser], or 830 [20 mW power, 830 nm wavelength laser])

**Line 165: During the period of this trial, the assessor did not approach any participant except the assessment time – this is unclear. What is meant by approach? Interact? Speak? See?**

**Response:** We thank the reviewer for this helpful comment.

Our intention was to state that the outcome assessor had no contact with participants outside of the scheduled assessment sessions in order to maintain blinding. To improve clarity, we have revised the wording from “did not approach any participant” to “was not in contact with any participant” in the revised manuscript.

(Page 10, Line 174-176)

During the period of this trial, the assessor was not in contact with any participant except for the time of assessment. Thus, until trial completion

**258: 45 participants survived and... SURVIVED is the wrong word - perhaps replace with "were accepted as trial participants"**

**Response:** We thank the reviewer for pointing this out.

Following your helpful suggestion, we have revised the sentence to more accurately describe the recruitment process.

**“**45 participants were accepted as trial participants” (Page 14, Line 266)

**Line 329: asthenia – are you using this to indicate feeling faintness? Was this during treatment?**

**Response:** Thank you for your pointing regarding the term "asthenia."

We would like to clarify that this did not refer to feeling faintness during treatment. Rather, it was intended to describe a non-specific sense of general weakness or a decrease in physical condition during the treatment period, without a particular underlying cause.

To avoid potential ambiguity and improve clarity, we have replaced the term "asthenia" with "general weakness" in the revised manuscript (Page 19, Line 329-330).

**In your discussion, you suggest significant outcomes. Then in your Sample Size Calculation, you correctly note that sample size was too limited to evaluate effectiveness. be clearer about the issue with sample size in the discussion.**

**Response:** We are grateful for this critical feedback, which has helped improve the clarity and consistency of our manuscript.

We agree that the small sample size limits the ability to draw definitive conclusions regarding clinical effectiveness, despite the observed statistical significance.

Accordingly, we have revised the Limitation section in the Discussion and Conclusion to more explicitly state that this was a pilot study and our findings should be interpreted as preliminary. We have clarified that although significant improvements were observed in primary outcomes, the current sample size (n=45) is insufficient to definitively validate clinical efficacy. These results are intended to inform and justify future adequately powered, large-scale trials.

(Page 23, Line 407-413)

Despite these advantages, our study has several limitations. First, we conducted only a pilot single-center study owing to the limitations of trial duration, research funding, and opportunities for recruitment. Therefore, the sample size and diversity were insufficient to fully validate the efficacy and safety of ILA in the treatment of KOA. These factors may have introduced bias into the trial results. In future studies, multi-center trials with larger sample sizes should be conducted to encompass a more diverse patient population, thereby enhancing the external validity and clinical applicability of the results.

(Page 24, Line 428-433)

**Conclusions**

The ILA at 650 and 830 nm suggested the potential for a short-term improvement in pain reduction among patients with KOA. Additionally, partial but exploratory short-term improvements were observed in pain-related functional disability and quality of life. However, as we conducted a pilot study with a small sample size, further well–designed studies with larger sample are required to validate these preliminary findings.

**Reviewer #3**:

**1. The text indicates that rescue medications were administered during Visit 1 and Visit 13. Were these provided to all participants, or exclusively to those exhibiting severe symptoms? If limited to a subset of participants, could this have influenced the observed outcomes?**

**Response:** We thank the reviewer for raising this important point.

Rescue medication (20 tablets) was provided to all participants at both Visit 1 and Visit 13 to ensure equal availability across groups and participants. Additional medication was available upon request for participants with persistent symptoms; however, no participants requested or received additional doses during the trial. To avoid confusion, we have revised manuscript.

(Page 11; Line 199-200)

Rescue medications (acetaminophen 500 mg 20 tablets) were provided to all participants at Visit 1 and Visit 13. Dosage of rescue medication was monitored at Visits 7, 13, and 14.

Adherence and the total amount of rescue medication used were strictly monitored at Visits 7, 13, and 14, and no significant differences were observed in the dosage of rescue medication among the three groups. Therefore, the use of rescue medication is unlikely to have influenced the observed treatment outcomes

**2. This study employed a relatively small sample size. Is the comparison involving 15 cases statistically significant? Please provide supporting references or statistical justification for this finding.**

**Response:** We fully agree with your feedback.

As described in the Methods section, this study was designed as a pilot trial, and the sample size was determined based on established studies for a pilot study [3, 4]. Following these references and allowing for an anticipated 20% dropout rate, 15 participants were allocated to each group.

The citation [28, 29] in manuscript has been moved one sentence forward to clarify reference.

(Page 13; Line 233-236)

We adopted a pilot study design due to the absence of previous studies, limited budgets, and enrollment opportunities. In a three-arm pilot study, the adequate sample size is more than 12 [28, 29]. Assuming that 20% of the participants may drop out, we allocated 15 participants to each group.

**3. Differences in body mass index exist across the three groups. Given that clinical practice widely acknowledges an association between body weight and KOA, were potential confounding effects related to BMI adjusted for in the analysis? Additionally, can the robustness of the statistical methods employed be confirmed?**

**Response:** We appreciate your insightful comment regarding the baseline BMI difference

In accordance with the suggestion, we have re-analyzed our data using Analysis of Covariance (ANCOVA), with BMI included as a covariate for all primary and secondary outcomes.

After adjustment for BMI, the primary outcome remained statistically significant among the three groups, consistent with the original analysis. Although minor changes were observed in some secondary outcomes, the overall interpretation and main conclusions of the study were not materially altered.

Based on the re-analysis results, we have revised the relevant tables (Table 3, 4 and 5) and updated the corresponding sections of the manuscript accordingly. (Page 12, Line 255-256; Page 16, Lines 284-286)

**Reference**

1. Fang J, Huang Z, Wang X, Chen L, Cheng K, Deng H, Lin L, Zhao L, Shen X. Comparison of 10.6 μm Laser Moxibustion with Traditional Moxibustion in Knee Osteoarthritic Therapy: A Randomized Noninferiority Clinical Trial. Photobiomodul Photomed Laser Surg. 2021 Jul;39(7):492-498. doi: 10.1089/photob.2021.0030. PMID: 34264766.

2. Wang Q, Lv H, Sun ZT, Tu JF, Feng YW, Wang TQ, Liu CZ. Effect of Electroacupuncture versus Sham Electroacupuncture in Patients with Knee Osteoarthritis: A Pilot Randomized Controlled Trial. Evid Based Complement Alternat Med. 2020 Jul 30;2020:1686952. doi: 10.1155/2020/1686952. PMID: 32802114; PMCID: PMC7414333.

3. Johanson GA, Brooks GP. Initial scale development: sample size for pilot studies. Educ Psychol Meas. 2010; 70: 394–400. doi:10.1177/0013164409355692.

4. Julious SA. Sample size of 12 per group rule of thumb for a pilot study. Pharm Stat. 2005; 4: 287–291. doi:10.1002/pst.185.
