# Supplementary material for: Efficacy and safety of invasive laser acupuncture (650 and 830 nm) on knee osteoarthritis: A pilot randomized clinical trial
Source: PLoS One. 2026 Jul 20;21(7):e0353654. doi: 10.1371/journal.pone.0353654 (PMC13384278; doi:10.1371/journal.pone.0353654)
Supplement: Attachment — Submitted filename: renamed_b7369.docx [file pone.0353654.s011.docx]

**POINT -BY -POINT RESPONSES**

Dear editor and reviewers

We thank you for reviewing our manuscript and for your constructive comments, which have helped improve the manuscript. We have carefully reviewed the comments and have accordingly revised the manuscript. Changes are tracked in the revised manuscript. Below is a detailed, point-by-point response to your comments. We hope that our revisions meet your expectations.

**Additional Editor Comments:**

**My principal concern remains the absence of an appropriate sham control, specifically both a true sham acupuncture group and a sham laser group. In my view, such controls should involve the application of acupuncture (with or without laser) at non-acupoint locations. Without these comparator groups, the current study design does not adequately exclude the possibility that the observed effects are nonspecific and could similarly occur with stimulation at non-acupoint sites.**

**I find this particularly important given that, in the Discussion section, the authors attribute the therapeutic efficacy of both conventional and laser acupuncture to stimulation of defined acupoints. Moreover, the authors’ response to the previous reviewer comment on this issue appears inconsistent with their own interpretation presented in the Discussion. .. please answer this**

**Response:** We apologize for the inconsistency in our previous response

We fully acknowledge the Editor’s concern regarding the absence of a non-acupoint sham control group. As this was an exploratory pilot study, our primary objective was to investigate the wavelength-dependent effects of ILA (650 and 830 nm) compared with those of a sham laser control; therefore, we prioritized these comparators within our limited resources. We agree that without a non-acupoint control, we cannot definitively dismiss non-specific effects.

To address this, we have made the following revisions:

1. We have revised the Abstract and Introduction to more clearly define the specific scope and aims of this pilot study, focusing on the comparative efficacy of the two wavelengths. The revised part is as follows:

This pilot study explored the safety and efficacy of invasive laser acupuncture (ILA) using two wavelengths (650 and 830 nm) compared with those of a sham laser in patients with knee osteoarthritis (KOA). (Page 3, Line 36-38)

Therefore, this exploratory pilot study aimed to explore wavelength-dependent differences in the safety and efficacy of ILA (650 and 830 nm) compared with those of a sham laser control in patients with KOA. (Page 5, Line 84-86)

2. We have added a detailed explanation in the Discussion section (under the fifth limitation) to explicitly acknowledge the lack of a non-acupoint control and the resulting inability to distinguish between site-specific and non-specific responses. The added part is as follows:

Fifth, LLLT application is influenced by a complex combination of parameters, delivery techniques, and irradiation sites [38]. As this exploratory pilot trial was primarily designed to investigate the wavelength-dependent effects of ILA, we prioritized comparing the 650 and 830 nm groups against a sham laser control. Consequently, our study did not incorporate a non-acupoint sham control, limiting our ability to determine whether the observed improvements were attributable to specific acupoints or to non-specific deep-tissue stimulation. Future studies incorporating sham acupoint designs are warranted to clarify the role of site-specificity. (Page 24, Line 427-434)

3. We have revised the Discussion to ensure consistency and to align our interpretation with the actual study design. The added text is as follows:

Effective delivery of energy to deeper structures—such as the synovium, articular cartilage, and muscle, which are pathologic sites in KOA—is important, as more than 90% of the energy from lasers used in musculoskeletal applications is absorbed within the first 10 mm of tissue [35]. (Page 21, Line 371-373)

Although no significant intergroup differences in the WOMAC function subscale and EQ-5D-5L scores were observed at Visits 7 and 13, substantial differences were observed at Visit 14. These findings may have been influenced by the common treatment in all groups, which included teaching of self-management and self-exercise—as strongly recommended in the ACR guidelines for KOA—during the intervention period [3]. (Page 22, Line 389-393)

Review Comments to the Author

**Reviewer #1:**

**Thank you for carefully addressing all the major concerns from my previous review. It is optional that if you want to further strengthen the manuscript, I suggest explicitly reinforcing the necessity of sensitivity analyses for missing data handling in future research, as well as clearly emphasizing the importance of assessing sustained long-term outcomes.:**

**Response:** We would like to thank the reviewer for evaluating our manuscript and for the insightful

To strengthen the manuscript, we have addressed the requirement of sensitivity analyses for missing data handling in future research. Moreover, we have also emphasized the importance of sustained long-term outcomes in the second limitation. The revised text is as follows:

Sixth, missing data were managed using the LOCF method, which is considered suboptimal compared to more robust approaches, such as multiple imputation or mixed-effects modeling [41]. Future studies with more advanced statistical methods are needed to better establish the efficacy of ILA on KOA. (Page 24, Line 434-437)

Second, the follow-up duration in our study was relatively short. Consequently, this study has limitations in assessing sustained efficacy, long-term safety, and potential AEs of ILA. To determine the long-term efficacy and safety of ILA, future studies should incorporate extended follow-up periods such as three to six months or longer. (Page 23, Line 414-417)

**Reviewer #4**:

**After reviewing the revised manuscript and the authors’ responses to the reviewers’ comments, I believe that the authors have satisfactorily addressed the concerns raised in the previous round of review. The revisions have improved the clarity of the manuscript, particularly with regard to methodological explanations, reporting according to relevant guidelines, and discussion of the study limitations.**

**Although the study has inherent limitations associated with its pilot design and relatively small sample size, these issues are now appropriately acknowledged and discussed by the authors. The statistical analyses and presentation of results are generally adequate for an exploratory pilot randomized clinical trial, and the conclusions have been revised to reflect the preliminary nature of the findings.**

**Overall, the manuscript provides useful preliminary evidence regarding the potential efficacy and safety of invasive laser acupuncture for knee osteoarthritis and may serve as a basis for future larger-scale trials.**

**Therefore, I recommend acceptance of the manuscript for publication.**

**Response:** We are deeply grateful for the reviewer’s positive assessment of our manuscript
