# Supplementary material for: Efficacy and safety of invasive laser acupuncture (650 and 830 nm) on knee osteoarthritis: A pilot randomized clinical trial
Source: PLoS One. 2026 Jul 20;21(7):e0353654. doi: 10.1371/journal.pone.0353654 (PMC13384278; doi:10.1371/journal.pone.0353654)
Supplement: Attachment — Submitted filename: Response_to_Reviewer_ILA_KOA_R3_Final.docx [file pone.0353654.s012.docx]

**POINT-BY-POINT RESPONSES**

Dear editor and reviewers

We thank you for reviewing our manuscript and for your constructive comments, which have helped improve the manuscript. We have carefully reviewed the comments and have accordingly revised the manuscript. Changes are tracked in the revised manuscript. Below is a detailed, point-by-point response to your comments. We hope that our revisions meet your expectations.

**Additional Editor Comments:**

**Could you please address the issues raised by the reviewer?**

**Response:**

We sincerely thank the Academic Editor for handling our manuscript across multiple revision rounds. We have carefully addressed each of the points raised by Reviewer #5, including those regarding sample size and statistical power, multiple comparisons, interpretation of findings, randomization and allocation concealment, blinding, clinical relevance, biological plausibility, and the minor comments. Detailed responses to each comment, along with the corresponding revisions to the manuscript, are provided below. We hope that the revised manuscript and our responses adequately address all concerns raised during this review round.

Review Comments to the Author

**Reviewer #1:**

**The authors have sufficiently addressed all of my questions. I do not have any additional concerns or questions.**

**Response:**

We sincerely thank you for the careful review of our manuscript across multiple revision rounds and for confirming that our previous responses have adequately addressed all concerns.

**Reviewer #5**:

**General Assessment**

**This manuscript presents a pilot randomized, placebo-controlled clinical trial evaluating the efficacy and safety of invasive laser acupuncture (ILA) at two wavelengths (650 and 830 nm) in patients with knee osteoarthritis (KOA). The topic is clinically relevant, and the randomized design with a sham control is a strength. However, several methodological and reporting limitations reduce confidence in the robustness and interpretation of the findings. In its current form, the manuscript would benefit from substantial revision.**

**Major Comments**

**Sample Size and Statistical Power**

**The study includes a total of 45 participants (approximately 15 per group). The manuscript does not clearly report an a priori sample size calculation. Given the small sample size, the study is likely underpowered, increasing the risk of both type I and type II errors.**

**Response:**

We sincerely thank the reviewer for this thoughtful comment, and we apologize if our sample size rationale was not sufficiently clear in the original manuscript.

We fully agree that the sample size of the present study is not sufficient to confirm the efficacy of ILA for KOA, and this limitation is acknowledged in the manuscript. For this reason, we adopted an exploratory pilot study design, as explicitly stated in the title, abstract, and main text.

This trial aimed to assess feasibility, estimate preliminary effect sizes, and inform the design of future definitive trials, rather than to provide confirmatory evidence of efficacy. Confirmation of efficacy will require an adequately powered follow-up trial.

An a priori sample size justification is provided in the "Sample size calculation" section. As no prior trials of ILA for KOA were available to inform a conventional power calculation, we followed the established rule-of-thumb for pilot studies, allocating 15 participants per group based on the recommended minimum of 12 per arm [28, 29], with an additional 20% allowance for attrition.

To further clarify the exploratory nature and future direction of this trial, we have revised the following sentence to the "Sample size calculation" section:

We adopted a pilot study design due to the absence of previous studies, limited budgets, and enrollment opportunities. In a three-arm pilot study, the adequate sample size is more than 12 [28, 29]. Assuming that 20% of the participants may drop out, we allocated 15 participants to each group.

Because this study was a pilot study, the sample size was not enough to evaluate the effectiveness and safety of ILA for KOA. However, our pilot study may establish the feasibility of a larger, rigorously designed confirmatory RCT on ILA in patients with KOA and provide preliminary insight into the acceptability and safety of ILA for KOA patients. (Page 14; Line 249-256)

**Multiple Comparisons and Risk of False Positives**

**Multiple outcomes are assessed across several time points without clear indication of correction for multiple comparisons. This raises concern about inflated type I error rates.**

**Response:**

We truly appreciate this important methodological comment. We acknowledge that assessing multiple outcomes across several time points without formal correction for multiple comparisons may inflate the type I error rate.

As an exploratory pilot trial, the aim of the present study was not to provide confirmatory evidence of efficacy, but to assess feasibility, estimate preliminary effect sizes, and inform the design of future definitive trials. Accordingly, the statistically significant findings observed in this study should be interpreted as preliminary and hypothesis-generating rather than confirmatory.

To transparently address this point, we have added the following statement as the seventh limitation.

Seventh, this exploratory pilot trial measured multiple outcomes at several time points without formal adjustment for multiple comparisons. Therefore, the possibility of inflated type I error and false-positive findings should be considered when interpreting the results. The statistically significant findings observed in this study should be regarded as preliminary and hypothesis-generating rather than confirmatory. Future adequately powered confirmatory trials with prespecified hierarchical testing strategies or multiplicity adjustment methods are warranted. (Page 26; Line 474-480)

**Interpretation of Findings**

**As a pilot study, conclusions should be more cautious and emphasize the exploratory nature of the findings rather than suggesting efficacy.**

**Response:**

We fully agree that the conclusions should more clearly reflect the exploratory and preliminary nature of this pilot trial. Accordingly, we have revised the relevant sections to soften the wording and emphasize the exploratory nature of the findings.

Abstract

The 650 and 830 nm ILA exhibited potential short-term improvement in pain reduction. Additionally, partial short-term improvements in pain-related functional disabilities and quality of life were observed. However, these preliminary findings should be confirmed in larger, rigorously designed clinical trials. (Page 3; Line 54-57)

Conclusion

In this exploratory pilot trial, ILA at 650 and 830 nm showed preliminary signals of the potential for a short-term improvement in pain reduction among patients with KOA. Additionally, partial but exploratory short-term improvements were observed in pain-related functional disability and quality of life. However, as we conducted a pilot study with a small sample size and exploratory design, adequately powered, confirmatory trials are warranted to validate these preliminary observations. (Page 26; Line 483-488)

**Randomization and Allocation Concealment**

**The manuscript lacks sufficient detail regarding the randomization process and allocation concealment.**

**Response:**

We agree that the randomization and allocation concealment procedures warranted a more detailed description. Accordingly, we have substantially revised the relevant section in the Methods.

The revised passage is as follows:

The 45 eligible participants were randomly assigned to three groups (control [0 mW power sham laser], 650 [20 mW power, 650 nm wavelength laser], or 830 [20 mW power, 830 nm wavelength laser]) at a 1:1:1 ratio. The randomization sequence was generated by SAS^Ⓡ^ (Version 9.4, SAS Institute, Cary, NC, USA) with block randomization by a statistician who did not participate in the trial. Allocation concealment was ensured through the following procedure. Each allocation code was placed inside an opaque envelope, which was then sealed and labeled with a sequential number. The envelopes were stored in a double-locked cabinet. At the point of enrollment—immediately after a participant had completed baseline measurements and provided informed consent—a designated randomization administrator, who took no part in screening and intervention, outcome assessment, or data analysis, retrieved the next envelope in numerical order, opened it, and recorded the date and signature on a log sheet. The group assignment was disclosed only to the practitioner who delivered the intervention. (Page 10-11; Line 172-183)

**Blinding**

**Further clarification is needed on how blinding was implemented and whether its effectiveness was assessed.**

**Response:**

As per your suggestion, we have expanded the Blinding section in the Methods to provide a more detailed description of how participant and outcome assessor blinding was implemented, and we have explicitly acknowledged that the effectiveness of blinding was not formally assessed using a validated blinding index. This point has also been added to the Limitations section. The revised passages are as follows:

Because of the nature of the ILA treatment, in which the practitioner had to operate the laser device (sham, 650 nm, or 830 nm laser) after inserting the needle, blinding of the practitioner was not feasible. To minimize potential bias, we conducted a patient- and assessor-blinded trial. Participants could not feel any specific sensation distinguishing between the real laser and sham laser, and the device was configured to generate identical operational noise across all groups. In addition, participants wore a blindfold during the procedure to block visual identification of the intervention. Through these methods, participants were unable to distinguish between treatment conditions. The outcome assessor was not in contact with any participant except during the assessment time points. Thus, until trial completion, both participants and assessor were blinded to the group allocation. Formal assessment of blinding effectiveness using a validated blinding index was not performed in this pilot trial. (Page 11; Line 184-194)

Third, owing to the characteristics of ILA, it was not possible to blind the practitioners, as they had to directly operate the laser device. To minimize this risk, we adopted an outcome assessor-blinded design. Nevertheless, the absence of practitioner blinding remains a methodological limitation and may have influenced the trial results. In addition, although we implemented multiple measures to maintain participant and assessor blinding, the effectiveness of blinding was not evaluated using a validated blinding index. Therefore, we could not verify whether the blinding procedures were fully successful. Further research should explore strategies for blinding practitioners when possible, consider the inclusion of independent outcome assessments or more objective outcome measures, and assess blinding effectiveness to further mitigate potential bias. (Page 25; Line 451-460)

**Clinical Relevance**

**The manuscript should address whether observed differences meet minimal clinically important differences (MCID).**

**Response:**

We sincerely thank you for raising this point. The clinical relevance of the within-group changes was previously addressed in the Discussion section using the minimal clinically important improvement (MCII) thresholds for KOA [40]. To further address the reviewer's concern regarding between-group differences (MCID), we have added a detailed analysis in the Discussion based on the MCID values reported in a recent meta-analysis of placebo-controlled trials in KOA. At the primary endpoint (Visit 13), most between-group differences (vs. control) exceeded the MCID thresholds, including VAS during activity and all WOMAC subscales. For VAS at rest, the 830 group difference exceeded the MCID threshold, while the 650 group difference marginally fell below it; this finding has been reported in the revised Discussion. The added passage is as follows:

The responder rates were 26.67% in the control group, 53.33% in the 650 group, and 60% in the 830 group. Although the experimental groups showed numerically higher rates compared to the control group, the difference did not reach statistical significance. This lack of significance may be attributed to the small sample size. Minimal clinically important improvement (MCII), defined as the smallest change in measurement that signifies an important improvement in a patient’s symptom, for KOA is established at -19.9 mm for pain VAS and -9.1 for the WOMAC function subscale [40]. In our study, the 650 group showed reductions of -20.7 mm in VAS at rest, -21.7 mm in VAS during activity, and -14.9 in the WOMAC function subscale. Similarly, the 830 group showed reductions of -22.6 mm in VAS at rest, -22.9 mm in VAS during activity, and -14.9 in the WOMAC function subscale. These improvements observed in both experimental groups exceeded the established MCII thresholds. Despite the lack of a statistically significant difference in responder rates, exceeding MCII thresholds suggested potential clinical relevance. In addition to MCII, minimal clinically important difference (MCID) values for KOA have been reported as VAS 13.7 mm, WOMAC total 6.4, WOMAC pain 1.5, and WOMAC function 4.6 [41]. At the primary endpoint (Visit 13), the between group differences (vs. control) exceeded these MCID thresholds for most outcomes: VAS during activity (15.0 mm, 650 group; 16.2 mm, 830 group), WOMAC total (13.1, 650 group; 11.8, 830 group), WOMAC pain (3.4, 650 group; 2.4, 830 group), and WOMAC function (8.4 for both groups). For VAS at rest, the difference in the 830 group (15.4 mm) exceeded the MCID threshold, while the difference in the 650 group (13.5 mm) closely approached but marginally fell below the threshold. Considering the clinically meaningful differences across most outcomes, these findings also suggested potential clinical relevance. Nevertheless, further well-designed studies with larger sample sizes are necessary to substantiate these findings. (Page 23-24; Line 419-441)

**Biological Plausibility**

**A stronger discussion of the biological mechanism underlying the intervention is recommended.**

**Response:**

We fully agree that a more detailed discussion of the biological mechanism would strengthen the manuscript. Accordingly, we have substantially expanded the relevant passage in the Discussion section to provide a more comprehensive account of photobiomodulation (PBM) mechanisms. The revised passage is as follows:

The reasons behind the reduction in the VAS score and WOMAC total scores are likely attributed to the analgesic and anti-inflammatory effects of photobiomodulation (PBM). PBM exerts several effects on cells by therapeutically exposing cells to low levels of red or near-infrared light. First, in the intracellular compartment, PBM light is thought to modulate cytochrome c oxidase activity in mitochondria, thereby increasing adenosine triphosphate production and modulating intracellular levels of reactive oxygen species. Through this pathway, PBM has been reported to modulate the production of key pro-inflammatory mediators, including prostaglandin E2, interleukin-6, and tumor necrosis factor-alpha. Second, at the cell membrane, PBM may modulate ion channels such as transient receptor potential vanilloid 1, thereby attenuating nociceptor excitability and contributing to analgesia. Third, in the extracellular compartment, PBM may activate latent transforming growth factor-β1 through a redox-dependent activation mechanism, which promotes tissue repair and regeneration. Through these molecular mechanisms acting at multiple cellular compartments, PBM exerts analgesic, anti-inflammatory, and reparative effects, which may contribute to the symptomatic improvement observed in patients with KOA [30-33]. (Page 21-22; Line 376-390)

**Minor Comments**

**Clarify treatment duration wording**

**Response:**

We have revised the treatment duration wording throughout the manuscript to improve clarity. The revised wording is now consistently applied across the Abstract, Methods (Study design), and Methods (Intervention) sections. The revised passage is as follows:

All participants were treated with ILA on one-side of SP9, SP10, ST34, ST35, EX-LE2, EX-LE4, and GB34 for 10 minutes per session, twice weekly, for a total of 12 sessions over 6 weeks. (Page 3; Line 40-42)

The participants were treated with ILA on one-side (more affected side) of SP9 (Yinglingquan; Spleen 9), SP10 (Xuehai; Spleen 10), ST34 (Liangqiu; Stomach 34), ST35 (Dubi; Stomach 35), EX-LE2 (Heding; Extra Lower Extremity 2), EX-LE4 (Neixiyan; Extra Lower Extremity 4), and GB34 (Yanglingquan; Gallbladder 34) for 10 minutes per session, twice weekly, for a total of 12 sessions over 6 weeks. (Page 5-6; Line 100-106)

The participants were treated with ILA on one-side (more affected side) SP9, SP10, ST34, ST35, EX-LE2, EX-LE4, and GB34, which were based on previous randomized controlled trial (RCT) and clinical practice guidelines for KOA [20,21], for 10 minutes per session, twice weekly, for a total of 12 sessions over 6 weeks. (Page 11; Line 197-200)

**Define acupuncture point abbreviations**

**Response:**

We have added explanatory definitions of each abbreviation in the Methods section. The revised passage is as follows:

The participants were treated with ILA on one-side (more affected side) of SP9 (Yinglingquan; Spleen 9), SP10 (Xuehai; Spleen 10), ST34 (Liangqiu; Stomach 34), ST35 (Dubi; Stomach 35), EX-LE2 (Heding; Extra Lower Extremity 2), EX-LE4 (Neixiyan; Extra Lower Extremity 4), and GB34 (Yanglingquan; Gallbladder 34) (Page 5-6; Line 100-105)

**Clarify rescue medication use**

**Response:**

As recommended, we have revised the Methods section to provide clearer details regarding the use of rescue medication. The revised passage is as follows:

Rescue medication (acetaminophen 500 mg, 20 tablets) was provided to all participants at Visit 1 and Visit 13. Participants were instructed to take the medication only when their knee pain was intolerable, at a dosage of 1-2 tablets (500-1,000 mg) per intake at intervals of 4-6 hours, and limited to a maximum of 8 tablets (4,000 mg) per day. (Page 12; Line 212-215)

**Include CONSORT flow diagram**

**Response:**

We would like to respectfully draw the reviewer's attention to the CONSORT 2010 flow diagram, which is provided as Figure 1 and is cited in the Results section (Page 16, Line 286-287). The diagram depicts participant flow through screening, randomization, treatment, and analysis phases, including the number of dropouts in each group.

We hope this clarification adequately addresses the reviewer's concern.

**Minor language edits recommended**

**Response:**

We thank the reviewer for this suggestion. The original manuscript was professionally edited by Editage (Cactus Communications), a well-established academic English-language editing service, prior to the initial submission to ensure linguistic accuracy and clarity. In preparation for the present revision, the manuscript has been professionally re-edited in full by Editage to address grammar, sentence structure, and overall language clarity. We hope these revisions adequately address the reviewer's concern.
