# Supplementary material for: Efficacy and safety of invasive laser acupuncture (650 and 830 nm) on knee osteoarthritis: A pilot randomized clinical trial
Source: PLoS One. 2026 Jul 20;21(7):e0353654. doi: 10.1371/journal.pone.0353654 (PMC13384278; doi:10.1371/journal.pone.0353654)
Supplement: Attachment — Submitted filename: Response_to_Reviewer_ILA_KOA_R4.docx [file pone.0353654.s013.docx]

**POINT-BY-POINT RESPONSES**

Dear editor and reviewers

We thank you for reviewing our manuscript and for your constructive comments, which have helped improve the manuscript. We have carefully reviewed the comments and have accordingly revised the manuscript. Changes are tracked in the revised manuscript. Below is a detailed, point-by-point response to your comments. We hope that our revisions meet your expectations.

**Additional Editor Comments:**

**Could you please address the issues raised by the reviewer?**

**Response:**

We sincerely thank the Academic Editor for handling our manuscript across multiple revision rounds. We have carefully addressed each of the points raised by Reviewer #8, including those regarding sample size and statistical power, multiple comparisons, interpretation of findings, randomization and allocation concealment, blinding, clinical relevance, biological plausibility, and the minor comments. Detailed responses to each comment, along with the corresponding revisions to the manuscript, are provided below. We hope that the revised manuscript and our responses adequately address all concerns raised during this revision.

Review Comments to the Author

**Reviewer #1:**

**The authors have fully addressed each of my comments and concerns, I do not have any other questions.**

**Response:**

We sincerely thank you for the careful review and for confirming that our previous responses have adequately addressed all concerns.

**Reviewer #6**:

**Thank you for the detailed revision and thoughtful point-by-point responses. The manuscript has improved substantially, addressing all comments of reviewers.**

**Response:**

We sincerely thank you for your positive feedback and for recognizing the improvements made to the manuscript.

**Reviewer #8**:

**As an ad-hoc reviewer invited during the third revision, I have focused my evaluation on the remaining scientific soundness, methodological queries, and logical structure of the manuscript.**

**1. Strengths**

**KOA is a major degenerative disorder affecting a significant portion of the population.**

**This pilot trial is well-designed to evaluate the feasibility of two different wavelengths of laser acupuncture, utilizing active acupuncture as a sham laser control for KOA patients.**

**The efficient delivery of laser treatment via acupuncture needles is a notable strength of this study. It suggests a potential for reducing KOA-related pain and improving QoL, thereby contributing valuable insights to the field of KOA treatment.**

**I hope this study, along with further follow-up research on ILA, will offer significant clinical relevance, impact, and a novel integrative approach to KOA management.**

**Throughout the initial and revised manuscripts, the authors have demonstrated transparency and scientific rigor by providing detailed CONSORT and STRICTA-compliant methods, comprehensive supplementary materials (including the study protocol and individual data), and plausible biological mechanisms to ensure the robustness of the trial.**

**Based on my review of the manuscript, I would like to provide some recommendations to further improve the paper.**

**Response:**

We sincerely thank the reviewer for the careful and constructive evaluation of our manuscript, and for recognizing the strengths of this study, including its design, the efficient delivery of laser treatment through acupuncture needles, and the transparency of our reporting. We have carefully addressed the recommendations below.

**2. Weaknesses and Suggestions**

**1) Major Issues**

**The concern regarding the inflation of the Type I error rate due to multiple comparisons of the same outcome across a continuous time series against the baseline remains unaddressed.**

**Although the authors previously responded to a similar point by expanding the limitations section, and this exploratory perspective of this pilot trial is understood and acceptable to some extent, the statistical validity of the findings may still be compromised by this inflated error rate.**

**Given that the study tracks indicators over time but relies solely on independent, point-by-point ANCOVA at each time interval, please consider utilizing a linear mixed-effects model or repeated measures ANCOVA to evaluate the time-by-group interaction effect if a formal re-analysis is feasible.**

**If a full re-analysis is realistically difficult at this stage, the statement of this methodological limitation must be further strengthened. Furthermore, the discussion and conclusion must be rephrased in a conservative tone, explicitly cautioning readers that the individual p-values at each visit were not adjusted for multiple temporal comparisons.**

**Response:**

We thank the reviewer for this important comment. To address the concern regarding inflated type I error from repeated visit-wise comparisons, we performed a supplementary repeated-measures ANCOVA evaluating the time-by-group interaction across visits, which accounts for the longitudinal structure rather than testing each visit independently. In this analysis, the time-by-group interaction remained supportive for the primary VAS outcomes, whereas most secondary outcomes were less robust. Because this analysis was not pre-specified, we retained the original results and presented the repeated-measures ANCOVA as S3 Table. We have also revised the Seventh limitation to acknowledge that the visit-wise p-values were not adjusted for multiple temporal comparisons and to describe the significant findings as preliminary and hypothesis-generating rather than confirmatory. In addition, we have revised the Conclusion in a more conservative tone.

<Manuscript>

A supplementary repeated-measures ANCOVA of outcomes is presented in S3 Table. (Page 20, Line 344)

Seventh, this exploratory pilot trial measured multiple outcomes at several time points, and the individual visit-wise p-values in the main ANCOVA analyses were not adjusted for multiple temporal comparisons, raising the possibility of an inflated type I error. To address this limitation, we additionally performed a supplementary repeated-measures ANCOVA to evaluate the visit-by-group interaction across visits. The results supported longitudinal group differences for the primary VAS outcomes, whereas most secondary outcomes were less robust. As this analysis was not pre-specified in the protocol and the study had a limited sample size, these supplementary findings should be regarded as exploratory. More broadly, the statistically significant findings of the main analyses should therefore be interpreted as preliminary and hypothesis-generating rather than confirmatory, and future adequately powered confirmatory trials with pre-specified longitudinal analyses and multiplicity-control strategies are warranted. (Page 27-28, Line 506-517)

In this exploratory pilot trial, ILA at 650 and 830 nm showed preliminary signals of short-term pain reduction among patients with KOA. However, because this was a pilot study with a small sample size and exploratory design, adequately powered, confirmatory trials are warranted to validate these preliminary observations. (Page 29, Line 535-538)

**While KOA improvement would be generally expected to influence QoL, the actual improvement in QoL achieved through ILA and conventional acupuncture was limited.**

**Please discuss the potential ceiling effect of the EQ-5D-5L in this patient population and the limitations of MCID,**

**and whether EQ-VAS should be considered as complementary outcome to capture more subtle changes for further study?**

**Response:**

We thank the reviewer for this valuable comment. As suggested, we have expanded the Discussion to address the ceiling effect of the EQ-5D-5L, the limitations of established MCID values, and the potential role of the EQ-VAS. Specifically, because our participants had K–L grade 2–3 disease, baseline EQ-5D-5L values were relatively high in all three groups (control, 0.74; 650 nm, 0.75; 830 nm, 0.73), leaving limited room for improvement and raising the possibility of a ceiling effect. We also noted that an established MCID (0.32) was derived only from surgically treated patients, whereas in non-surgical patients the effect sizes were small and no clear MCID could be established; accordingly, established MCID values may not be directly applicable to our non-surgical, high-baseline population. Finally, we noted that incorporating the EQ-VAS as a complementary outcome in future studies may help capture subtle, patient-perceived changes in quality of life.

<Manuscript>

The EQ-5D-5L findings in this trial should be interpreted cautiously. In this K-L grade 2–3 KOA population, baseline EQ-5D-5L scores were relatively high across the three groups (control, 0.74; 650 nm, 0.75; 830 nm, 0.73), leaving limited room for further improvement and raising the possibility of a ceiling effect. A previous study of patients with hip or knee OA established an MCID for improvement of 0.32 in surgically treated patients whose health improved, but among non-surgically managed patients the effect sizes were small and no clear minimal clinically important difference (MCID) exceeding the individual-level minimal detectable change could be established [40]. These findings suggest that EQ-5D-5L MCID estimates vary according to the patient population and treatment context. Accordingly, an established EQ-5D-5L MCID may not be directly applicable to a non-surgical, high-baseline population such as ours, which further limits the interpretation of the modest changes observed. Incorporating a complementary measure such as the EQ-VAS in future studies may help better characterize subtle patient-perceived changes in quality of life. (Page 24-25, Line 435-447)

**The authors provided detailed reporting on AEs with V/S and laboratory results, clarifying that no significant changes were observed among the three groups in their Discussion section and Table S3.**

**However, for more conservative safety evaluation, tracking individual clinical change events (or outlier shifts) is more clinically relevant than merely reporting the intergroup statistical significance of means and p-values of vital signs and lab results.**

**Response:**

We thank the reviewer for this insightful comment. In addition to the existing between-group statistical comparisons (S4A and S4B Tables), we added a description of individual-level shifts (normal at baseline to abnormal at Visit 13) by group (S4C Table). These shifts were minor and not clinically significant, and none were reported as adverse events or considered intervention-related.

<Manuscript>

Detailed changes in the vital signs and laboratory test results, as well as individual-level safety shift, are shown in S4 Table. (Page 21, Line 364-365)

**The purpose and impact of including bilateral KOA patients in a unilateral treatment trial: "treated with ILA on one-side (more affected side)"**

**What was the rationale for including patients with bilateral KOA in this trial? (Please elaborate if this was chosen to enhance external validity (applicability to whole KOA population) or to facilitate favorable enrollment of the study population.)**

**Response:**

We appreciate this valuable observation. The primary rationale was to enhance external validity, as restricting enrollment to unilateral KOA would not reflect the broad KOA population seen in routine clinical practice, where bilateral involvement is common. In addition, as a single-center pilot study with limited trial duration, funding, and recruitment opportunities, not excluding patients with bilateral KOA helped maintain a feasible recruitment pool. Only one pre-specified target knee was treated throughout the trial, and target-knee pain/function outcomes were assessed with reference to this knee.

**Could the main outcomes differ significantly between patients with bilateral KOA and those with unilateral KOA?**

**While treating the more severely affected side, if the severity flipped (i.e., the opposite side became more painful at Visit 7 or Visit 13), could this have influenced the patient-reported outcomes?**

**Response:**

Previous evidence suggests that knee-specific patient-reported pain measures, particularly the WOMAC pain scale, may be affected by crosstalk from co-occurring pain sites, including contralateral knee pain, and that patient-reported physical function in symptomatic KOA may vary according to unilateral versus bilateral knee pain status and contralateral knee pain severity [1,2]. Therefore, the possibility that outcomes may differ between patients with unilateral and bilateral KOA cannot be entirely excluded. In our trial, treatment was confined to a single predefined target knee, and the same target knee was treated throughout the trial. Furthermore, VAS and WOMAC assessments were conducted with reference to that target knee. However, contralateral knee symptoms were not longitudinally recorded; therefore, we could not determine whether a “severity flip” occurred during follow-up. These limitations precluded a formal assessment of whether, and to what extent, symptomatic bilateral involvement may have influenced the outcomes.

**If so, is a subgroup analysis or sensitivity analysis excluding bilateral KOA required?
Response:**

As symptomatic unilateral/bilateral KOA involvement was not systematically recorded as a study variable, and contralateral knee symptoms were not tracked longitudinally, a laterality-based subgroup or sensitivity analysis, including an analysis excluding bilateral KOA, was not feasible. Moreover, given the small sample size of this exploratory pilot study (n = 15 per group), a formal subgroup or sensitivity analysis would have been underpowered and unlikely to yield interpretable results. Accordingly, we have added this issue as a limitation and noted that future confirmatory trials should prospectively record unilateral/bilateral involvement, collect knee-specific outcomes for both knees, and consider stratification or sensitivity analyses based on laterality.

**Patients with bilateral KOA are likely to exhibit different weight-bearing patterns during daily activities and may have a worse prognosis or clinical outcome compared to those with unilateral KOA. Please clarify and discuss these aspects.**

**Response:**

We have added a discussion of the potential biomechanical and prognostic implications of bilateral KOA. Previous gait research in mild-to-moderate KOA reported that between-limb kinematic asymmetry during walking was more prevalent in patients with bilateral symptomatic disease [3], suggesting that bilateral involvement may affect weight-bearing activities. In addition, contralateral knee involvement has been associated with greater structural progression of the ipsilateral knee and a higher subsequent arthroplasty rate [4]. Because unilateral/bilateral KOA status and contralateral symptoms were not systematically recorded in our trial, we could not evaluate these factors directly. We have therefore added this issue as a limitation in the Discussion and noted that future studies should prospectively record laterality, collect knee-specific outcomes for both knees, and consider laterality-based stratification or sensitivity analyses.

Taking the above suggestions together, we have added the following to the limitations section of the manuscript:

Eighth, we did not restrict enrollment to patients with unilateral KOA to reflect real-world clinical practice, while treatment was confined to a single predefined target knee and VAS/WOMAC assessments were conducted for the predefined target knee. However, symptoms in the contralateral knee were not monitored over time. Previous evidence indicates that patient-reported pain and physical function in KOA may be influenced by contralateral knee pain and by unilateral versus bilateral symptom status [44,45]. We therefore could not determine whether the relative severity between the two knees shifted during follow-up, and an influence of the untreated contralateral knee on the patient-reported outcomes cannot be excluded. Prior work also suggests that bilateral involvement may matter clinically: between-limb gait asymmetry has been reported to be more common in bilateral than in unilateral mild-to-moderate KOA [46], and coexisting contralateral KOA has been linked to faster structural progression and a higher subsequent arthroplasty rate in the index knee [47]. Because the symptomatic unilateral/bilateral KOA status was not recorded as a study variable, and given the small per-group sample of this exploratory pilot trial, a laterality-based subgroup or sensitivity analysis was not feasible. Future confirmatory trials should prospectively document unilateral versus bilateral involvement, collect outcomes for both knees, and consider stratified or sensitivity analyses based on laterality. (Page 28, Line 517-533)

**If statistical significance between visits occurred at Visit 13 but not at Visit 7, does this imply that LLLT has a delayed or cumulative effect, whereas conventional acupuncture plays a major role during the early phase?**

**How would this timeline-related result be explained, in the background of a dose-response effect or the multiple exposures to LLLT and acupuncture treatment?**

**Response:**

We thank the reviewer for this insightful comment. We have revised the Discussion to interpret the temporal pattern of the VAS findings more cautiously. The clinical effects of laser therapy are influenced by multiple parameters, including not only wavelength and per-session dose but also irradiation time, treatment frequency, and total treatment duration [5]. Because the per-session laser parameters were fixed within each active group, we cautiously interpreted the observed time course as reflecting the temporal pattern of response under a fixed treatment regimen rather than evidence of a formal dose-response or cumulative exposure-response relationship of laser therapy.

<Manuscript>

For the VAS outcomes, nominal between-group differences were not observed at Visit 7 but were observed at Visit 13 and were sustained at Visit 14. Although this time course might suggest a gradual response after repeated ILA sessions, the exploratory design and small sample size preclude any firm interpretation of this temporal pattern. The observation should therefore be regarded as a preliminary observation rather than evidence of a specific temporal effect. (Page 24, Line 425-429)

**Furthermore, it is intriguing that 12 sessions of active acupuncture resulted in only a -7 mm difference on the VAS, which falls below the MCID threshold.**

**Response:**

We agree that the change in the control arm was modest and below commonly used MCID thresholds. The therapeutic effect of acupuncture is influenced by multiple specific and non-specific factors, including needling sensation (de-qi), manipulation, and needle retention time [6]. In this trial, the acupuncture procedure was not configured to optimize these factors, because the study was designed to compare the wavelength-dependent effects of ILA. The control group response may therefore be interpreted within the procedural context of this trial, in which the needling conditions were not optimized, unlike in conventional acupuncture trials.

**While the sham control is well-designed and rigorous, there was an unavoidable lack of practitioner blinding, and this functioned as an active control.**

**Please discuss the potential bias arising from the practitioners' intentions or expectations when administering the control acupuncture treatment.**

**Response:**

We agree that the unavoidable lack of practitioner blinding is an important methodological consideration. Because the control group received needle insertion with sham laser output at 0 mW and practitioners had to operate the laser device according to group allocation, practitioners were necessarily aware of group allocation. Their intentions or expectations while administering the control treatment may have introduced performance bias. We have strengthened this point in the limitations section of the Discussion.

<Manuscript>

Third, owing to the characteristics of ILA, it was not possible to blind the practitioners, as they had to directly operate the laser device. To minimize this risk, we adopted a patient- and outcome assessor-blinded design. Nevertheless, because practitioners were aware of group allocation, their awareness and any associated intentions or expectations while administering the control treatment may have introduced performance bias across the treatment and control groups. The absence of practitioner blinding therefore remains a methodological limitation and may have influenced the trial results. In addition, although we implemented multiple measures to maintain participant and assessor blinding, the effectiveness of blinding was not evaluated using a validated blinding index. Therefore, we could not verify whether the blinding procedures were fully successful. Further research should explore strategies for blinding practitioners when possible, consider the inclusion of independent outcome assessments or more objective outcome measures, and assess blinding effectiveness to further mitigate potential bias. (Page 26-27, Line 480-492)

**In the final section of the Results: "830 nm ILA group showed a larger mean change in the primary outcome compared with the 650 nm group."**

**If this pilot trial was aimed at determining superiority between the 830 nm and 650 nm wavelengths, a three-group ANCOVA would not directly compare the head-to-head difference between these two active groups.**

**I suggest that this statement be rephrased in a more conservative tone, as it is currently based strictly on mean differences without direct intergroup statistical significance.**

**Response:**

We appreciate the reviewer's thoughtful recommendation. We agree that the statement should be more conservative, as this trial was not designed to test superiority between the two wavelengths. We have revised the statement accordingly.

<Manuscript>

Although we did not directly compare the superiority of the 830 nm and 650 nm wavelengths, we calculated the sample size for a larger future study focused on the 830 nm ILA based on the mean change observed in this group. (Page 21, Line 368-370)

**2) Minor Considerations**

**Please consider providing a more detailed visual representation of the major outcomes would be beneficial for readers (e.g. a raincloud plot) rather than presenting them solely in the current data matrix format.**

**Response:**

Thank you for your advice. We have added the raincloud plot for primary outcome as Fig. 2.

**This pilot study introduces the intervention as a specific type of LLLT in the background section. However, since the control group was designed under the premise that active acupuncture is already effective for KOA (without an inactive/placebo control), the authors should provide the underlying evidence for acupuncture's efficacy in the Introduction (or discussion) so that readers can fully understand the rationale behind this control setting.**

**Response:**

Thank you for flagging this. As suggested, we have noted in the Introduction that acupuncture has been conditionally recommended as a treatment modality for KOA in the ACR clinical practice guideline, and it helps readers understand the rationale behind the control setting in this trial.

<Manuscript>

Invasive laser acupuncture (ILA) combines acupuncture—a modality conditionally recommended for KOA in the ACR guideline [3]—with low-level laser therapy. It is performed by invasive irradiation and acupuncture stimulation using an optical fiber-coupled laser diode (InGaAlP 650 nm; GaAlAs 830 nm) inserted inside the needle (material, stainless steel; length, 30 mm; inner diameter, 0.15 mm; external diameter, 0.3 mm). (Page 4-5, Line 78-82)

**Please ensure the phrase "VAS during activity" is defined clearly for the readers. Does it refer to walking under weight-bearing conditions or the active range of motion of the joint? "Activity" can vary widely in terms of tasks and intensity.**

**Response:**

We are grateful for this helpful pointer. We added the definition of VAS during activity used in this trial.

The primary outcome was the difference in changes in the VAS scores at rest and during activity (while walking on flat ground) among the three groups at visit 13. (Page 13, Line 223-224)

**p.14, line 254: Please change "effectiveness" to "efficacy".**

**Response:**

Thank you for pointing this out. We corrected the term accordingly.

<Manuscript>

Because this study was a pilot study, the sample size was not enough to evaluate the efficacy and safety of ILA for KOA. (Page 14, Line 254-255)

**Table 1: Please review the table, as it contains two duplicated rows for the VAS. Additionally, VAS data and vital sign surveillance would be more appropriately placed in the assessment/safety section.**

**Response:**

Thank you for your advice. We have removed the duplicated VAS rows from Table 1 and relocated the VAS and vital sign data to the assessment section, as suggested.

**Table 2: Please change the "Dependent" column header to "Dependent variable".
Response:**

We thank the reviewer for pointing out this oversight. The column header was originally labeled "Dependent variable," but the full text was not displayed owing to the table resizing process. We have increased the row height so that the complete header is now visible.

**References**

1. Riddle DL, Perera RA. The WOMAC Pain Scale and Crosstalk From Co-occurring Pain Sites in People With Knee Pain: A Causal Modeling Study. Phys Ther. 2020 Sep 28;100(10):1872-1881. doi: 10.1093/ptj/pzaa098.

2. Riddle DL, Stratford PW. Unilateral vs bilateral symptomatic knee osteoarthritis: associations between pain intensity and function. Rheumatology (Oxford). 2013 Dec;52(12):2229-37. doi: 10.1093/rheumatology/ket291.

3. Mills K, Hettinga BA, Pohl MB, Ferber R. Between-limb kinematic asymmetry during gait in unilateral and bilateral mild to moderate knee osteoarthritis. Arch Phys Med Rehabil. 2013 Nov;94(11):2241-7. doi: 10.1016/j.apmr.2013.05.010. Epub 2013 Jun 5. PMID: 23747645.

4. Dai Z, Yang T, Liu J. Contralateral knee osteoarthritis is a risk factor for ipsilateral knee osteoarthritis progressing: a case control study. BMC Musculoskelet Disord. 2024 Mar 2;25(1):190. doi: 10.1186/s12891-024-07292-6. PMID: 38431551; PMCID: PMC10908155.

5. Huang Z, Ma J, Chen J, Shen B, Pei F, Kraus VB. The effectiveness of low-level laser therapy for nonspecific chronic low back pain: a systematic review and meta-analysis. Arthritis Res Ther. 2015;17:360. doi:10.1186/s13075-015-0882-0

6. Shi GX, Yang XM, Liu CZ, Wang LP. Factors contributing to therapeutic effects evaluated in acupuncture clinical trials. Trials. 2012;13:42. doi:10.1186/1745-6215-13-42
