# Supplementary material for: Efficacy and safety of invasive laser acupuncture (650 and 830 nm) on knee osteoarthritis: A pilot randomized clinical trial
Source: PLoS One. 2026 Jul 20;21(7):e0353654. doi: 10.1371/journal.pone.0353654 (PMC13384278; doi:10.1371/journal.pone.0353654)
Supplement: Peer review history

# Peer review: Decision Letter 0

**DOI:** 10.1371/journal.pone.0353654.r001

**Authors:** Ramada Rateb Khasawneh

13 Oct 2025

PONE-D-25-31350

Efficacy and safety of invasive laser acupuncture (650 and 830 nm) on knee osteoarthritis: A pilot randomized clinical trial

PLOS ONE

Dear Dr. Kim,

Thank you for submitting your manuscript to PLOS ONE. After careful consideration, we feel that it has merit but does not fully meet PLOS ONE’s publication criteria as it currently stands. Therefore, we invite you to submit a revised version of the manuscript that addresses the points raised during the review process.

==============================

Can you please answer the following

Control design: The study lacks a non-acupoint laser control group, making it difficult to determine whether effects are acupoint-specific or due to nonspecific laser stimulation.

BMI imbalance: Significant baseline BMI differences (p = 0.0072) were not adjusted for; this could confound the outcomes.

Sample size: The small sample (n = 45) limits statistical power and generalizability; findings should be interpreted as preliminary.

Missing data handling: Use of LOCF is suboptimal; more robust methods such as multiple imputation or mixed-effects modeling are recommended.

Interpretation of results: The conclusions appear overstated given the pilot design; results should be described more cautiously as exploratory.

thank you

==============================

Please submit your revised manuscript by Nov 27 2025 11:59PM. If you will need more time than this to complete your revisions, please reply to this message or contact the journal office at plosone@plos.org. When you're ready to submit your revision, log on to https://www.editorialmanager.com/pone/ and select the 'Submissions Needing Revision' folder to locate your manuscript file.

Please include the following items when submitting your revised manuscript:

- A rebuttal letter that responds to each point raised by the academic editor and reviewer(s). You should upload this letter as a separate file labeled 'Response to Reviewers'.
- A marked-up copy of your manuscript that highlights changes made to the original version. You should upload this as a separate file labeled 'Revised Manuscript with Track Changes'.
- An unmarked version of your revised paper without tracked changes. You should upload this as a separate file labeled 'Manuscript'.

-->If you would like to make changes to your financial disclosure, please include your updated statement in your cover letter. Guidelines for resubmitting your figure files are available below the reviewer comments at the end of this letter.

If applicable, we recommend that you deposit your laboratory protocols in protocols.io to enhance the reproducibility of your results. Protocols.io assigns your protocol its own identifier (DOI) so that it can be cited independently in the future. For instructions see: https://journals.plos.org/plosone/s/submission-guidelines#loc-laboratory-protocols. Additionally, PLOS ONE offers an option for publishing peer-reviewed Lab Protocol articles, which describe protocols hosted on protocols.io. Read more information on sharing protocols at https://plos.org/protocols?utm_medium=editorial-email&utm_source=authorletters&utm_campaign=protocols.

We look forward to receiving your revised manuscript.

Kind regards,

Ramada Rateb Khasawneh

Academic Editor

PLOS ONE

Journal requirements:

When submitting your revision, we need you to address these additional requirements.

1. Please ensure that your manuscript meets PLOS ONE's style requirements, including those for file naming. The PLOS ONE style templates can be found at

https://journals.plos.org/plosone/s/file?id=wjVg/PLOSOne_formatting_sample_main_body.pdf and

https://journals.plos.org/plosone/s/file?id=ba62/PLOSOne_formatting_sample_title_authors_affiliations.pdf

2. We note that you have selected “Clinical Trial” as your article type. PLOS ONE requires that all clinical trials are registered in an appropriate registry (the WHO list of approved registries is at

https://www.who.int/clinical-trials-registry-platform/network/primary-registries" https://www.who.int/clinical-trials-registry-platform/network/primary-registriesand more information on trial registration is at

http://www.icmje.org/about-icmje/faqs/clinical-trials-registration/). Please state the name of the registry and the registration number (e.g. ISRCTN or ClinicalTrials.gov) in the submission data and on the title page of your manuscript. a) Please provide the complete date range for participant

recruitment and follow-up in the methods section of your manuscript. b) If you have not yet registered your trial in an appropriate registry, we now require you to do so and will need confirmation of the trial registry number before we can pass your paper to the next stage of review. Please include in the Methods section of your paper your reasons for not registering this study before enrolment of participants started. Please confirm that all related trials are registered by stating: “The authors confirm that all ongoing and related trials for this drug/intervention are registered”. Please see http://journals.plos.org/plosone/s/submission-guidelines#loc-clinical-trials for our policies on clinical trials.

3. Thank you for stating the following financial disclosure:

[This work was supported by a grant (RS-2023-KH139215) from the Korea Health Technology R&D Project through the Korea Health Industry Development Institute (KHIDI), funded by the Ministry of Health and Welfare, Republic of Korea.].

Please state what role the funders took in the study.  If the funders had no role, please state: "The funders had no role in study design, data collection and analysis, decision to publish, or preparation of the manuscript."

If this statement is not correct you must amend it as needed.

Please include this amended Role of Funder statement in your cover letter; we will change the online submission form on your behalf.

4. Your ethics statement should only appear in the Methods section of your manuscript. If your ethics statement is written in any section besides the Methods, please move it to the Methods section and delete it from any other section. Please ensure that your ethics statement is included in your manuscript, as the ethics statement entered into the online submission form will not be published alongside your manuscript.

5. If the reviewer comments include a recommendation to cite specific previously published works, please review and evaluate these publications to determine whether they are relevant and should be cited. There is no requirement to cite these works unless the editor has indicated otherwise.

6. Please review your reference list to ensure that it is complete and correct. If you have cited papers that have been retracted, please include the rationale for doing so in the manuscript text, or remove these references and replace them with relevant current references. Any changes to the reference list should be mentioned in the rebuttal letter that accompanies your revised manuscript. If you need to cite a retracted article, indicate the article’s retracted status in the References list and also include a citation and full reference for the retraction notice.

Additional Editor Comments:

Can you please answer the following

Control design: The study lacks a non-acupoint laser control group, making it difficult to determine whether effects are acupoint-specific or due to nonspecific laser stimulation.

BMI imbalance: Significant baseline BMI differences (p = 0.0072) were not adjusted for; this could confound the outcomes.

Sample size: The small sample (n = 45) limits statistical power and generalizability; findings should be interpreted as preliminary.

Missing data handling: Use of LOCF is suboptimal; more robust methods such as multiple imputation or mixed-effects modeling are recommended.

Interpretation of results: The conclusions appear overstated given the pilot design; results should be described more cautiously as exploratory.

thank you

[Note: HTML markup is below. Please do not edit.]

Reviewers' comments:

Reviewer's Responses to Questions

**Comments to the Author**

1. Is the manuscript technically sound, and do the data support the conclusions?

The manuscript must describe a technically sound piece of scientific research with data that supports the conclusions. Experiments must have been conducted rigorously, with appropriate controls, replication, and sample sizes. The conclusions must be drawn appropriately based on the data presented.

Reviewer #1: Yes

Reviewer #2: Partly

Reviewer #3: Yes

**********

2. Has the statistical analysis been performed appropriately and rigorously?

Reviewer #1: Yes

Reviewer #2: Yes

Reviewer #3: Yes

**********

3. Have the authors made all data underlying the findings in their manuscript fully available?

The PLOS Data policy requires authors to make all data underlying the findings described in their manuscript fully available without restriction, with rare exception (please refer to the Data Availability Statement in the manuscript PDF file). The data should be provided as part of the manuscript or its supporting information, or deposited to a public repository. For example, in addition to summary statistics, the data points behind means, medians and variance measures should be available. If there are restrictions on publicly sharing data—e.g. participant privacy or use of data from a third party—those must be specified.

Reviewer #1: Yes

Reviewer #2: Yes

Reviewer #3: Yes

**********

4. Is the manuscript presented in an intelligible fashion and written in standard English?

PLOS ONE does not copyedit accepted manuscripts, so the language in submitted articles must be clear, correct, and unambiguous. Any typographical or grammatical errors should be corrected at revision, so please note any specific errors here.

Reviewer #1: Yes

Reviewer #2: No

Reviewer #3: Yes

**********

5. Review Comments to the Author

Please use the space provided to explain your answers to the questions above. You may also include additional comments for the author, including concerns about dual publication, research ethics, or publication ethics. (Please upload your review as an attachment if it exceeds 20,000 characters)

Reviewer #1: This pilot randomized clinical trial investigates the efficacy and safety of invasive laser acupuncture (ILA) at wavelengths of 650 nm and 830 nm compared to a sham control in patients with knee osteoarthritis (KOA). The study employs a robust design, adhering closely to CONSORT and SPIRIT guidelines, including proper randomization and effective blinding of patients and outcome assessors. Clinically relevant outcomes such as pain (VAS), functional impairment (WOMAC), and quality of life (EQ-5D-5L) were assessed using validated tools. Findings indicate significant short-term improvements in pain relief, functional outcomes, and quality of life with ILA treatment, and no substantial adverse events were reported. However, I have the following concerns:

1. The small sample size (n=45) is adequate for a pilot study but insufficient for drawing definitive conclusions regarding efficacy and safety. I would recommend explicitly acknowledging this limitation in the manuscript, providing clear justification for the chosen sample size, and including preliminary sample-size estimations for future large-scale studies based on the variability and effect sizes observed.

2. The use of the last observation carried forward (LOCF) method for handling missing data may introduce bias and potentially affect the validity of findings. I suggest employing multiple imputation or conducting sensitivity analyses to examine the robustness of the results to different methods of missing-data handling, thereby strengthening the credibility of your conclusions.

3. Due to the intervention’s nature, investigators delivering the treatment were not blinded, potentially introducing performance bias. I recommend clearly discussing this limitation and proposing strategies, such as independent outcome assessments or objective outcome measures, to mitigate bias in future trials.

4. There was a significant baseline imbalance in body mass index (BMI) among groups (p=0.0072), which could confound the outcomes related to pain and function. I suggest explicitly addressing this issue in the manuscript and performing analyses adjusted for BMI to account for possible confounding effects.

5. Although significant differences were observed in primary outcomes (VAS and WOMAC), the responder rate defined by OMERACT-OARSI criteria was not significantly different among groups, raising questions regarding clinical significance. I recommend clearly interpreting these results in the context of minimal clinically important differences (MCID) to enhance clinical relevance and interpretability.

6. The short follow-up period (6 weeks post-intervention) limits assessment of the sustained effects of ILA treatment. I suggest clearly addressing this limitation and recommending longer follow-up durations (3–6 months or longer) in future studies to determine long-term treatment effects.

7. The evaluation of safety data is limited due to the study’s small sample size and short duration. I recommend explicitly noting this limitation in the manuscript and advising larger-scale trials with extended follow-up periods to robustly assess treatment safety and potential adverse events.

8. This was a single-center study, limiting the generalizability of the results. I suggest clearly discussing this limitation and recommending that future research involve multicenter studies with more diverse patient populations to improve external validity and applicability of the findings.

Reviewer #2: To meet STRICTA Guidelines:

1. Identify depth of insertion

2. Name as well as number acupuncture points

3. Description of participating acupuncturists (qualification or professional affiliation, years in acupuncture practice, other relevant experience)

Language/clarity issues:

Line 67: Define invasive laser acupuncture – in intro – needle size, diode used, etc.

Line 127 "daily mean pain visual analogue scale [VAS] score during activity and at rest over the past week up to screening is between 35 and 74." VAS is usually a score of 0-10. If you are using a 0-100 VAS, this needs to be explained here.

Line 158 assigned to three groups (control, 650, or 830) – need to add nm not just the number. And "control" should also be expressed as "0"

Line 165: During the period of this trial, the assessor did not approach any participant except the

assessment time – this is unclear. What is meant by approach? Interact? Speak? See?

258: 45 participants survived and... SURVIVED is the wrong word - perhaps replace with "were accepted as trial participants"

Line 329: asthenia – are you using this to indicate feeling faintness? Was this during treatment?

In your discussion, you suggest significant outcomes. Then in your Sample Size Calculation, you correctly note that sample size was too limited to evaluate effectiveness. be clearer about the issue with sample size in the discussion.

Reviewer #3: 1. The text indicates that rescue medications were administered during Visit 1 and Visit 13. Were these provided to all participants, or exclusively to those exhibiting severe symptoms? If limited to a subset of participants, could this have influenced the observed outcomes?

2. This study employed a relatively small sample size. Is the comparison involving 15 cases statistically significant? Please provide supporting references or statistical justification for this finding.

3. Differences in body mass index exist across the three groups. Given that clinical practice widely acknowledges an association between body weight and KOA, were potential confounding effects related to BMI adjusted for in the analysis? Additionally, can the robustness of the statistical methods employed be confirmed?

**********

6. PLOS authors have the option to publish the peer review history of their article (what does this mean?). If published, this will include your full peer review and any attached files.

If you choose “no”, your identity will remain anonymous but your review may still be made public.

**Do you want your identity to be public for this peer review?**  For information about this choice, including consent withdrawal, please see our Privacy Policy.

Reviewer #1: No

Reviewer #2: **Yes:** Jennifer Brett

Reviewer #3: No

**********

[NOTE: If reviewer comments were submitted as an attachment file, they will be attached to this email and accessible via the submission site. Please log into your account, locate the manuscript record, and check for the action link "View Attachments". If this link does not appear, there are no attachment files.]

While revising your submission, please upload your figure files to the Preflight Analysis and Conversion Engine (PACE) digital diagnostic tool, https://pacev2.apexcovantage.com/. PACE helps ensure that figures meet PLOS requirements. To use PACE, you must first register as a user. Registration is free. Then, login and navigate to the UPLOAD tab, where you will find detailed instructions on how to use the tool. If you encounter any issues or have any questions when using PACE, please email PLOS at figures@plos.org. Please note that Supporting Information files do not need this step.

# Author comment: Author response to Decision Letter 1

**DOI:** 10.1371/journal.pone.0353654.r002

5 Mar 2026

Additional Editor Comments:

Control design: The study lacks a non-acupoint laser control group, making it difficult to determine whether effects are acupoint-specific or due to nonspecific laser stimulation.

Response: We apologize for the confusion

The objective of our study is to explore the effects of non-specific invasive laser stimulation (650 nm and 830 nm) on KOA, rather than focusing on acupoint-specific effects. Therefore, all three groups received acupuncture at the same acupoints. Following this, the control group received a sham laser, while the experimental groups received 650 nm and 830 nm laser stimulation, respectively. (Page 10, Line 166-168)

BMI imbalance: Significant baseline BMI differences (p = 0.0072) were not adjusted for; this could confound the outcomes.

Response: We appreciate your insightful comment regarding the baseline BMI difference

In accordance with the suggestion, we have re-analyzed our data using Analysis of Covariance (ANCOVA), with BMI included as a covariate for all primary and secondary outcomes.

After adjustment for BMI, the primary outcome remained statistically significant among the three groups, consistent with the original analysis. Although minor changes were observed in some secondary outcomes, the overall interpretation and main conclusions of the study were not materially altered.

Based on the re-analysis results, we have revised the relevant tables (Table 3, 4 and 5) and updated the corresponding sections of the manuscript accordingly. (Page 12, Line 255-256; Page 16, Lines 284-286)

Sample size: The small sample (n = 45) limits statistical power and generalizability; findings should be interpreted as preliminary.

Response: We thank you for pointing this out

We have explicitly acknowledged this limitation in the revised manuscript. Furthermore, we have strengthened our conclusion (Page 24, Line 429-433) to emphasize that "well-designed studies with larger sample sizes are required to validate these preliminary findings."

(Page 22, Line 407-413)

First, we conducted only a pilot single-center study owing to the limitations of trial duration, research funding, and opportunities for recruitment. Therefore, the sample size and diversity were insufficient to fully validate the efficacy and safety of ILA in the treatment of KOA. These factors may have introduced bias into the trial results. In future studies, multi-center trials with larger sample sizes should be conducted to encompass a more diverse patient population, thereby enhancing the external validity and clinical applicability of the results.

(Page 24, Line 429-433)

The ILA at 650 and 830 nm suggested the potential of short-term improvement in pain reduction among patients with KOA. Additionally, partial but exploratory short-term improvements were observed in pain-related functional disability and quality of life. However, as we conducted a pilot study with a small sample size, further well–designed studies with larger sample are required to validate these preliminary findings.

Missing data handling: Use of LOCF is suboptimal; more robust methods such as multiple imputation or mixed-effects modeling are recommended.

Response: We appreciate the reviewer’s valuable methodological suggestion

In this study, we employed the Last Observation Carried Forward (LOCF) method because it has been utilized in previous clinical trials investigating the effects of laser therapy or acupuncture for KOA [1,2]. Our study protocol was originally designed and approved by the Institutional Review Board (IRB) based on the LOCF approach, following these established precedents.

While we adhered to the pre-specified protocol for the primary analysis, we acknowledge that LOCF is not the most robust method for handling missing data. Accordingly, we will adopt more advanced statistical methods, such as multiple imputation or mixed-effects models, in the design of our future large-scale trials.

Interpretation of results: The conclusions appear overstated given the pilot design; results should be described more cautiously as exploratory.

Response: We completely agree with your feedback.

We have revised the conclusion to use more conservative and exploratory language, as suggested. We have also explicitly stated that these preliminary findings should be validated with larger sample size.

(Page 24, Line 429-433)

The ILA at 650 and 830 nm suggested the potential of short-term improvement in pain reduction among patients with KOA. Additionally, partial but exploratory short-term improvements were observed in pain-related functional disability and quality of life. However, as we conducted a pilot study with a small sample size, further well–designed studies with larger sample are required to validate these preliminary findings.

Reviewer #1: This pilot randomized clinical trial investigates the efficacy and safety of invasive laser acupuncture (ILA) at wavelengths of 650 nm and 830 nm compared to a sham control in patients with knee osteoarthritis (KOA). The study employs a robust design, adhering closely to CONSORT and SPIRIT guidelines, including proper randomization and effective blinding of patients and outcome assessors. Clinically relevant outcomes such as pain (VAS), functional impairment (WOMAC), and quality of life (EQ-5D-5L) were assessed using validated tools. Findings indicate significant short-term improvements in pain relief, functional outcomes, and quality of life with ILA treatment, and no substantial adverse events were reported. However, I have the following concerns:

1. The small sample size (n=45) is adequate for a pilot study but insufficient for drawing definitive conclusions regarding efficacy and safety. I would recommend explicitly acknowledging this limitation in the manuscript, providing clear justification for the chosen sample size, and including preliminary sample-size estimations for future large-scale studies based on the variability and effect sizes observed.

Response: We fully agree with the point you have raised.

We have explicitly stated the justification for the chosen sample size and acknowledged that it is insufficient to fully evaluate the efficacy and safety of the intervention (Page 13, Line 233-239).

And, we have added a new subsection titled "Preliminary sample size estimation for future studies." In this section, we provided a calculated sample size for future large-scale trials based on the effect sizes and variability observed in the current study.

(Page 20; Line 339-347)

Preliminary sample size estimation for future studies

Following our pilot trial, in which the 830 nm ILA group showed a larger mean change in the primary outcome compared with the 650 nm group, we calculated the sample size for a future larger study focused on the 830 nm ILA. Based on the differences in VAS score changes observed in the pilot trial, we conservatively set the mean difference (standard deviation) between the control and 830 nm groups at 12 (17) for the sample size estimation. With a 1:1 allocation ratio, 90% power, and a two-tailed significance level of 5%, 43 participants per group (86 in total) would be required. Considering the 15% dropout rate observed in the pilot trial, we estimated that 102 participants (51 per group) would need to be recruited.

2. The use of the last observation carried forward (LOCF) method for handling missing data may introduce bias and potentially affect the validity of findings. I suggest employing multiple imputation or conducting sensitivity analyses to examine the robustness of the results to different methods of missing-data handling, thereby strengthening the credibility of your conclusions.

Response: We appreciate the reviewer’s valuable methodological suggestion

In this study, we employed the Last Observation Carried Forward (LOCF) method because it has been utilized in previous clinical trials investigating the effects of laser therapy or acupuncture for KOA [1,2]. Our study protocol was originally designed and approved by the Institutional Review Board (IRB) based on the LOCF approach, following these established precedents.

While we adhered to the pre-specified protocol for the primary analysis, we acknowledge that LOCF is not the most robust method for handling missing data. Accordingly, we will adopt more advanced statistical methods, such as multiple imputation or mixed-effects models, in the design of our future large-scale trials.

3. Due to the intervention’s nature, investigators delivering the treatment were not blinded, potentially introducing performance bias. I recommend clearly discussing this limitation and proposing strategies, such as independent outcome assessments or objective outcome measures, to mitigate bias in future trials.

Response: We sincerely thank you for your constructive feedback regarding performance bias.

As suggested, we have revised the limitation section (in the discussion) to more clearly acknowledge the difficulty of blinding practitioners due to the nature of the ILA intervention.

To mitigate this potential bias, we adopted an outcome assessor-blinded design, ensuring that outcome evaluations were conducted by assessors who were not involved in the treatment delivery. We have also clarified in the Discussion that future trials should explore strategies to blind practitioners where feasible and consider incorporating independent outcome assessments or more objective outcome measures to further reduce potential bias.

(Page 23, Line 416-422)

Third, owing to the characteristics of ILA, it was not possible to blind the practitioners, as they had to directly operate the laser device. To minimize this risk, we adopted an outcome assessor-blinded design. Nevertheless, the absence of practitioner blinding remains a methodological limitation and may have influenced the trial results. Future studies should explore strategies to blind practitioners when possible and consider the inclusion of independent outcome assessments or more objective outcome measures to further mitigate potential bias.

4. There was a significant baseline imbalance in body mass index (BMI) among groups (p=0.0072), which could confound the outcomes related to pain and function. I suggest explicitly addressing this issue in the manuscript and performing analyses adjusted for BMI to account for possible confounding effects.

Response: We appreciate your insightful comment regarding the baseline BMI difference

In accordance with the suggestion, we have re-analyzed our data using Analysis of Covariance (ANCOVA), with BMI included as a covariate for all primary and secondary outcomes.

After adjustment for BMI, the primary outcome remained statistically significant among the three groups, consistent with the original analysis. Although minor changes were observed in some secondary outcomes, the overall interpretation and main conclusions of the study were not materially altered.

Based on the re-analysis results, we have revised the relevant tables (Table 3, 4 and 5) and updated the corresponding sections of the manuscript accordingly. (Page 12, Line 255-256; Page 16, Lines 284-286)

5. Although significant differences were observed in primary outcomes (VAS and WOMAC), the responder rate defined by OMERACT-OARSI criteria was not significantly different among groups, raising questions regarding clinical significance. I recommend clearly interpreting these results in the context of minimal clinically important differences (MCID) to enhance clinical relevance and interpretability.

Response: We agree that the recommended addition/change significantly strengthens the manuscript.

As suggested, we have expanded the Discussion section to interpret our results using the Minimal Clinically Important Improvement (MCII). We have clarified that despite the non-significant responder rate, the improvement in primary outcomes (VAS and WOMAC pain subscale) surpassed the MCII thresholds. These findings suggest that ILA treatment may offer clinically meaningful improvements for patients with KOA, even if those improvements do not meet the OMERACT-OARSI responder criteria.

(Page 22-23; Line 393-406)

The responder rates were 26.67% in the control group, 53.33% in the 650 group, and 60% in the 830 group. Although the experimental groups showed numerically higher rates compared to the control group, the difference did not reach statistical significance. This lack of significance may be attributed to the small sample size. Minimal clinically important improvement (MCII), defined as the smallest change in measurement that signifies an important improvement in a patient’s symptom, for KOA is established at -19.9 mm for pain VAS and -9.1 for the WOMAC function subscale [40]. In our study, the 650 group showed reductions of -20.7 mm in VAS at rest, -21.7 mm in VAS during activity, and -14.9 in the WOMAC function subscale. Similarly, the 830 group showed reductions of -22.6 mm in VAS at rest, -22.9 mm in VAS during activity, and -14.9 in the WOMAC function subscale. These improvements observed in both experimental groups exceeded the established MCII thresholds. Despite the lack of a statistically significant difference in responder rates, exceeding MCII thresholds suggested potential clinical relevance. Nevertheless, further well-designed studies with larger sample sizes are necessary to substantiate these findings.

6. The short follow-up period (6 weeks post-intervention) limits assessment of the sustained effects of ILA treatment. I suggest clearly addressing this limitation and recommending longer follow-up durations (3–6 months or longer) in future studies to determine long-term treatment effects.

Response: We thank the reviewer for highlighting this important limitation

As suggested, we have clearly addressed this in the Limitation section of the Discussion and emphasized the necessity for extended follow-up durations (e.g., 3 to 6 months or longer) in future studies to better determine the long-term clinical outcomes.

(Page 23, Line 413-416)

Second, the follow-up duration in our study was relatively short. Consequently, this study has limitations in assessing sustained efficacy, long-term safety, and potential AEs of ILA. To determine the long-term efficacy and safety of ILA, future studies should incorporate extended follow-up periods such as three to six months or longer.

7. The evaluation of safety data is limited due to the study’s small sample size and short duration. I recommend explicitly noting this limitation in the manuscript and advising larger-scale trials with extended follow-up periods to robustly assess treatment safety and potential adverse events.

Response: We appreciate this comment, as it has allowed us to provide a more balanced view.

As recommended, we have revised the first and second paragraphs of the Limitation section to explicitly acknowledge that the small sample size and short follow-up period limit the robustness of safety assessment and the detection of potential adverse events. We have also emphasized the need for larger, multi-center trials with extended follow-up durations to more comprehensively evaluate long-term safety outcomes as per your suggestion.

(Page 23; Line 407-416)

Despite these advantages, our study has several limitations. First, we conducted only a pilot single-center study owing to the limitations of trial duration, research funding, and opportunities for recruitment. Therefore, the sample size and diversity were insufficient to fully validate the efficacy and safety of ILA in the treatment of KOA. These factors may have introduced bias into the trial results. In future studies, multi-center trials with larger sample sizes should be conducted to encompass a more diverse patient population, thereby enhancing the external validity and clinical applicability of the results. Second, the follow-up duration in our study was relatively short. Consequently, this study has limitations in assessing sustained efficacy, long-term safety, and potential AEs of ILA. To determine the long-term efficacy and safety of ILA, future studies should incorporate extended follow-up periods such as three to six months or longer.

8. This was a single-center study, limiting the generalizability of the results. I suggest clearly disc

Attachment Submitted filename: renamed_f8e96.docx

# Peer review: Decision Letter 1

**DOI:** 10.1371/journal.pone.0353654.r003

**Authors:** Ramada Rateb Khasawneh

29 Mar 2026

PONE-D-25-31350R1

Efficacy and safety of invasive laser acupuncture (650 and 830 nm) on knee osteoarthritis: A pilot randomized clinical trial

PLOS One

Dear Dr. Kim,

Thank you for submitting your manuscript to PLOS ONE. After careful consideration, we feel that it has merit but does not fully meet PLOS ONE’s publication criteria as it currently stands. Therefore, we invite you to submit a revised version of the manuscript that addresses the points raised during the review process.

==============================

My principal concern remains the absence of an appropriate sham control, specifically both a true sham acupuncture group and a sham laser group. In my view, such controls should involve the application of acupuncture (with or without laser) at non-acupoint locations. Without these comparator groups, the current study design does not adequately exclude the possibility that the observed effects are nonspecific and could similarly occur with stimulation at non-acupoint sites.

I find this particularly important given that, in the Discussion section, the authors attribute the therapeutic efficacy of both conventional and laser acupuncture to stimulation of defined acupoints. Moreover, the authors’ response to the previous reviewer comment on this issue appears inconsistent with their own interpretation presented in the Discussion. please answer this

==============================

Please submit your revised manuscript by May 13 2026 11:59PM. If you will need more time than this to complete your revisions, please reply to this message or contact the journal office at plosone@plos.org. When you're ready to submit your revision, log on to https://www.editorialmanager.com/pone/ and select the 'Submissions Needing Revision' folder to locate your manuscript file.

Please include the following items when submitting your revised manuscript:

- A letter that responds to each point raised by the academic editor and reviewer(s). You should upload this letter as a separate file labeled 'Response to Reviewers'.
- A marked-up copy of your manuscript that highlights changes made to the original version. You should upload this as a separate file labeled 'Revised Manuscript with Track Changes'.
- An unmarked version of your revised paper without tracked changes. You should upload this as a separate file labeled 'Manuscript'.

If you would like to make changes to your financial disclosure, please include your updated statement in your cover letter. Guidelines for resubmitting your figure files are available below the reviewer comments at the end of this letter.

If applicable, we recommend that you deposit your laboratory protocols in protocols.io to enhance the reproducibility of your results. Protocols.io assigns your protocol its own identifier (DOI) so that it can be cited independently in the future. For instructions see: https://journals.plos.org/plosone/s/submission-guidelines#loc-laboratory-protocols. Additionally, PLOS ONE offers an option for publishing peer-reviewed Lab Protocol articles, which describe protocols hosted on protocols.io. Read more information on sharing protocols at https://plos.org/protocols?utm_medium=editorial-email&utm_source=authorletters&utm_campaign=protocols.

As the corresponding author, your ORCID iD is verified in the submission system and will appear in the published article. PLOS supports the use of ORCID, and we encourage all coauthors to register for an ORCID iD and use it as well. Please encourage your coauthors to verify their ORCID iD within the submission system before final acceptance, as unverified ORCID iDs will not appear in the published article. *Only*  the individual author can complete the verification step; PLOS staff *cannot*  verify ORCID iDs on behalf of authors.

We look forward to receiving your revised manuscript.

Kind regards,

Ramada Rateb Khasawneh

Academic Editor

PLOS One

Journal Requirements:

If the reviewer comments include a recommendation to cite specific previously published works, please review and evaluate these publications to determine whether they are relevant and should be cited. There is no requirement to cite these works unless the editor has indicated otherwise.

Additional Editor Comments :

My principal concern remains the absence of an appropriate sham control, specifically both a true sham acupuncture group and a sham laser group. In my view, such controls should involve the application of acupuncture (with or without laser) at non-acupoint locations. Without these comparator groups, the current study design does not adequately exclude the possibility that the observed effects are nonspecific and could similarly occur with stimulation at non-acupoint sites.

I find this particularly important given that, in the Discussion section, the authors attribute the therapeutic efficacy of both conventional and laser acupuncture to stimulation of defined acupoints. Moreover, the authors’ response to the previous reviewer comment on this issue appears inconsistent with their own interpretation presented in the Discussion. .. please answer this

[Note: HTML markup is below. Please do not edit.]

Reviewers' comments:

Reviewer's Responses to Questions

**Comments to the Author**

1. If the authors have adequately addressed your comments raised in a previous round of review and you feel that this manuscript is now acceptable for publication, you may indicate that here to bypass the “Comments to the Author” section, enter your conflict of interest statement in the “Confidential to Editor” section, and submit your "Accept" recommendation.

Reviewer #1: All comments have been addressed

Reviewer #4: All comments have been addressed

**********

2. Is the manuscript technically sound, and do the data support the conclusions?

The manuscript must describe a technically sound piece of scientific research with data that supports the conclusions. Experiments must have been conducted rigorously, with appropriate controls, replication, and sample sizes. The conclusions must be drawn appropriately based on the data presented.

Reviewer #1: Yes

Reviewer #4: Yes

**********

3. Has the statistical analysis been performed appropriately and rigorously?

Reviewer #1: Yes

Reviewer #4: Yes

**********

4. Have the authors made all data underlying the findings in their manuscript fully available?

The PLOS Data policy requires authors to make all data underlying the findings described in their manuscript fully available without restriction, with rare exception (please refer to the Data Availability Statement in the manuscript PDF file). The data should be provided as part of the manuscript or its supporting information, or deposited to a public repository. For example, in addition to summary statistics, the data points behind means, medians and variance measures should be available. If there are restrictions on publicly sharing data—e.g. participant privacy or use of data from a third party—those must be specified.

Reviewer #1: Yes

Reviewer #4: Yes

**********

5. Is the manuscript presented in an intelligible fashion and written in standard English?

PLOS ONE does not copyedit accepted manuscripts, so the language in submitted articles must be clear, correct, and unambiguous. Any typographical or grammatical errors should be corrected at revision, so please note any specific errors here.

Reviewer #1: Yes

Reviewer #4: Yes

**********

6. Review Comments to the Author

Please use the space provided to explain your answers to the questions above. You may also include additional comments for the author, including concerns about dual publication, research ethics, or publication ethics. (Please upload your review as an attachment if it exceeds 20,000 characters)

Reviewer #1: Thank you for carefully addressing all the major concerns from my previous review. It is optional that if you want to further strengthen the manuscript, I suggest explicitly reinforcing the necessity of sensitivity analyses for missing data handling in future research, as well as clearly emphasizing the importance of assessing sustained long-term outcomes.

Reviewer #4: After reviewing the revised manuscript and the authors’ responses to the reviewers’ comments, I believe that the authors have satisfactorily addressed the concerns raised in the previous round of review. The revisions have improved the clarity of the manuscript, particularly with regard to methodological explanations, reporting according to relevant guidelines, and discussion of the study limitations.

Although the study has inherent limitations associated with its pilot design and relatively small sample size, these issues are now appropriately acknowledged and discussed by the authors. The statistical analyses and presentation of results are generally adequate for an exploratory pilot randomized clinical trial, and the conclusions have been revised to reflect the preliminary nature of the findings.

Overall, the manuscript provides useful preliminary evidence regarding the potential efficacy and safety of invasive laser acupuncture for knee osteoarthritis and may serve as a basis for future larger-scale trials.

Therefore, I recommend acceptance of the manuscript for publication.

**********

7. PLOS authors have the option to publish the peer review history of their article (what does this mean?). If published, this will include your full peer review and any attached files.

If you choose “no”, your identity will remain anonymous but your review may still be made public.

**Do you want your identity to be public for this peer review?**  For information about this choice, including consent withdrawal, please see our Privacy Policy.

Reviewer #1: No

Reviewer #4: **Yes:** Dr.Fawwaz Al-Smadi

**********

[NOTE: If reviewer comments were submitted as an attachment file, they will be attached to this email and accessible via the submission site. Please log into your account, locate the manuscript record, and check for the action link "View Attachments". If this link does not appear, there are no attachment files.]

To ensure your figures meet our technical requirements, please review our figure guidelines: https://journals.plos.org/plosone/s/figures

You may also use PLOS’s free figure tool, NAAS, to help you prepare publication quality figures: https://journals.plos.org/plosone/s/figures#loc-tools-for-figure-preparation.

NAAS will assess whether your figures meet our technical requirements by comparing each figure against our figure specifications.

# Author comment: Author response to Decision Letter 2

**DOI:** 10.1371/journal.pone.0353654.r004

3 Apr 2026

POINT -BY -POINT RESPONSES

Dear editor and reviewers

We thank you for reviewing our manuscript and for your constructive comments, which have helped improve the manuscript. We have carefully reviewed the comments and have accordingly revised the manuscript. Changes are tracked in the revised manuscript. Below is a detailed, point-by-point response to your comments. We hope that our revisions meet your expectations.

Additional Editor Comments:

My principal concern remains the absence of an appropriate sham control, specifically both a true sham acupuncture group and a sham laser group. In my view, such controls should involve the application of acupuncture (with or without laser) at non-acupoint locations. Without these comparator groups, the current study design does not adequately exclude the possibility that the observed effects are nonspecific and could similarly occur with stimulation at non-acupoint sites.

I find this particularly important given that, in the Discussion section, the authors attribute the therapeutic efficacy of both conventional and laser acupuncture to stimulation of defined acupoints. Moreover, the authors’ response to the previous reviewer comment on this issue appears inconsistent with their own interpretation presented in the Discussion. .. please answer this

Response: We apologize for the inconsistency in our previous response

We fully acknowledge the Editor’s concern regarding the absence of a non-acupoint sham control group. As this was an exploratory pilot study, our primary objective was to investigate the wavelength-dependent effects of ILA (650 and 830 nm) compared with those of a sham laser control; therefore, we prioritized these comparators within our limited resources. We agree that without a non-acupoint control, we cannot definitively dismiss non-specific effects.

To address this, we have made the following revisions:

1. We have revised the Abstract and Introduction to more clearly define the specific scope and aims of this pilot study, focusing on the comparative efficacy of the two wavelengths. The revised part is as follows:

This pilot study explored the safety and efficacy of invasive laser acupuncture (ILA) using two wavelengths (650 and 830 nm) compared with those of a sham laser in patients with knee osteoarthritis (KOA). (Page 3, Line 36-38)

Therefore, this exploratory pilot study aimed to explore wavelength-dependent differences in the safety and efficacy of ILA (650 and 830 nm) compared with those of a sham laser control in patients with KOA. (Page 5, Line 84-86)

2. We have added a detailed explanation in the Discussion section (under the fifth limitation) to explicitly acknowledge the lack of a non-acupoint control and the resulting inability to distinguish between site-specific and non-specific responses. The added part is as follows:

Fifth, LLLT application is influenced by a complex combination of parameters, delivery techniques, and irradiation sites [38]. As this exploratory pilot trial was primarily designed to investigate the wavelength-dependent effects of ILA, we prioritized comparing the 650 and 830 nm groups against a sham laser control. Consequently, our study did not incorporate a non-acupoint sham control, limiting our ability to determine whether the observed improvements were attributable to specific acupoints or to non-specific deep-tissue stimulation. Future studies incorporating sham acupoint designs are warranted to clarify the role of site-specificity. (Page 24, Line 427-434)

3. We have revised the Discussion to ensure consistency and to align our interpretation with the actual study design. The added text is as follows:

Effective delivery of energy to deeper structures—such as the synovium, articular cartilage, and muscle, which are pathologic sites in KOA—is important, as more than 90% of the energy from lasers used in musculoskeletal applications is absorbed within the first 10 mm of tissue [35]. (Page 21, Line 371-373)

Although no significant intergroup differences in the WOMAC function subscale and EQ-5D-5L scores were observed at Visits 7 and 13, substantial differences were observed at Visit 14. These findings may have been influenced by the common treatment in all groups, which included teaching of self-management and self-exercise—as strongly recommended in the ACR guidelines for KOA—during the intervention period [3]. (Page 22, Line 389-393)

Review Comments to the Author

Reviewer #1:

Thank you for carefully addressing all the major concerns from my previous review. It is optional that if you want to further strengthen the manuscript, I suggest explicitly reinforcing the necessity of sensitivity analyses for missing data handling in future research, as well as clearly emphasizing the importance of assessing sustained long-term outcomes.:

Response: We would like to thank the reviewer for evaluating our manuscript and for the insightful

To strengthen the manuscript, we have addressed the requirement of sensitivity analyses for missing data handling in future research. Moreover, we have also emphasized the importance of sustained long-term outcomes in the second limitation. The revised text is as follows:

Sixth, missing data were managed using the LOCF method, which is considered suboptimal compared to more robust approaches, such as multiple imputation or mixed-effects modeling [41]. Future studies with more advanced statistical methods are needed to better establish the efficacy of ILA on KOA. (Page 24, Line 434-437)

Second, the follow-up duration in our study was relatively short. Consequently, this study has limitations in assessing sustained efficacy, long-term safety, and potential AEs of ILA. To determine the long-term efficacy and safety of ILA, future studies should incorporate extended follow-up periods such as three to six months or longer. (Page 23, Line 414-417)

Reviewer #4:

After reviewing the revised manuscript and the authors’ responses to the reviewers’ comments, I believe that the authors have satisfactorily addressed the concerns raised in the previous round of review. The revisions have improved the clarity of the manuscript, particularly with regard to methodological explanations, reporting according to relevant guidelines, and discussion of the study limitations.

Although the study has inherent limitations associated with its pilot design and relatively small sample size, these issues are now appropriately acknowledged and discussed by the authors. The statistical analyses and presentation of results are generally adequate for an exploratory pilot randomized clinical trial, and the conclusions have been revised to reflect the preliminary nature of the findings.

Overall, the manuscript provides useful preliminary evidence regarding the potential efficacy and safety of invasive laser acupuncture for knee osteoarthritis and may serve as a basis for future larger-scale trials.

Therefore, I recommend acceptance of the manuscript for publication.

Response: We are deeply grateful for the reviewer’s positive assessment of our manuscript

Attachment Submitted filename: renamed_b7369.docx

# Peer review: Decision Letter 2

**DOI:** 10.1371/journal.pone.0353654.r005

**Authors:** Ramada Rateb Khasawneh

12 May 2026

PONE-D-25-31350R2

Efficacy and safety of invasive laser acupuncture (650 and 830 nm) on knee osteoarthritis: A pilot randomized clinical trial

PLOS One

Dear Dr. Kim,

Thank you for submitting your manuscript to PLOS ONE. After careful consideration, we feel that it has merit but does not fully meet PLOS ONE’s publication criteria as it currently stands. Therefore, we invite you to submit a revised version of the manuscript that addresses the points raised during the review process.

Please submit your revised manuscript by Jun 26 2026 11:59PM. If you will need more time than this to complete your revisions, please reply to this message or contact the journal office at plosone@plos.org. When you're ready to submit your revision, log on to https://www.editorialmanager.com/pone/ and select the 'Submissions Needing Revision' folder to locate your manuscript file.

Please include the following items when submitting your revised manuscript:

- A letter that responds to each point raised by the academic editor and reviewer(s). You should upload this letter as a separate file labeled 'Response to Reviewers'.
- A marked-up copy of your manuscript that highlights changes made to the original version. You should upload this as a separate file labeled 'Revised Manuscript with Track Changes'.
- An unmarked version of your revised paper without tracked changes. You should upload this as a separate file labeled 'Manuscript'.

If you would like to make changes to your financial disclosure, please include your updated statement in your cover letter. Guidelines for resubmitting your figure files are available below the reviewer comments at the end of this letter.

If applicable, we recommend that you deposit your laboratory protocols in protocols.io to enhance the reproducibility of your results. Protocols.io assigns your protocol its own identifier (DOI) so that it can be cited independently in the future. For instructions see: https://journals.plos.org/plosone/s/submission-guidelines#loc-laboratory-protocols. Additionally, PLOS ONE offers an option for publishing peer-reviewed Lab Protocol articles, which describe protocols hosted on protocols.io. Read more information on sharing protocols at https://plos.org/protocols?utm_medium=editorial-email&utm_source=authorletters&utm_campaign=protocols.

As the corresponding author, your ORCID iD is verified in the submission system and will appear in the published article. PLOS supports the use of ORCID, and we encourage all coauthors to register for an ORCID iD and use it as well. Please encourage your coauthors to verify their ORCID iD within the submission system before final acceptance, as unverified ORCID iDs will not appear in the published article. *Only*  the individual author can complete the verification step; PLOS staff *cannot*  verify ORCID iDs on behalf of authors.

We look forward to receiving your revised manuscript.

Kind regards,

Ramada Rateb Khasawneh

Academic Editor

PLOS One

Journal Requirements:

1. If the reviewer comments include a recommendation to cite specific previously published works, please review and evaluate these publications to determine whether they are relevant and should be cited. There is no requirement to cite these works unless the editor has indicated otherwise.

2. Please review your reference list to ensure that it is complete and correct. If you have cited papers that have been retracted, please include the rationale for doing so in the manuscript text, or remove these references and replace them with relevant current references. Any changes to the reference list should be mentioned in the rebuttal letter that accompanies your revised manuscript. If you need to cite a retracted article, indicate the article’s retracted status in the References list and also include a citation and full reference for the retraction notice.

Additional Editor Comments:

Could you please address the issues raised by the reviewer?

[Note: HTML markup is below. Please do not edit.]

Reviewers' comments:

Reviewer's Responses to Questions

**Comments to the Author**

1. If the authors have adequately addressed your comments raised in a previous round of review and you feel that this manuscript is now acceptable for publication, you may indicate that here to bypass the “Comments to the Author” section, enter your conflict of interest statement in the “Confidential to Editor” section, and submit your "Accept" recommendation.

Reviewer #1: All comments have been addressed

Reviewer #5: (No Response)

**********

2. Is the manuscript technically sound, and do the data support the conclusions?

The manuscript must describe a technically sound piece of scientific research with data that supports the conclusions. Experiments must have been conducted rigorously, with appropriate controls, replication, and sample sizes. The conclusions must be drawn appropriately based on the data presented.

Reviewer #1: Yes

Reviewer #5: Partly

**********

3. Has the statistical analysis been performed appropriately and rigorously?

Reviewer #1: Yes

Reviewer #5: No

**********

4. Have the authors made all data underlying the findings in their manuscript fully available?

The PLOS Data policy requires authors to make all data underlying the findings described in their manuscript fully available without restriction, with rare exception (please refer to the Data Availability Statement in the manuscript PDF file). The data should be provided as part of the manuscript or its supporting information, or deposited to a public repository. For example, in addition to summary statistics, the data points behind means, medians and variance measures should be available. If there are restrictions on publicly sharing data—e.g. participant privacy or use of data from a third party—those must be specified.

Reviewer #1: Yes

Reviewer #5: Yes

**********

5. Is the manuscript presented in an intelligible fashion and written in standard English?

PLOS ONE does not copyedit accepted manuscripts, so the language in submitted articles must be clear, correct, and unambiguous. Any typographical or grammatical errors should be corrected at revision, so please note any specific errors here.

Reviewer #1: Yes

Reviewer #5: Yes

**********

6. Review Comments to the Author

Please use the space provided to explain your answers to the questions above. You may also include additional comments for the author, including concerns about dual publication, research ethics, or publication ethics. (Please upload your review as an attachment if it exceeds 20,000 characters)

Reviewer #1: The authors have sufficiently addressed all of my questions. I do not have any additional concerns or questions.

Reviewer #5: General Assessment

This manuscript presents a pilot randomized, placebo-controlled clinical trial evaluating the efficacy and safety of invasive laser acupuncture (ILA) at two wavelengths (650 and 830 nm) in patients with knee osteoarthritis (KOA). The topic is clinically relevant, and the randomized design with a sham control is a strength. However, several methodological and reporting limitations reduce confidence in the robustness and interpretation of the findings. In its current form, the manuscript would benefit from substantial revision.

Major Comments

Sample Size and Statistical Power

The study includes a total of 45 participants (approximately 15 per group). The manuscript does not clearly report an a priori sample size calculation. Given the small sample size, the study is likely underpowered, increasing the risk of both type I and type II errors.

Multiple Comparisons and Risk of False Positives

Multiple outcomes are assessed across several time points without clear indication of correction for multiple comparisons. This raises concern about inflated type I error rates.

Interpretation of Findings

As a pilot study, conclusions should be more cautious and emphasize the exploratory nature of the findings rather than suggesting efficacy.

Randomization and Allocation Concealment

The manuscript lacks sufficient detail regarding the randomization process and allocation concealment.

Blinding

Further clarification is needed on how blinding was implemented and whether its effectiveness was assessed.

Clinical Relevance

The manuscript should address whether observed differences meet minimal clinically important differences (MCID).

Biological Plausibility

A stronger discussion of the biological mechanism underlying the intervention is recommended.

Minor Comments

Clarify treatment duration wording

Define acupuncture point abbreviations

Clarify rescue medication use

Include CONSORT flow diagram

Minor language edits recommended

**********

7. PLOS authors have the option to publish the peer review history of their article (what does this mean?). If published, this will include your full peer review and any attached files.

If you choose “no”, your identity will remain anonymous but your review may still be made public.

**Do you want your identity to be public for this peer review?**  For information about this choice, including consent withdrawal, please see our Privacy Policy.

Reviewer #1: No

Reviewer #5: No

**********

[NOTE: If reviewer comments were submitted as an attachment file, they will be attached to this email and accessible via the submission site. Please log into your account, locate the manuscript record, and check for the action link "View Attachments". If this link does not appear, there are no attachment files.]

To ensure your figures meet our technical requirements, please review our figure guidelines: https://journals.plos.org/plosone/s/figures

You may also use PLOS’s free figure tool, NAAS, to help you prepare publication quality figures: https://journals.plos.org/plosone/s/figures#loc-tools-for-figure-preparation.

NAAS will assess whether your figures meet our technical requirements by comparing each figure against our figure specifications.

# Author comment: Author response to Decision Letter 3

**DOI:** 10.1371/journal.pone.0353654.r006

22 May 2026

POINT-BY-POINT RESPONSES

Dear editor and reviewers

We thank you for reviewing our manuscript and for your constructive comments, which have helped improve the manuscript. We have carefully reviewed the comments and have accordingly revised the manuscript. Changes are tracked in the revised manuscript. Below is a detailed, point-by-point response to your comments. We hope that our revisions meet your expectations.

Additional Editor Comments:

Could you please address the issues raised by the reviewer?

Response:

We sincerely thank the Academic Editor for handling our manuscript across multiple revision rounds. We have carefully addressed each of the points raised by Reviewer #5, including those regarding sample size and statistical power, multiple comparisons, interpretation of findings, randomization and allocation concealment, blinding, clinical relevance, biological plausibility, and the minor comments. Detailed responses to each comment, along with the corresponding revisions to the manuscript, are provided below. We hope that the revised manuscript and our responses adequately address all concerns raised during this review round.

Review Comments to the Author

Reviewer #1:

The authors have sufficiently addressed all of my questions. I do not have any additional concerns or questions.

Response:

We sincerely thank you for the careful review of our manuscript across multiple revision rounds and for confirming that our previous responses have adequately addressed all concerns.

Reviewer #5:

General Assessment

This manuscript presents a pilot randomized, placebo-controlled clinical trial evaluating the efficacy and safety of invasive laser acupuncture (ILA) at two wavelengths (650 and 830 nm) in patients with knee osteoarthritis (KOA). The topic is clinically relevant, and the randomized design with a sham control is a strength. However, several methodological and reporting limitations reduce confidence in the robustness and interpretation of the findings. In its current form, the manuscript would benefit from substantial revision.

Major Comments

Sample Size and Statistical Power

The study includes a total of 45 participants (approximately 15 per group). The manuscript does not clearly report an a priori sample size calculation. Given the small sample size, the study is likely underpowered, increasing the risk of both type I and type II errors.

Response:

We sincerely thank the reviewer for this thoughtful comment, and we apologize if our sample size rationale was not sufficiently clear in the original manuscript.

We fully agree that the sample size of the present study is not sufficient to confirm the efficacy of ILA for KOA, and this limitation is acknowledged in the manuscript. For this reason, we adopted an exploratory pilot study design, as explicitly stated in the title, abstract, and main text.

This trial aimed to assess feasibility, estimate preliminary effect sizes, and inform the design of future definitive trials, rather than to provide confirmatory evidence of efficacy. Confirmation of efficacy will require an adequately powered follow-up trial.

An a priori sample size justification is provided in the "Sample size calculation" section. As no prior trials of ILA for KOA were available to inform a conventional power calculation, we followed the established rule-of-thumb for pilot studies, allocating 15 participants per group based on the recommended minimum of 12 per arm [28, 29], with an additional 20% allowance for attrition.

To further clarify the exploratory nature and future direction of this trial, we have revised the following sentence to the "Sample size calculation" section:

We adopted a pilot study design due to the absence of previous studies, limited budgets, and enrollment opportunities. In a three-arm pilot study, the adequate sample size is more than 12 [28, 29]. Assuming that 20% of the participants may drop out, we allocated 15 participants to each group.

Because this study was a pilot study, the sample size was not enough to evaluate the effectiveness and safety of ILA for KOA. However, our pilot study may establish the feasibility of a larger, rigorously designed confirmatory RCT on ILA in patients with KOA and provide preliminary insight into the acceptability and safety of ILA for KOA patients. (Page 14; Line 249-256)

Multiple Comparisons and Risk of False Positives

Multiple outcomes are assessed across several time points without clear indication of correction for multiple comparisons. This raises concern about inflated type I error rates.

Response:

We truly appreciate this important methodological comment. We acknowledge that assessing multiple outcomes across several time points without formal correction for multiple comparisons may inflate the type I error rate.

As an exploratory pilot trial, the aim of the present study was not to provide confirmatory evidence of efficacy, but to assess feasibility, estimate preliminary effect sizes, and inform the design of future definitive trials. Accordingly, the statistically significant findings observed in this study should be interpreted as preliminary and hypothesis-generating rather than confirmatory.

To transparently address this point, we have added the following statement as the seventh limitation.

Seventh, this exploratory pilot trial measured multiple outcomes at several time points without formal adjustment for multiple comparisons. Therefore, the possibility of inflated type I error and false-positive findings should be considered when interpreting the results. The statistically significant findings observed in this study should be regarded as preliminary and hypothesis-generating rather than confirmatory. Future adequately powered confirmatory trials with prespecified hierarchical testing strategies or multiplicity adjustment methods are warranted. (Page 26; Line 474-480)

Interpretation of Findings

As a pilot study, conclusions should be more cautious and emphasize the exploratory nature of the findings rather than suggesting efficacy.

Response:

We fully agree that the conclusions should more clearly reflect the exploratory and preliminary nature of this pilot trial. Accordingly, we have revised the relevant sections to soften the wording and emphasize the exploratory nature of the findings.

Abstract

The 650 and 830 nm ILA exhibited potential short-term improvement in pain reduction. Additionally, partial short-term improvements in pain-related functional disabilities and quality of life were observed. However, these preliminary findings should be confirmed in larger, rigorously designed clinical trials. (Page 3; Line 54-57)

Conclusion

In this exploratory pilot trial, ILA at 650 and 830 nm showed preliminary signals of the potential for a short-term improvement in pain reduction among patients with KOA. Additionally, partial but exploratory short-term improvements were observed in pain-related functional disability and quality of life. However, as we conducted a pilot study with a small sample size and exploratory design, adequately powered, confirmatory trials are warranted to validate these preliminary observations. (Page 26; Line 483-488)

Randomization and Allocation Concealment

The manuscript lacks sufficient detail regarding the randomization process and allocation concealment.

Response:

We agree that the randomization and allocation concealment procedures warranted a more detailed description. Accordingly, we have substantially revised the relevant section in the Methods.

The revised passage is as follows:

The 45 eligible participants were randomly assigned to three groups (control [0 mW power sham laser], 650 [20 mW power, 650 nm wavelength laser], or 830 [20 mW power, 830 nm wavelength laser]) at a 1:1:1 ratio. The randomization sequence was generated by SASⓇ (Version 9.4, SAS Institute, Cary, NC, USA) with block randomization by a statistician who did not participate in the trial. Allocation concealment was ensured through the following procedure. Each allocation code was placed inside an opaque envelope, which was then sealed and labeled with a sequential number. The envelopes were stored in a double-locked cabinet. At the point of enrollment—immediately after a participant had completed baseline measurements and provided informed consent—a designated randomization administrator, who took no part in screening and intervention, outcome assessment, or data analysis, retrieved the next envelope in numerical order, opened it, and recorded the date and signature on a log sheet. The group assignment was disclosed only to the practitioner who delivered the intervention. (Page 10-11; Line 172-183)

Blinding

Further clarification is needed on how blinding was implemented and whether its effectiveness was assessed.

Response:

As per your suggestion, we have expanded the Blinding section in the Methods to provide a more detailed description of how participant and outcome assessor blinding was implemented, and we have explicitly acknowledged that the effectiveness of blinding was not formally assessed using a validated blinding index. This point has also been added to the Limitations section. The revised passages are as follows:

Because of the nature of the ILA treatment, in which the practitioner had to operate the laser device (sham, 650 nm, or 830 nm laser) after inserting the needle, blinding of the practitioner was not feasible. To minimize potential bias, we conducted a patient- and assessor-blinded trial. Participants could not feel any specific sensation distinguishing between the real laser and sham laser, and the device was configured to generate identical operational noise across all groups. In addition, participants wore a blindfold during the procedure to block visual identification of the intervention. Through these methods, participants were unable to distinguish between treatment conditions. The outcome assessor was not in contact with any participant except during the assessment time points. Thus, until trial completion, both participants and assessor were blinded to the group allocation. Formal assessment of blinding effectiveness using a validated blinding index was not performed in this pilot trial. (Page 11; Line 184-194)

Third, owing to the characteristics of ILA, it was not possible to blind the practitioners, as they had to directly operate the laser device. To minimize this risk, we adopted an outcome assessor-blinded design. Nevertheless, the absence of practitioner blinding remains a methodological limitation and may have influenced the trial results. In addition, although we implemented multiple measures to maintain participant and assessor blinding, the effectiveness of blinding was not evaluated using a validated blinding index. Therefore, we could not verify whether the blinding procedures were fully successful. Further research should explore strategies for blinding practitioners when possible, consider the inclusion of independent outcome assessments or more objective outcome measures, and assess blinding effectiveness to further mitigate potential bias. (Page 25; Line 451-460)

Clinical Relevance

The manuscript should address whether observed differences meet minimal clinically important differences (MCID).

Response:

We sincerely thank you for raising this point. The clinical relevance of the within-group changes was previously addressed in the Discussion section using the minimal clinically important improvement (MCII) thresholds for KOA [40]. To further address the reviewer's concern regarding between-group differences (MCID), we have added a detailed analysis in the Discussion based on the MCID values reported in a recent meta-analysis of placebo-controlled trials in KOA. At the primary endpoint (Visit 13), most between-group differences (vs. control) exceeded the MCID thresholds, including VAS during activity and all WOMAC subscales. For VAS at rest, the 830 group difference exceeded the MCID threshold, while the 650 group difference marginally fell below it; this finding has been reported in the revised Discussion. The added passage is as follows:

The responder rates were 26.67% in the control group, 53.33% in the 650 group, and 60% in the 830 group. Although the experimental groups showed numerically higher rates compared to the control group, the difference did not reach statistical significance. This lack of significance may be attributed to the small sample size. Minimal clinically important improvement (MCII), defined as the smallest change in measurement that signifies an important improvement in a patient’s symptom, for KOA is established at -19.9 mm for pain VAS and -9.1 for the WOMAC function subscale [40]. In our study, the 650 group showed reductions of -20.7 mm in VAS at rest, -21.7 mm in VAS during activity, and -14.9 in the WOMAC function subscale. Similarly, the 830 group showed reductions of -22.6 mm in VAS at rest, -22.9 mm in VAS during activity, and -14.9 in the WOMAC function subscale. These improvements observed in both experimental groups exceeded the established MCII thresholds. Despite the lack of a statistically significant difference in responder rates, exceeding MCII thresholds suggested potential clinical relevance. In addition to MCII, minimal clinically important difference (MCID) values for KOA have been reported as VAS 13.7 mm, WOMAC total 6.4, WOMAC pain 1.5, and WOMAC function 4.6 [41]. At the primary endpoint (Visit 13), the between group differences (vs. control) exceeded these MCID thresholds for most outcomes: VAS during activity (15.0 mm, 650 group; 16.2 mm, 830 group), WOMAC total (13.1, 650 group; 11.8, 830 group), WOMAC pain (3.4, 650 group; 2.4, 830 group), and WOMAC function (8.4 for both groups). For VAS at rest, the difference in the 830 group (15.4 mm) exceeded the MCID threshold, while the difference in the 650 group (13.5 mm) closely approached but marginally fell below the threshold. Considering the clinically meaningful differences across most outcomes, these findings also suggested potential clinical relevance. Nevertheless, further well-designed studies with larger sample sizes are necessary to substantiate these findings. (Page 23-24; Line 419-441)

Biological Plausibility

A stronger discussion of the biological mechanism underlying the intervention is recommended.

Response:

We fully agree that a more detailed discussion of the biological mechanism would strengthen the manuscript. Accordingly, we have substantially expanded the relevant passage in the Discussion section to provide a more comprehensive account of photobiomodulation (PBM) mechanisms. The revised passage is as follows:

The reasons behind the reduction in the VAS score and WOMAC total scores are likely attributed to the analgesic and anti-inflammatory effects of photobiomodulation (PBM). PBM exerts several effects on cells by therapeutically exposing cells to low levels of red or near-infrared light. First, in the intracellular compartment, PBM light is thought to modulate cytochrome c oxidase activity in mitochondria, thereby increasing adenosine triphosphate production and modulating intracellular levels of reactive oxygen species. Through this pathway, PBM has been reported to modulate the production of key pro-inflammatory mediators, including prostaglandin E2, interleukin-6, and tumor necrosis factor-alpha. Second, at the cell membrane, PBM may modulate ion channels such as transient receptor potential vanilloid 1, thereby attenuating nociceptor excitability and contributing to analgesia. Third, in the extracellular compartment, PBM may activate latent transforming growth factor-β1 through a redox-dependent activation mechanism, which promotes tissue repair and regeneration. Through these molecular mechanisms acting at multiple cellular compartments, PBM exerts analgesic, anti-inflammatory, and reparative effects, which may contribute to the symptomatic improvement observed in patients with KOA [30-33]. (Page 21-22; Line 376-390)

Minor Comments

Clarify treatment duration wording

Response:

We have revised the treatment duration wording throughout the manuscript to improve clarity. The revised wording is now consistently applied across the Abstract, Methods (Study design), and Methods (Intervention) sections

Attachment Submitted filename: Response_to_Reviewer_ILA_KOA_R3_Final.docx

# Peer review: Decision Letter 3

**DOI:** 10.1371/journal.pone.0353654.r007

**Authors:** Ramada Rateb Khasawneh

9 Jun 2026

PONE-D-25-31350R3

Efficacy and safety of invasive laser acupuncture (650 and 830 nm) on knee osteoarthritis: A pilot randomized clinical trial

PLOS One

Dear Dr. Kim,

Thank you for submitting your manuscript to PLOS ONE. After careful consideration, we feel that it has merit but does not fully meet PLOS ONE’s publication criteria as it currently stands. Therefore, we invite you to submit a revised version of the manuscript that addresses the points raised during the review process.

Please submit your revised manuscript by Jul 24 2026 11:59PM. If you will need more time than this to complete your revisions, please reply to this message or contact the journal office at plosone@plos.org. When you're ready to submit your revision, log on to https://www.editorialmanager.com/pone/ and select the 'Submissions Needing Revision' folder to locate your manuscript file.

Please include the following items when submitting your revised manuscript:

- A letter that responds to each point raised by the academic editor and reviewer(s). You should upload this letter as a separate file labeled 'Response to Reviewers'.
- A marked-up copy of your manuscript that highlights changes made to the original version. You should upload this as a separate file labeled 'Revised Manuscript with Track Changes'.
- An unmarked version of your revised paper without tracked changes. You should upload this as a separate file labeled 'Manuscript'.

If you would like to make changes to your financial disclosure, please include your updated statement in your cover letter. Guidelines for resubmitting your figure files are available below the reviewer comments at the end of this letter.

If applicable, we recommend that you deposit your laboratory protocols in protocols.io to enhance the reproducibility of your results. Protocols.io assigns your protocol its own identifier (DOI) so that it can be cited independently in the future. For instructions see: https://journals.plos.org/plosone/s/submission-guidelines#loc-laboratory-protocols. Additionally, PLOS ONE offers an option for publishing peer-reviewed Lab Protocol articles, which describe protocols hosted on protocols.io. Read more information on sharing protocols at https://plos.org/protocols?utm_medium=editorial-email&utm_source=authorletters&utm_campaign=protocols.

As the corresponding author, your ORCID iD is verified in the submission system and will appear in the published article. PLOS supports the use of ORCID, and we encourage all coauthors to register for an ORCID iD and use it as well. Please encourage your coauthors to verify their ORCID iD within the submission system before final acceptance, as unverified ORCID iDs will not appear in the published article. *Only*  the individual author can complete the verification step; PLOS staff *cannot*  verify ORCID iDs on behalf of authors.

We look forward to receiving your revised manuscript.

Kind regards,

Ramada Rateb Khasawneh

Academic Editor

PLOS One

Journal Requirements:

If the reviewer comments include a recommendation to cite specific previously published works, please review and evaluate these publications to determine whether they are relevant and should be cited. There is no requirement to cite these works unless the editor has indicated otherwise.

Please review your reference list to ensure that it is complete and correct. If you have cited papers that have been retracted, please include the rationale for doing so in the manuscript text, or remove these references and replace them with relevant current references. Any changes to the reference list should be mentioned in the rebuttal letter that accompanies your revised manuscript. If you need to cite a retracted article, indicate the article’s retracted status in the References list and also include a citation and full reference for the retraction notice.

Additional Editor Comments:

Could you please address the issue raised by the reviewer?

[Note: HTML markup is below. Please do not edit.]

Reviewers' comments:

Reviewer's Responses to Questions

**Comments to the Author**

1. If the authors have adequately addressed your comments raised in a previous round of review and you feel that this manuscript is now acceptable for publication, you may indicate that here to bypass the “Comments to the Author” section, enter your conflict of interest statement in the “Confidential to Editor” section, and submit your "Accept" recommendation.

Reviewer #1: All comments have been addressed

Reviewer #6: All comments have been addressed

Reviewer #7: (No Response)

Reviewer #8: All comments have been addressed

**********

2. Is the manuscript technically sound, and do the data support the conclusions?

The manuscript must describe a technically sound piece of scientific research with data that supports the conclusions. Experiments must have been conducted rigorously, with appropriate controls, replication, and sample sizes. The conclusions must be drawn appropriately based on the data presented.

Reviewer #1: Yes

Reviewer #6: Yes

Reviewer #7: (No Response)

Reviewer #8: Yes

**********

3. Has the statistical analysis been performed appropriately and rigorously?

Reviewer #1: Yes

Reviewer #6: Yes

Reviewer #7: (No Response)

Reviewer #8: Yes

**********

4. Have the authors made all data underlying the findings in their manuscript fully available?

The PLOS Data policy requires authors to make all data underlying the findings described in their manuscript fully available without restriction, with rare exception (please refer to the Data Availability Statement in the manuscript PDF file). The data should be provided as part of the manuscript or its supporting information, or deposited to a public repository. For example, in addition to summary statistics, the data points behind means, medians and variance measures should be available. If there are restrictions on publicly sharing data—e.g. participant privacy or use of data from a third party—those must be specified.

Reviewer #1: Yes

Reviewer #6: Yes

Reviewer #7: (No Response)

Reviewer #8: Yes

**********

5. Is the manuscript presented in an intelligible fashion and written in standard English?

PLOS ONE does not copyedit accepted manuscripts, so the language in submitted articles must be clear, correct, and unambiguous. Any typographical or grammatical errors should be corrected at revision, so please note any specific errors here.

Reviewer #1: Yes

Reviewer #6: Yes

Reviewer #7: (No Response)

Reviewer #8: Yes

**********

6. Review Comments to the Author

Please use the space provided to explain your answers to the questions above. You may also include additional comments for the author, including concerns about dual publication, research ethics, or publication ethics. (Please upload your review as an attachment if it exceeds 20,000 characters)

Reviewer #1: The authors have fully addressed each of my comments and concerns, I do not have any other questions.

Reviewer #6: Thank you for the detailed revision and thoughtful point-by-point responses. The manuscript has improved substantially, addressing all comments of reviewers.

Reviewer #7: (No Response)

Reviewer #8: As an ad-hoc reviewer invited during the third revision, I have focused my evaluation on the remaining scientific soundness, methodological queries, and logical structure of the manuscript.

1. Strengths

KOA is a major degenerative disorder affecting a significant portion of the population.

This pilot trial is well-designed to evaluate the feasibility of two different wavelengths of laser acupuncture, utilizing active acupuncture as a sham laser control for KOA patients.

The efficient delivery of laser treatment via acupuncture needles is a notable strength of this study. It suggests a potential for reducing KOA-related pain and improving QoL, thereby contributing valuable insights to the field of KOA treatment.

I hope this study, along with further follow-up research on ILA, will offer significant clinical relevance, impact, and a novel integrative approach to KOA management.

Throughout the initial and revised manuscripts, the authors have demonstrated transparency and scientific rigor by providing detailed CONSORT and STRICTA-compliant methods, comprehensive supplementary materials (including the study protocol and individual data), and plausible biological mechanisms to ensure the robustness of the trial.

Based on my review of the manuscript, I would like to provide some recommendations to further improve the paper.

2. Weaknesses and Suggestions

1) Major Issues

- The concern regarding the inflation of the Type I error rate due to multiple comparisons of the same outcome across a continuous time series against the baseline remains unaddressed.

Although the authors previously responded to a similar point by expanding the limitations section, and this exploratory perspective of this pilot trial is understood and acceptable to some extent, the statistical validity of the findings may still be compromised by this inflated error rate.

Given that the study tracks indicators over time but relies solely on independent, point-by-point ANCOVA at each time interval, please consider utilizing a linear mixed-effects model or repeated measures ANCOVA to evaluate the time-by-group interaction effect if a formal re-analysis is feasible.

If a full re-analysis is realistically difficult at this stage, the statement of this methodological limitation must be further strengthened. Furthermore, the discussion and conclusion must be rephrased in a conservative tone, explicitly cautioning readers that the individual p-values at each visit were not adjusted for multiple temporal comparisons.

- While KOA improvement would be generally expected to influence QoL, the actual improvement in QoL achieved through ILA and conventional acupuncture was limited.

Please discuss the potential ceiling effect of the EQ-5D-5L in this patient population and the limitations of MCID,

and whether EQ-VAS should be considered as complementary outcome to capture more subtle changes for further study?

- The authors provided detailed reporting on AEs with V/S and laboratory results, clarifying that no significant changes were observed among the three groups in their Discussion section and Table S3.

However, for more conservative safety evaluation, tracking individual clinical change events (or outlier shifts) is more clinically relevant than merely reporting the intergroup statistical significance of means and p-values of vital signs and lab results.

- The purpose and impact of including bilateral KOA patients in a unilateral treatment trial: "treated with ILA on one-side (more affected side)"

What was the rationale for including patients with bilateral KOA in this trial? (Please elaborate if this was chosen to enhance external validity (applicability to whole KOA population) or to facilitate favorable enrollment of the study population.)

Could the main outcomes differ significantly between patients with bilateral KOA and those with unilateral KOA?

While treating the more severely affected side, if the severity flipped (i.e., the opposite side became more painful at Visit 7 or Visit 13), could this have influenced the patient-reported outcomes?

If so, is a subgroup analysis or sensitivity analysis excluding bilateral KOA required?

Patients with bilateral KOA are likely to exhibit different weight-bearing patterns during daily activities and may have a worse prognosis or clinical outcome compared to those with unilateral KOA. Please clarify and discuss these aspects.

- If statistical significance between visits occurred at Visit 13 but not at Visit 7, does this imply that LLLT has a delayed or cumulative effect, whereas conventional acupuncture plays a major role during the early phase?

How would this timeline-related result be explained, in the background of a dose-response effect or the multiple exposures to LLLT and acupuncture treatment?

Furthermore, it is intriguing that 12 sessions of active acupuncture resulted in only a -7 mm difference on the VAS, which falls below the MCID threshold.

While the sham control is well-designed and rigorous, there was an unavoidable lack of practitioner blinding, and this functioned as an active control.

Please discuss the potential bias arising from the practitioners' intentions or expectations when administering the control acupuncture treatment.

- In the final section of the Results: "830 nm ILA group showed a larger mean change in the primary outcome compared with the 650 nm group."

If this pilot trial was aimed at determining superiority between the 830 nm and 650 nm wavelengths, a three-group ANCOVA would not directly compare the head-to-head difference between these two active groups.

I suggest that this statement be rephrased in a more conservative tone, as it is currently based strictly on mean differences without direct intergroup statistical significance.

2) Minor Considerations

- Please consider providing a more detailed visual representation of the major outcomes would be beneficial for readers (e.g. a raincloud plot) rather than presenting them solely in the current data matrix format.

- This pilot study introduces the intervention as a specific type of LLLT in the background section. However, since the control group was designed under the premise that active acupuncture is already effective for KOA (without an inactive/placebo control), the authors should provide the underlying evidence for acupuncture's efficacy in the Introduction (or discussion) so that readers can fully understand the rationale behind this control setting.

- Please ensure the phrase "VAS during activity" is defined clearly for the readers. Does it refer to walking under weight-bearing conditions or the active range of motion of the joint? "Activity" can vary widely in terms of tasks and intensity.

- p.14, line 254: Please change "effectiveness" to "efficacy".

- Table 1: Please review the table, as it contains two duplicated rows for the VAS. Additionally, VAS data and vital sign surveillance would be more appropriately placed in the assessment/safety section.

- Table 2: Please change the "Dependent" column header to "Dependent variable".

**********

7. PLOS authors have the option to publish the peer review history of their article (what does this mean?). If published, this will include your full peer review and any attached files.

If you choose “no”, your identity will remain anonymous but your review may still be made public.

**Do you want your identity to be public for this peer review?**  For information about this choice, including consent withdrawal, please see our Privacy Policy.

Reviewer #1: No

Reviewer #6: No

Reviewer #7: No

Reviewer #8: No

**********

[NOTE: If reviewer comments were submitted as an attachment file, they will be attached to this email and accessible via the submission site. Please log into your account, locate the manuscript record, and check for the action link "View Attachments". If this link does not appear, there are no attachment files.]

To ensure your figures meet our technical requirements, please review our figure guidelines: https://journals.plos.org/plosone/s/figures

You may also use PLOS’s free figure tool, NAAS, to help you prepare publication quality figures: https://journals.plos.org/plosone/s/figures#loc-tools-for-figure-preparation.

NAAS will assess whether your figures meet our technical requirements by comparing each figure against our figure specifications.

# Author comment: Author response to Decision Letter 4

**DOI:** 10.1371/journal.pone.0353654.r008

19 Jun 2026

POINT-BY-POINT RESPONSES

Dear editor and reviewers

We thank you for reviewing our manuscript and for your constructive comments, which have helped improve the manuscript. We have carefully reviewed the comments and have accordingly revised the manuscript. Changes are tracked in the revised manuscript. Below is a detailed, point-by-point response to your comments. We hope that our revisions meet your expectations.

Additional Editor Comments:

Could you please address the issues raised by the reviewer?

Response:

We sincerely thank the Academic Editor for handling our manuscript across multiple revision rounds. We have carefully addressed each of the points raised by Reviewer #8, including those regarding sample size and statistical power, multiple comparisons, interpretation of findings, randomization and allocation concealment, blinding, clinical relevance, biological plausibility, and the minor comments. Detailed responses to each comment, along with the corresponding revisions to the manuscript, are provided below. We hope that the revised manuscript and our responses adequately address all concerns raised during this revision.

Review Comments to the Author

Reviewer #1:

The authors have fully addressed each of my comments and concerns, I do not have any other questions.

Response:

We sincerely thank you for the careful review and for confirming that our previous responses have adequately addressed all concerns.

Reviewer #6:

Thank you for the detailed revision and thoughtful point-by-point responses. The manuscript has improved substantially, addressing all comments of reviewers.

Response:

We sincerely thank you for your positive feedback and for recognizing the improvements made to the manuscript.

Reviewer #8:

As an ad-hoc reviewer invited during the third revision, I have focused my evaluation on the remaining scientific soundness, methodological queries, and logical structure of the manuscript.

1. Strengths

KOA is a major degenerative disorder affecting a significant portion of the population.

This pilot trial is well-designed to evaluate the feasibility of two different wavelengths of laser acupuncture, utilizing active acupuncture as a sham laser control for KOA patients.

The efficient delivery of laser treatment via acupuncture needles is a notable strength of this study. It suggests a potential for reducing KOA-related pain and improving QoL, thereby contributing valuable insights to the field of KOA treatment.

I hope this study, along with further follow-up research on ILA, will offer significant clinical relevance, impact, and a novel integrative approach to KOA management.

Throughout the initial and revised manuscripts, the authors have demonstrated transparency and scientific rigor by providing detailed CONSORT and STRICTA-compliant methods, comprehensive supplementary materials (including the study protocol and individual data), and plausible biological mechanisms to ensure the robustness of the trial.

Based on my review of the manuscript, I would like to provide some recommendations to further improve the paper.

Response:

We sincerely thank the reviewer for the careful and constructive evaluation of our manuscript, and for recognizing the strengths of this study, including its design, the efficient delivery of laser treatment through acupuncture needles, and the transparency of our reporting. We have carefully addressed the recommendations below.

2. Weaknesses and Suggestions

1) Major Issues

The concern regarding the inflation of the Type I error rate due to multiple comparisons of the same outcome across a continuous time series against the baseline remains unaddressed.

Although the authors previously responded to a similar point by expanding the limitations section, and this exploratory perspective of this pilot trial is understood and acceptable to some extent, the statistical validity of the findings may still be compromised by this inflated error rate.

Given that the study tracks indicators over time but relies solely on independent, point-by-point ANCOVA at each time interval, please consider utilizing a linear mixed-effects model or repeated measures ANCOVA to evaluate the time-by-group interaction effect if a formal re-analysis is feasible.

If a full re-analysis is realistically difficult at this stage, the statement of this methodological limitation must be further strengthened. Furthermore, the discussion and conclusion must be rephrased in a conservative tone, explicitly cautioning readers that the individual p-values at each visit were not adjusted for multiple temporal comparisons.

Response:

We thank the reviewer for this important comment. To address the concern regarding inflated type I error from repeated visit-wise comparisons, we performed a supplementary repeated-measures ANCOVA evaluating the time-by-group interaction across visits, which accounts for the longitudinal structure rather than testing each visit independently. In this analysis, the time-by-group interaction remained supportive for the primary VAS outcomes, whereas most secondary outcomes were less robust. Because this analysis was not pre-specified, we retained the original results and presented the repeated-measures ANCOVA as S3 Table. We have also revised the Seventh limitation to acknowledge that the visit-wise p-values were not adjusted for multiple temporal comparisons and to describe the significant findings as preliminary and hypothesis-generating rather than confirmatory. In addition, we have revised the Conclusion in a more conservative tone.

<Manuscript>

A supplementary repeated-measures ANCOVA of outcomes is presented in S3 Table. (Page 20, Line 344)

Seventh, this exploratory pilot trial measured multiple outcomes at several time points, and the individual visit-wise p-values in the main ANCOVA analyses were not adjusted for multiple temporal comparisons, raising the possibility of an inflated type I error. To address this limitation, we additionally performed a supplementary repeated-measures ANCOVA to evaluate the visit-by-group interaction across visits. The results supported longitudinal group differences for the primary VAS outcomes, whereas most secondary outcomes were less robust. As this analysis was not pre-specified in the protocol and the study had a limited sample size, these supplementary findings should be regarded as exploratory. More broadly, the statistically significant findings of the main analyses should therefore be interpreted as preliminary and hypothesis-generating rather than confirmatory, and future adequately powered confirmatory trials with pre-specified longitudinal analyses and multiplicity-control strategies are warranted. (Page 27-28, Line 506-517)

In this exploratory pilot trial, ILA at 650 and 830 nm showed preliminary signals of short-term pain reduction among patients with KOA. However, because this was a pilot study with a small sample size and exploratory design, adequately powered, confirmatory trials are warranted to validate these preliminary observations. (Page 29, Line 535-538)

While KOA improvement would be generally expected to influence QoL, the actual improvement in QoL achieved through ILA and conventional acupuncture was limited.

Please discuss the potential ceiling effect of the EQ-5D-5L in this patient population and the limitations of MCID,

and whether EQ-VAS should be considered as complementary outcome to capture more subtle changes for further study?

Response:

We thank the reviewer for this valuable comment. As suggested, we have expanded the Discussion to address the ceiling effect of the EQ-5D-5L, the limitations of established MCID values, and the potential role of the EQ-VAS. Specifically, because our participants had K–L grade 2–3 disease, baseline EQ-5D-5L values were relatively high in all three groups (control, 0.74; 650 nm, 0.75; 830 nm, 0.73), leaving limited room for improvement and raising the possibility of a ceiling effect. We also noted that an established MCID (0.32) was derived only from surgically treated patients, whereas in non-surgical patients the effect sizes were small and no clear MCID could be established; accordingly, established MCID values may not be directly applicable to our non-surgical, high-baseline population. Finally, we noted that incorporating the EQ-VAS as a complementary outcome in future studies may help capture subtle, patient-perceived changes in quality of life.

<Manuscript>

The EQ-5D-5L findings in this trial should be interpreted cautiously. In this K-L grade 2–3 KOA population, baseline EQ-5D-5L scores were relatively high across the three groups (control, 0.74; 650 nm, 0.75; 830 nm, 0.73), leaving limited room for further improvement and raising the possibility of a ceiling effect. A previous study of patients with hip or knee OA established an MCID for improvement of 0.32 in surgically treated patients whose health improved, but among non-surgically managed patients the effect sizes were small and no clear minimal clinically important difference (MCID) exceeding the individual-level minimal detectable change could be established [40]. These findings suggest that EQ-5D-5L MCID estimates vary according to the patient population and treatment context. Accordingly, an established EQ-5D-5L MCID may not be directly applicable to a non-surgical, high-baseline population such as ours, which further limits the interpretation of the modest changes observed. Incorporating a complementary measure such as the EQ-VAS in future studies may help better characterize subtle patient-perceived changes in quality of life. (Page 24-25, Line 435-447)

The authors provided detailed reporting on AEs with V/S and laboratory results, clarifying that no significant changes were observed among the three groups in their Discussion section and Table S3.

However, for more conservative safety evaluation, tracking individual clinical change events (or outlier shifts) is more clinically relevant than merely reporting the intergroup statistical significance of means and p-values of vital signs and lab results.

Response:

We thank the reviewer for this insightful comment. In addition to the existing between-group statistical comparisons (S4A and S4B Tables), we added a description of individual-level shifts (normal at baseline to abnormal at Visit 13) by group (S4C Table). These shifts were minor and not clinically significant, and none were reported as adverse events or considered intervention-related.

<Manuscript>

Detailed changes in the vital signs and laboratory test results, as well as individual-level safety shift, are shown in S4 Table. (Page 21, Line 364-365)

The purpose and impact of including bilateral KOA patients in a unilateral treatment trial: "treated with ILA on one-side (more affected side)"

What was the rationale for including patients with bilateral KOA in this trial? (Please elaborate if this was chosen to enhance external validity (applicability to whole KOA population) or to facilitate favorable enrollment of the study population.)

Response:

We appreciate this valuable observation. The primary rationale was to enhance external validity, as restricting enrollment to unilateral KOA would not reflect the broad KOA population seen in routine clinical practice, where bilateral involvement is common. In addition, as a single-center pilot study with limited trial duration, funding, and recruitment opportunities, not excluding patients with bilateral KOA helped maintain a feasible recruitment pool. Only one pre-specified target knee was treated throughout the trial, and target-knee pain/function outcomes were assessed with reference to this knee.

Could the main outcomes differ significantly between patients with bilateral KOA and those with unilateral KOA?

While treating the more severely affected side, if the severity flipped (i.e., the opposite side became more painful at Visit 7 or Visit 13), could this have influenced the patient-reported outcomes?

Response:

Previous evidence suggests that knee-specific patient-reported pain measures, particularly the WOMAC pain scale, may be affected by crosstalk from co-occurring pain sites, including contralateral knee pain, and that patient-reported physical function in symptomatic KOA may vary according to unilateral versus bilateral knee pain status and contralateral knee pain severity [1,2]. Therefore, the possibility that outcomes may differ between patients with unilateral and bilateral KOA cannot be entirely excluded. In our trial, treatment was confined to a single predefined target knee, and the same target knee was treated throughout the trial. Furthermore, VAS and WOMAC assessments were conducted with reference to that target knee. However, contralateral knee symptoms were not longitudinally recorded; therefore, we could not determine whether a “severity flip” occurred during follow-up. These limitations precluded a formal assessment of whether, and to what extent, symptomatic bilateral involvement may have influenced the outcomes.

If so, is a subgroup analysis or sensitivity analysis excluding bilateral KOA required?

Response:

As symptomatic unilateral/bilateral KOA involvement was not systematically recorded as a study variable, and contralateral knee symptoms were not tracked longitudinally, a laterality-based subgroup or sensitivity analysis, including an analysis excluding bilateral KOA, was not feasible. Moreover, given the small sample size of this exploratory pilot study (n = 15 per group), a formal subgroup or sensitivity analysis would have been underpowered and unlikely to yield interpretable results. Accordingly, we have added this issue as a limitation and noted that future confirmatory trials should prospectively record unilateral/bilateral involvement, collect knee-specific outcomes for both knees, and consider stratification or sensitivity analyses based on laterality.

Patients with bilateral KOA are likely to exhibit different weight-bearing patterns during daily activities and may have a worse prognosis or clinical outcome compared to those with unilateral KOA. Please clarify and discuss these aspects.

Response:

We have added a discussion of the potential biomechanical and prognostic implications of bilateral KOA. Previous gait research in mild-to-moderate KOA reported that between-limb kinematic asymmetry during walking was more prevalent in patients with bilateral symptomatic disease [3], suggesting that bilateral involvement may affect weight-bearing activities. In addition, contralateral knee involvement has been associated with greater structural progression of the ipsilateral knee and a higher subsequent arthroplasty rate [4]. Because unilateral/bilateral KOA status and contralateral symptoms were not systematically recorded in our trial, we could not evaluate these factors directly. We have therefore added this issue as a limitation in the Discussion and noted that future studies should prospectively record laterality, collect knee-specific outcomes for both knees, and consider laterality-based stratification or sensitivity analyses.

Taking the above suggestions together, we have added the following to the limitations section of the manuscript:

Eighth, we did not restrict enrollment to patients with unilateral KOA to reflect real-world clinical practice, while treatment was confined to a single predefined target knee and VAS/WOMAC assessments were conducted for the predefined target knee. However, symptoms in the contralateral knee were not monitored over time. Previous evidence indicates that patient-reported pain and physical function in KOA may be influenced by contralateral knee pain and by unilateral versus bilateral symptom status [44,45]. We therefore could not determine whether the relative severity between the two knees shifted during follow-up, and an influence of the untreated contralateral knee on the patient-reported outcomes cannot be excluded. Prior work also suggests that bilateral involvement may matter clinically: between-limb gait asymmetry has been reported to be more common in bilateral than in unilateral mild-to-moderate KOA [46], and coexisting contralateral KOA has been linked to faster structural progression and a higher subsequent arthroplasty rate in the index knee [47]

Attachment Submitted filename: Response_to_Reviewer_ILA_KOA_R4.docx

# Peer review: Decision Letter 4

**DOI:** 10.1371/journal.pone.0353654.r009

**Authors:** Ramada Rateb Khasawneh

29 Jun 2026

Efficacy and safety of invasive laser acupuncture (650 and 830 nm) on knee osteoarthritis: A pilot randomized clinical trial

PONE-D-25-31350R4

Dear Dr. Kim,

We’re pleased to inform you that your manuscript has been judged scientifically suitable for publication and will be formally accepted for publication once it meets all outstanding technical requirements.

Within one week, you’ll receive an e-mail detailing the required amendments. When these have been addressed, you’ll receive a formal acceptance letter and your manuscript will be scheduled for publication.

An invoice will be generated when your article is formally accepted. Please note, if your institution has a publishing partnership with PLOS and your article meets the relevant criteria, all or part of your publication costs will be covered. Please make sure your user information is up-to-date by logging into Editorial Manager at Editorial Manager® and clicking the ‘Update My Information' link at the top of the page. For questions related to billing, please contact billing support.

If your institution or institutions have a press office, please notify them about your upcoming paper to help maximize its impact. If they’ll be preparing press materials, please inform our press team as soon as possible -- no later than 48 hours after receiving the formal acceptance. Your manuscript will remain under strict press embargo until 2 pm Eastern Time on the date of publication. For more information, please contact onepress@plos.org.

Kind regards,

Ramada Rateb Khasawneh

Academic Editor

PLOS One

Additional Editor Comments (optional):

Good Luck

Reviewers' comments:

Reviewer's Responses to Questions

**Comments to the Author**

1. If the authors have adequately addressed your comments raised in a previous round of review and you feel that this manuscript is now acceptable for publication, you may indicate that here to bypass the “Comments to the Author” section, enter your conflict of interest statement in the “Confidential to Editor” section, and submit your "Accept" recommendation.

Reviewer #8: All comments have been addressed

**********

2. Is the manuscript technically sound, and do the data support the conclusions?

The manuscript must describe a technically sound piece of scientific research with data that supports the conclusions. Experiments must have been conducted rigorously, with appropriate controls, replication, and sample sizes. The conclusions must be drawn appropriately based on the data presented.

Reviewer #8: Yes

**********

3. Has the statistical analysis been performed appropriately and rigorously?

Reviewer #8: Yes

**********

4. Have the authors made all data underlying the findings in their manuscript fully available?

The PLOS Data policy requires authors to make all data underlying the findings described in their manuscript fully available without restriction, with rare exception (please refer to the Data Availability Statement in the manuscript PDF file). The data should be provided as part of the manuscript or its supporting information, or deposited to a public repository. For example, in addition to summary statistics, the data points behind means, medians and variance measures should be available. If there are restrictions on publicly sharing data—e.g. participant privacy or use of data from a third party—those must be specified.

Reviewer #8: Yes

**********

5. Is the manuscript presented in an intelligible fashion and written in standard English?

PLOS ONE does not copyedit accepted manuscripts, so the language in submitted articles must be clear, correct, and unambiguous. Any typographical or grammatical errors should be corrected at revision, so please note any specific errors here.

Reviewer #8: Yes

**********

6. Review Comments to the Author

Please use the space provided to explain your answers to the questions above. You may also include additional comments for the author, including concerns about dual publication, research ethics, or publication ethics. (Please upload your review as an attachment if it exceeds 20,000 characters)

Reviewer #8: #. Response to Revision

- Thank you for your hard work on the revised manuscript, particularly regarding the supplementary plot visualizations, post-hoc rmANCOVA, transparent data reporting, expanded discussions with cautious interpretations, and thorough responses to the previous comments.

The authors have put tremendous effort into providing data-supported conclusions and methodological transparency, and have proactively addressed the points previously suggested.

- I fully understand the authors' point that the eligibility criteria for KOA patients were appropriate given that this trial needed to secure external validity and feasibility as a preliminary clinical study, rather than a strictly controlled experimental setup. Furthermore, the authors have expanded their discussion by referencing related literature on the potential crosstalk from contralateral pain.

It is noted that the control group was not designed to demonstrate or compare the full effect of active acupuncture, but rather to serve as a sham ILA. It was my oversight to misunderstand the control as an active control. Thank you for your response.

While practitioner open-blinding was inevitable, it was successfully minimized by securing assessor, patient, and allocator blinding. Although the authors noted that "Formal assessment of blinding effectiveness using a validated blinding index was not performed in this pilot trial" for this trial, it would be recommended to include a blinding index test in future studies to avoid similar concerns.

- From a clinical perspective, I recommend this research as it elucidates an efficient and minimally invasive ILA approach for KOA patients suffering from poor long-term prognosis and deteriorating QoL, and thus would contribute to the field of integrative medicine.

I hope the authors' upcoming confirmatory clinical trial based on this pilot study will be successful, utilizing increased statistical power and a refined, focused protocol.

**********

7. PLOS authors have the option to publish the peer review history of their article (what does this mean?). If published, this will include your full peer review and any attached files.

If you choose “no”, your identity will remain anonymous but your review may still be made public.

**Do you want your identity to be public for this peer review?**  For information about this choice, including consent withdrawal, please see our Privacy Policy.

Reviewer #8: No

**********

# Editor report: Acceptance letter

**DOI:** 10.1371/journal.pone.0353654.r010

**Authors:** Ramada Rateb Khasawneh

PONE-D-25-31350R4

PLOS One

Dear Dr. Kim,

I'm pleased to inform you that your manuscript has been deemed suitable for publication in PLOS One. Congratulations! Your manuscript is now being handed over to our production team.

At this stage, our production department will prepare your paper for publication. This includes ensuring the following:

* All references, tables, and figures are properly cited

* All relevant supporting information is included in the manuscript submission,

* There are no issues that prevent the paper from being properly typeset

You will receive further instructions from the production team, including instructions on how to review your proof when it is ready. Please keep in mind that we are working through a large volume of accepted articles, so please give us a few days to review your paper and let you know the next and final steps.

Lastly, if your institution or institutions have a press office, please let them know about your upcoming paper now to help maximize its impact. If they'll be preparing press materials, please inform our press team within the next 48 hours. Your manuscript will remain under strict press embargo until 2 pm Eastern Time on the date of publication. For more information, please contact onepress@plos.org.

You will receive an invoice from PLOS for your publication fee after your manuscript has reached the completed accept phase. If you receive an email requesting payment before acceptance or for any other service, this may be a phishing scheme. Learn how to identify phishing emails and protect your accounts at https://explore.plos.org/phishing.

If we can help with anything else, please email us at customercare@plos.org.

Thank you for submitting your work to PLOS ONE and supporting open access.

Kind regards,

PLOS ONE Editorial Office Staff

on behalf of

Dr. Ramada Rateb Khasawneh

Academic Editor

PLOS One
